# A standardized framework for robust fragmentomic feature extraction from cell-free DNA sequencing data

**DOI:** 10.1186/s13059-025-03607-5

**Published:** 2025-05-23

**Authors:** Haichao Wang, Paulius D. Mennea, Yu Kiu Elkie Chan, Zhao Cheng, Maria C. Neofytou, Arif Anwer Surani, Aadhitthya Vijayaraghavan, Emma-Jane Ditter, Richard Bowers, Matthew D. Eldridge, Dmitry S. Shcherbo, Christopher G. Smith, Florian Markowetz, Wendy N. Cooper, Tommy Kaplan, Nitzan Rosenfeld, Hui Zhao

**Affiliations:** 1 Cancer Research UK Cambridge Institute, University of Cambridge, Li Ka Shing Centre, Robinson Way, Cambridge, CB2 0RE UK; 2 Cancer Research UK Cambridge Centre, University of Cambridge, Li Ka Shing Centre, Robinson Way, Cambridge, CB2 0RE UK; 3 The Centre for Cancer Cell and Molecular Biology, Barts Cancer Institute, Queen Mary University of London, John Vane Science Centre, Charterhouse Square, London, EC1M 6BQ UK; 4 LKS Faculty of Medicine, The University of Hong Kong, Hong Kong SAR, China; 5 Cancer Mechanisms and Biomarkers Research Group, School of Life Sciences, University of Westminster, London, W1 W 6UW UK; 6 School of Computer Science and Engineering, The Hebrew University of Jerusalem, Jerusalem, Israel; 7 Department of Developmental Biology and Cancer Research, Faculty of Medicine, The Hebrew University of Jerusalem, Jerusalem, Israel

**Keywords:** CfDNA, Fragmentomics, Cancer genomics, Feature extraction

## Abstract

Fragmentomics features of cell-free DNA represent promising non-invasive biomarkers for cancer diagnosis. A lack of systematic evaluation of biases in feature quantification hinders the adoption of such applications. We compare features derived from whole-genome sequencing of ten healthy donors using nine library kits and ten data-processing routes and validated in 1182 plasma samples from published studies. Our results clarify the variations from library preparation and feature quantification methods. We design the Trim Align Pipeline and cfDNAPro R package as unified interfaces for data pre-processing, feature extraction, and visualization to standardize multi-modal feature engineering and integration for machine learning.

**Supplementary Information:**

The online version contains supplementary material available at 10.1186/s13059-025-03607-5.

## Background

Cell-free DNA (cfDNA) is naturally shed into body fluids (e.g., blood, urine, and cerebrospinal fluid) via various biological processes [1, 2]. These fragments are relatively short in length (~ 167 bp) and short-lived (half-life of ~ 30 min) and reflect the physiological condition and disease progressing in the host [1, 3]. Utilizing cfDNA from peripheral blood plasma for non-invasive diagnostics has been reported as applicable in various clinical regimes, such as non-invasive prenatal testing (NIPT) [4], urinary tract infection monitoring [5], and genotyping to enable targeted therapy [6]. One of the earliest and broadest applications of liquid biopsy is to detect somatic mutations in cell-free DNA shed by tumors into the bloodstream. Minimal residual disease (MRD) detection commonly utilizes matched tumor tissue for a priori information and often relies on targeted approaches such as whole-exome and capture-panel sequencing (i.e., tumor-informed) [7–12].

However, access to tumor material can be challenging, and the design and optimization of sequencing panels can lead to long turnaround times, posing challenges for clinical applications. In contrast to tumor-informed methods, there is a growing focus on tumor-naive strategies, which have better accessibility and are more feasible for clinical practice as tumor tissue is not required. Accompanied by endeavors to search for better tumor-naive methods, the research field is witnessing an upsurge in multi-modal artificial intelligence (AI) methods for cancer detection, among which cfDNA fragmentation patterns are one of the most promising biomarkers [13–18].

The length of the cfDNA fragments is an informative fragmentomic feature. Plasma cfDNA exhibits specific biological patterns shaped by the physiological conditions in blood circulation. Circulating tumor DNA (ctDNA) has been reported to be shorter than cfDNA fragments derived from healthy tissue [17], a finding which was validated with patient-derived mouse model and signal enrichment by selection of shorter DNA fragments [13, 14]. In addition, interrogation of sequencing coverage in specific genomic regions could also help detect cancer. Various studies reported that coverage and fragment length patterns in transcription factor binding sites (TFBS) and transcription start sites (TSS) could inform cancer detection [15, 16, 18–20].

Furthermore, various studies have investigated and exploited the motif landscapes of cfDNA to detect cancer signal. Jiang et al. reported that patients with hepatocellular carcinoma exhibited a higher fraction of adenine (A) or thymine (T) relative to cytosine (C) and guanine (G) at the 5′ ends of fragments compared to samples from healthy donors [21]. The biological mechanisms were elucidated by studying roles of deoxyribonuclease 1 (DNASE1), deoxyribonuclease 1 like 3 (DNASE1L3), and DNA fragmentation factor subunit beta (DFFB) in the cfDNA fragmentation processes [22]. Fragment motif is increasingly demonstrating its effectiveness in detecting cancer signal as part of multi-modal approaches [23–26].

Unlike solid tissue specimens, there is minute quantity of cfDNA molecules in plasma (5–10 ng/mL) and usually an even lower amount of ctDNAs in the early stage patients [1, 27]. Data derived from cfDNA reflects a comprehensive and heterogeneous spectrum of information from the entire human body [28]. Importantly, considering the specific property of cfDNA molecules, the fragmentomic features might be easily biased by external factors introduced in various pre-analytical, lab experimental and analytical steps, including sample collection [29, 30], cfDNA extraction [31], library preparation, data trimming, genome alignment, and how the fragmentomic features are computationally calculated. The differences caused by the enzymatical and chemical settings in library kits, adapter trimming, local and global genome alignment strategies, and the extraction of biological features become unneglectable in the cfDNA study field [1, 27, 32–34] and software originally designed for analyzing solid tissue sequencing data is suboptimal for cfDNA, raising significant concerns when developing multi-modal AI models for cancer detection [27, 34]. An interpretable and robust feature engineering process is essential, given its pivotal role in creating effective AI models [35].

However, despite being broadly recognized by the research community as a possible confounder, research studies that comprehensively measure how various library preparation protocols and computational pipelines impact the fragmentomic markers are lacking. The calculation of fragmentomic features (e.g., fragment length and motif) requires deeper understanding of library structures and cfDNA-specific considerations. Using fragment length as an example, previous tools designed for tissue sequencing might not work for cfDNA sequencing data [36, 37]. User-friendly software tailored for cfDNA data analysis is in urgent need.

**Fig. 1:**
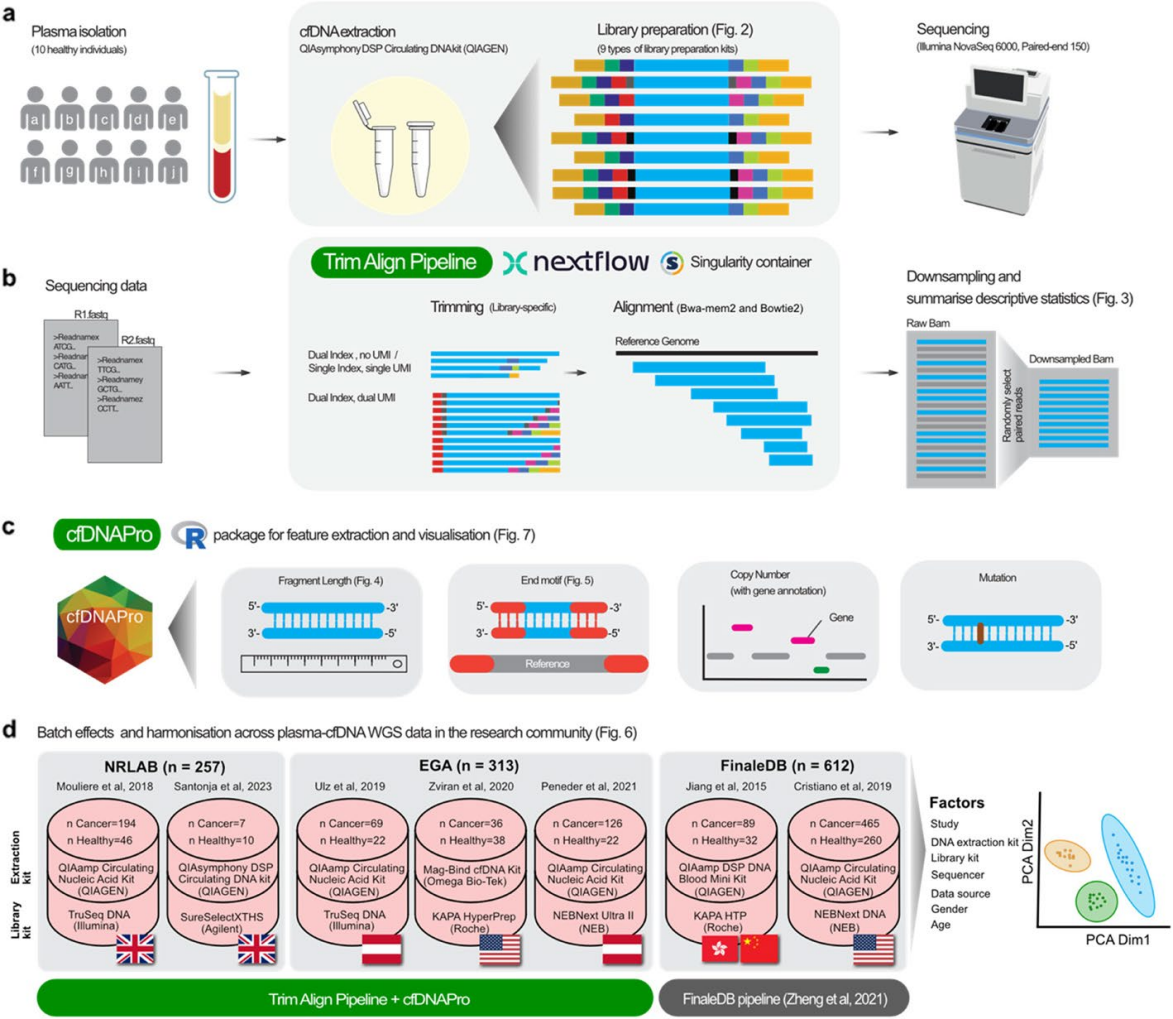
Overview of the study. **a** Plasma samples were collected from 10 healthy donors, cfDNA was extracted using QIAsymphony DSP Circulating DNA Kit (QIAGEN) [41], and independent sequencing libraries were made using 9 different kits (Fig. 2 and Additional file 1: Fig. S1). PE 150 bp whole-genome sequencing was performed on Illumina NovaSeq 6000 sequencer. **b** Trimming and alignment of data. The Trimming Alignment Pipeline (TAP) built using Nextflow [42], designed for library-specific sequencing data trimming and cfDNA-specific alignment. All generated bam files were downsampled to 1 × coverage. **c** cfDNAPro R package was written for cfDNA feature calculation and visualization. It offers utilities for extracting fragment length, fragment end motif, copy number, and single nucleotide variations from whole-genome sequencing data of cfDNA. In addition, cfDNAPro allows integrated analysis of features, such as gene location annotation on CNV plot, and separating length or motif distribution by mutations. **d** Healthy and cancer plasma samples were collected from seven published studies (*n* = 1182, Additional file 2: Table S5). For each patient, when multiple samples are available, only sample from earliest timepoint was kept. PCA analysis revealed the batch effects across datasets

For this purpose, we investigated and demonstrated the various biases affecting cfDNA analysis by examining the paired-end (PE) sequencing data of cfDNA fragments We collected plasma specimens from 10 healthy donors and extracted cfDNA using the QIAsymphony DSP Circulating DNA Kit (QIAGEN). This was followed by library preparation (Fig. 1a), sequencing, bioinformatic analysis (Fig. 1b), robust feature extraction with cfDNAPro (Fig. 1c), and controlling for batch effects (Fig. 1d). We report the biases originating from individual samples and library kits and clarified the batch effects among healthy plasma samples derived from published studies. In this paper, we present Trim Align Pipeline (TAP), a new Nextflow pipeline for library-specific trimming and cfDNA-optimized alignment. We also implemented the cfDNA-specific feature extraction methods as “cfDNAPro” R [38] package, providing a user-friendly ensembled tool for comprehensive and reproducible analysis of cfDNA sequencing data. The feature analysis utilities include not only individual fragment length, motif, copy number aberration (CNA), and single nucleotide variations (SNV) feature, but also cross-feature analysis, for example, comparing the length profiles of fragments with and without SNVs. In comparison to existing tools, such as FinaleToolkit [39] and cfDNApipe [40] (Additional file 2: Table S4), TAP and cfDNAPro address the need for library-specific data pre-processing, as well cross-feature analysis in the cutting-edge cfDNA fragmentomic researches. This underpins reproducible and robust research towards multi-modal AI for disease detection. Our study proposed a one-stop solution for processing sequencing data, from FASTQ files to fragmentomics features. We wish TAP and cfDNAPro to provide a catalyst for further improvements in the implementation and development of cfDNA biomarkers.

**Fig. 2:**
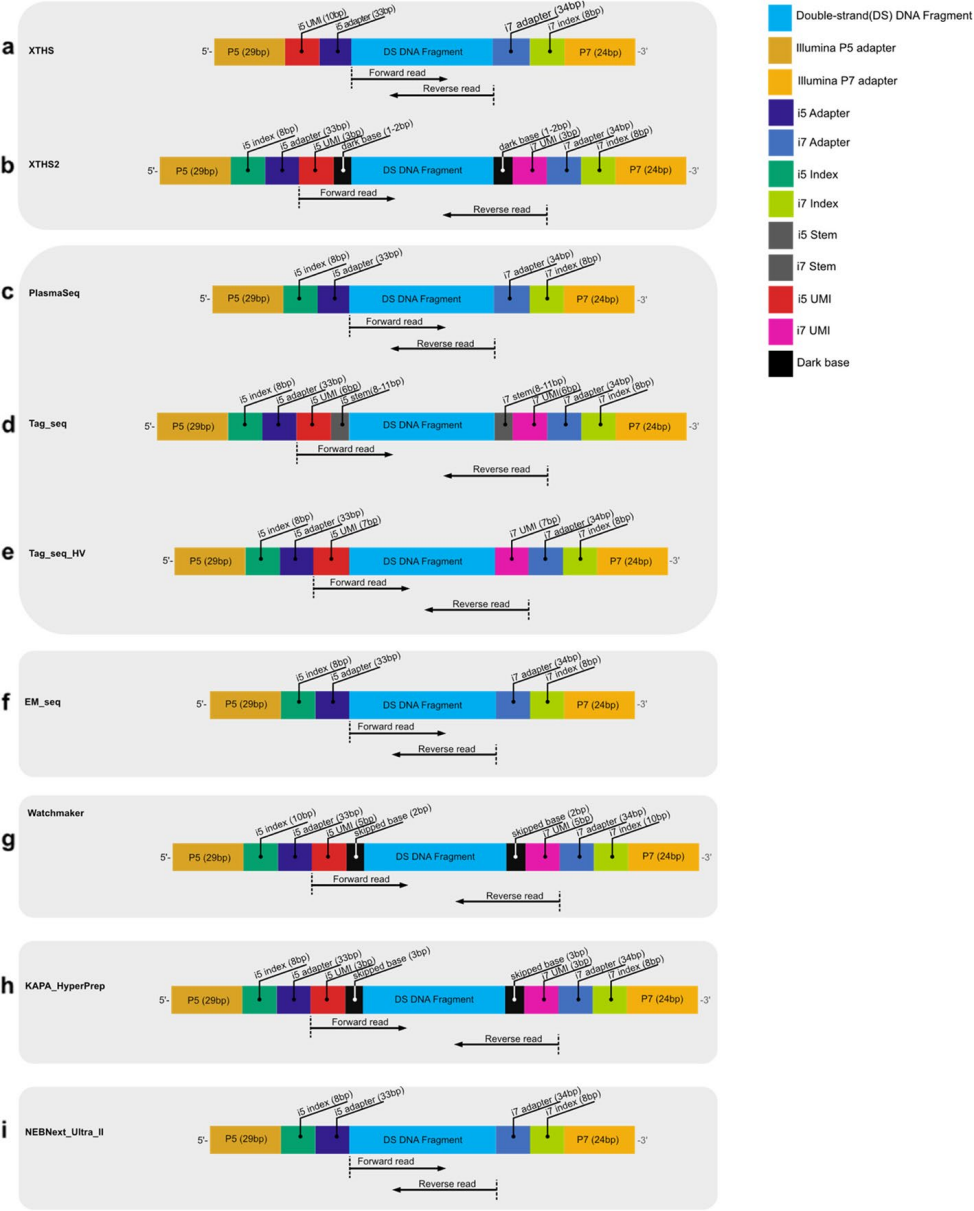
Amplicon structure of different library kits. All libraries are made from double-stranded cfDNA fragments. Kits within the same grey rectangle have the same supplier. **a** XTHS [43] and **b** XTHS2 [44] (Agilent Technologies, Inc.). **c** PlasmaSeq [45],**d** Tag_seq [46], and **e** Tag_seq_HV [47] (Takara Bio Inc.). **f** A library (denoted by “EM_seq” in the manuscript) was made using EM_seq [48] (New England Biolabs), libraries before enzymatic C to T conversion were sequenced. **g** A library (denoted by “Watchmaker” in the manuscript) prepared with adapters from EF 2.0 Library Preparation and Universal Adapter System [49] (Twist Bioscience), and enzymes from Watchmaker [50] (Watchmaker Genomics). **h** KAPA_HyperPrep kits (Roche) [51]. **i** NEBNext_Ultra_II DNA Library Prep Kit for Illumina (New England Biolabs) [52]. The nucleotide sequences of P5/P7 adapter, i5/i7 adapter and i5/i7 stem are shown in Additional file 1: Fig. S1

## Results

### Different library kits exhibited variations in sequencing data properties

We collected plasma specimens from 10 healthy donors and extracted cfDNAs using QIAsymphony DSP Circulating DNA Kit (QIAGEN) [41]. In our study, the general criteria for selecting library kits are as follows: (a) it should be simple to perform capture as the targeted assay is still more sensitive than WGS for the same cost; (b) it should have molecular barcodes; (c) it should be broadly used by the research community. Thus, we chose these nine library kits: ThruPLEX Plasma-Seq (PlasmaSeq) [45] and ThruPLEX Tag-Seq (Tag_seq) [46] are the kits constantly used by the in-house experiments. ThruPLEX Tag-Seq HV (Tag_seq_HV) [47] is a newer version of Tag_seq; it accepts larger volume of plasma DNA as input which facilitates analysis when the samples are less concentrated. Based on previous experiences [53], SureSelect XT HS (XTHS) [43] could achieve high sensitivity with low input and is more amenable to capture than ThruPLEX kits. However, it does not have dual sample barcodes, which suffers from index hopping issues. In contrast, SureSelect XT HS2 (XTHS2) [44] has dual sample barcodes and dual molecular barcodes and easy capture steps for targeted sequencing. NEBNext Enzymatic Methyl-seq (EM_seq) [48] is popular in methylation studies in the cfDNA research area. Multi-omics AI combining different features (e.g., fragmentomics and methylome) is broadly studied. We wish to evaluate the fragmentomics features derived from this EM_seq kit to offer guidance for multi-omic studies. Kapa HyperPrep (KAPA_HyperPrep) [51] and NEBNext Ultra II DNA Library Prep Kit for Illumina (NEBNext_Ultra_II) [52] are broadly used by the research community. To further increase the diversity, we have also added Watchmaker DNA Library Prep Kit for Fragmented Double-Stranded DNA (Watchmaker) to the analysis pool.

**Fig. 3:**
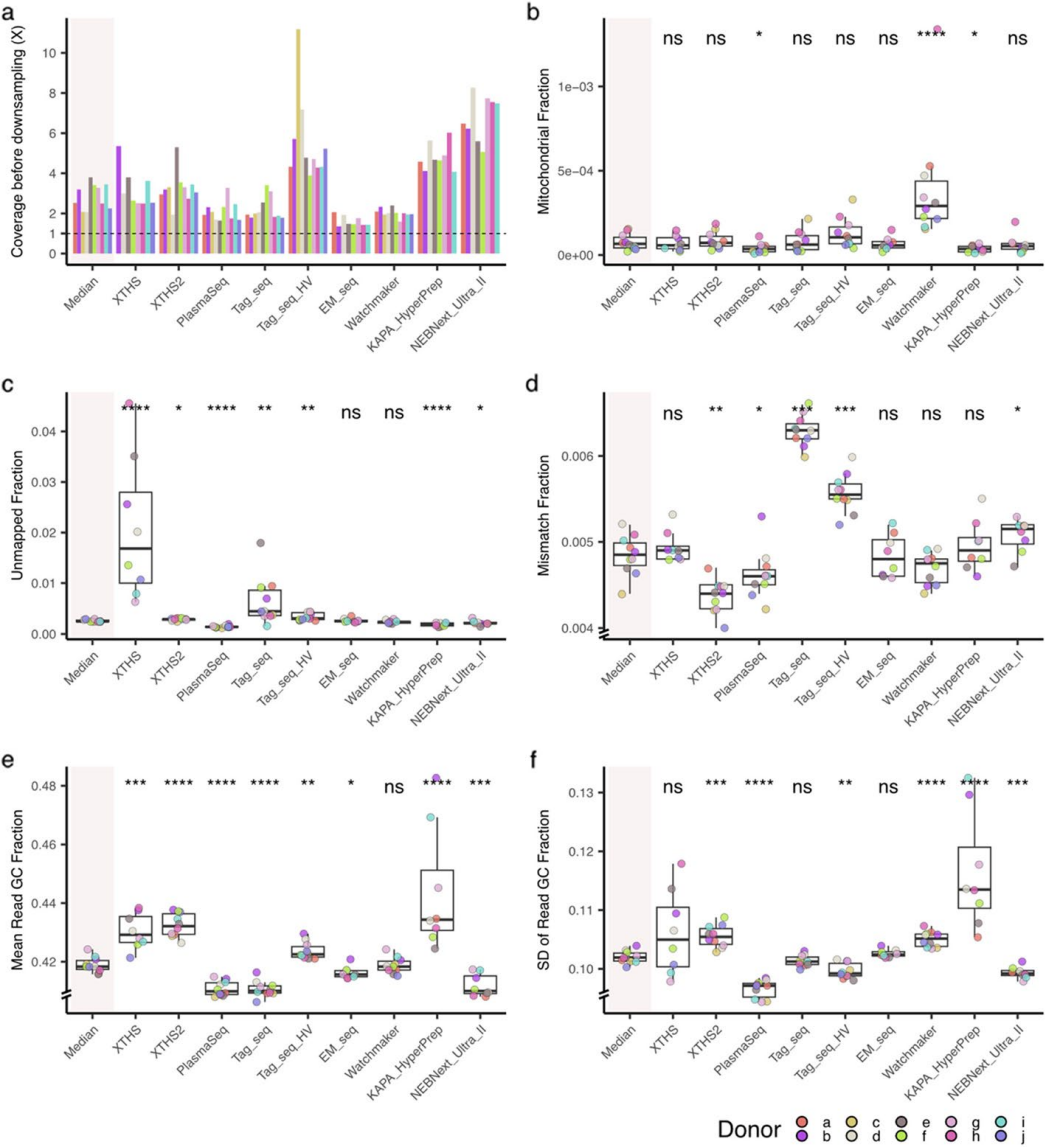
Sequencing data statistics. The metrics of each library kit group were compared with the median values (i.e., the median value of each donor across all library kits). **a** Raw sequencing coverage. All samples were downsampled to 1 × as indicated by horizontal dash line. Statistics shown in other panels were based on downsampled BAM files. **b** The fraction of mitochondrial reads. **c** Fraction of unmapped reads. **d** Fraction of mismatched bases. **e** Mean GC content per read. **f** Standard deviation (SD) of GC content of reads. Wilcoxon test (two-sided) was used for all statistical comparisons. ns: *p* > 0.05, *: *p* ≤ 0.05, **: *p* ≤ 0.01, ***: *p* ≤ 0.001, ****: *p* ≤ 0.0001

We made 9 different libraries (Fig. 2, Table 1, and Additional file 1: Fig. S1) from 10 healthy donors, followed by PE 150 bp sequencing using Illumina NovaSeq 6000 sequencer (Fig. 1a). Then, we processed sequencing data with 10 different trimming-alignment routes (Table 2), with all generated bams being downsampled to 1x (Fig. 1b). We calculated descriptive metrics of the bam files to evaluate the inherent properties exhibited by different library kits (e.g., the fraction of unmapped reads, mitochondrial reads, GC content) (Fig. 3 and Additional file 1: Fig. S2).

**Table 1:** Library kit characteristics. Further information about extension temperature, extension time, and amplification enzyme is shown in Additional file 2: Table S7

Library kit	Label in paper	Provider	DNA input^a^	Sample barcode	Molecular barcode	Cost^b^	Processing time^c^	PCR cycles^d^
SureSelect XT HS	XTHS	Agilent Technologies	10–200 ng	Single	Single (i5)	£££	~ 4 h	16
SureSelect XT HS2	XTHS2	Agilent Technologies	10–200 ng	Unique dual	Dual	££££	~ 4 h	14
ThruPLEX Plasma-Seq	PlasmaSeq	Takara Bio	1–30 ng	Unique dual	No	££££	~ 2 h	9
ThruPLEX Tag-Seq	Tag_seq	Takara Bio	1–50 ng	Unique dual	Dual	££	~ 2 h	7
ThruPLEX Tag-Seq HV	Tag_seq_HV	Takara Bio	5–200 ng	Unique dual	Dual	££	~ 2 h	16
NEBNext Enzymatic Methyl-seq^e^	EM_seq	New England Biolabs	10–200 ng	Unique dual	No	£££££	~ 2 h	10
Watchmaker DNA Library Prep Kit for Fragmented Double-Stranded DNA^f^	“Watchmaker” in figures and texts	Twist Bioscience and Watchmaker Genomics	0.1–500 ng	Unique dual	Dual	££	~ 4 h	9
Kapa HyperPrep	KAPA_HyperPrep	Roche	10–50 ng	Unique dual	Dual	£££££	~ 22 h	10
NEBNext Ultra II DNA Library Prep Kit for Illumina	NEBNext_Ultra_II	New England Biolabs	0.5–1000 ng	Unique dual	No	£	~ 2 h	8

^a^DNA input recommended by manufacturers

^b^Estimated based on internal laboratory settings. More “£” signs mean higher cost

^c^Estimated according to in-house protocols

^d^PCR cycles used in this study

^e^The library was sent for sequencing before enzymatic conversion

^f^Adapters were from The Twist EF 2.0 Library Preparation and Universal Adapter System, and enzymes were from Watchmaker DNA Library Prep Kit for Fragmented Double-Stranded DNA

**Table 2:**
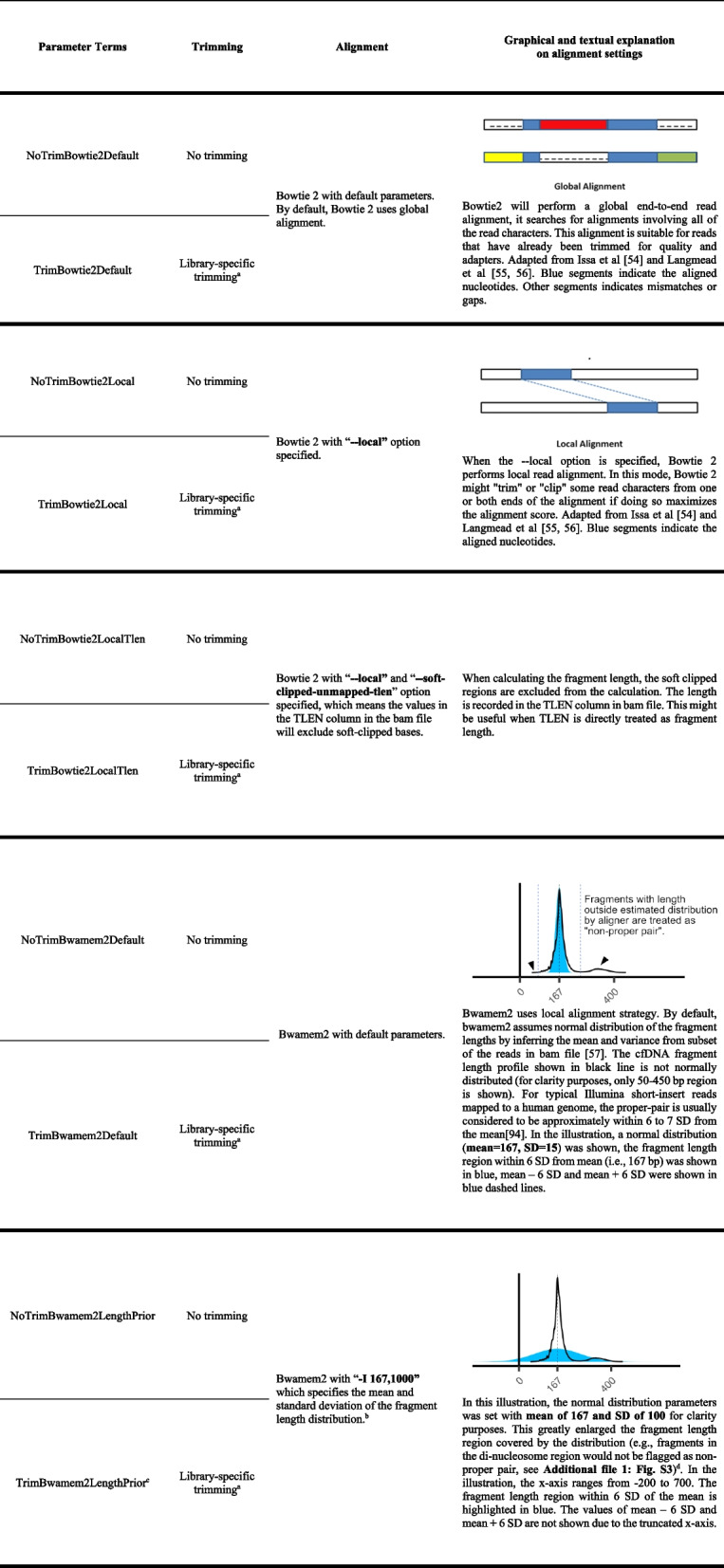
Trimming-alignment parameter settings [54–57]. The version numbers of software used are shown in Additional file 2: Table S2

^a^See Methods, Fig. 2, and Additional file 1: Fig. S1 for a detailed trimming strategy

^b^See Methods and Additional file 1: Fig. S3 for a detailed “proper pair” filtering explanation

^c^In the manuscript, the “TrimBwamem2LengthPrior” was referred to as “optimized” trimming-alignment parameter settings with prior knowledge of fragment length distribution

^d^Although in our study non-proper pair reads were not discarded, we still recommend setting the length prior to minimize the potential issues in other data analyses which might interact with the proper pair flags

Previous studies revealed that the fragmentation pattern of cell-free mitochondrial DNA differs from chromosomal DNA [16], and cancer samples have elevated fragments from mitochondria [14, 58]. We found that the Watchmaker has a median of 0.03% mitochondria reads, which is 4.4 times higher than the median of all library kits (Fig. 3b). This observation is consistent across all analyses routes (Additional file 1: Fig. S2a), which strongly implies the inherent biochemical property of Watchmaker shaped the result. XTHS, XTHS2, Tag_seq, and Tag_seq_HV have a higher number of unmapped reads. XTHS seems to be more variable across donors (Fig. 3c).

In terms of the number of mismatches between sequenced reads and reference genome (Fig. 3d), Tag_seq, Tag_seq_HV, and NEBNext_Ultra_II have more mismatched nucleotides while XTHS2 and PlasmaSeq have fewer. These metrics are useful for evaluating the suitability of a kit for studying mutations together with the Unique Molecular Identifier (UMI). In addition, we analyzed the Mean GC content per read (Fig. 3e). XTHS, XTHS2, Tag_seq_HV, and KAPA_HyperPrep have higher GC content while PlasmaSeq, Tag_seq, EM_seq, and NEBNext_Ultra_II are lower. The standard deviation (SD) of GC content of reads was shown in Fig. 3f. XTHS2, Watchmaker, and KAPA_HyperPrep have higher SD while PlasmaSeq, Tag_seq_HV, and NEBNext_Ultra_II have lower SD. In addition, XTHS and KAPA_HyperPrep kit tend to have a broader distribution. The results strongly indicate the heterogeneity in sequencing data introduced by different library kits.

### Analytical settings and ambiguity over the definition of a “fragment” affect fragment length

To comprehensively evaluate the analytical impacts on length profiles, we designed ten different trimming-alignment routes (Table 2), coupled with two calculation schemes (i.e., “*With problematic fragment length calculation*” and “*With correct fragment length calculation*”).

**Fig. 4:**
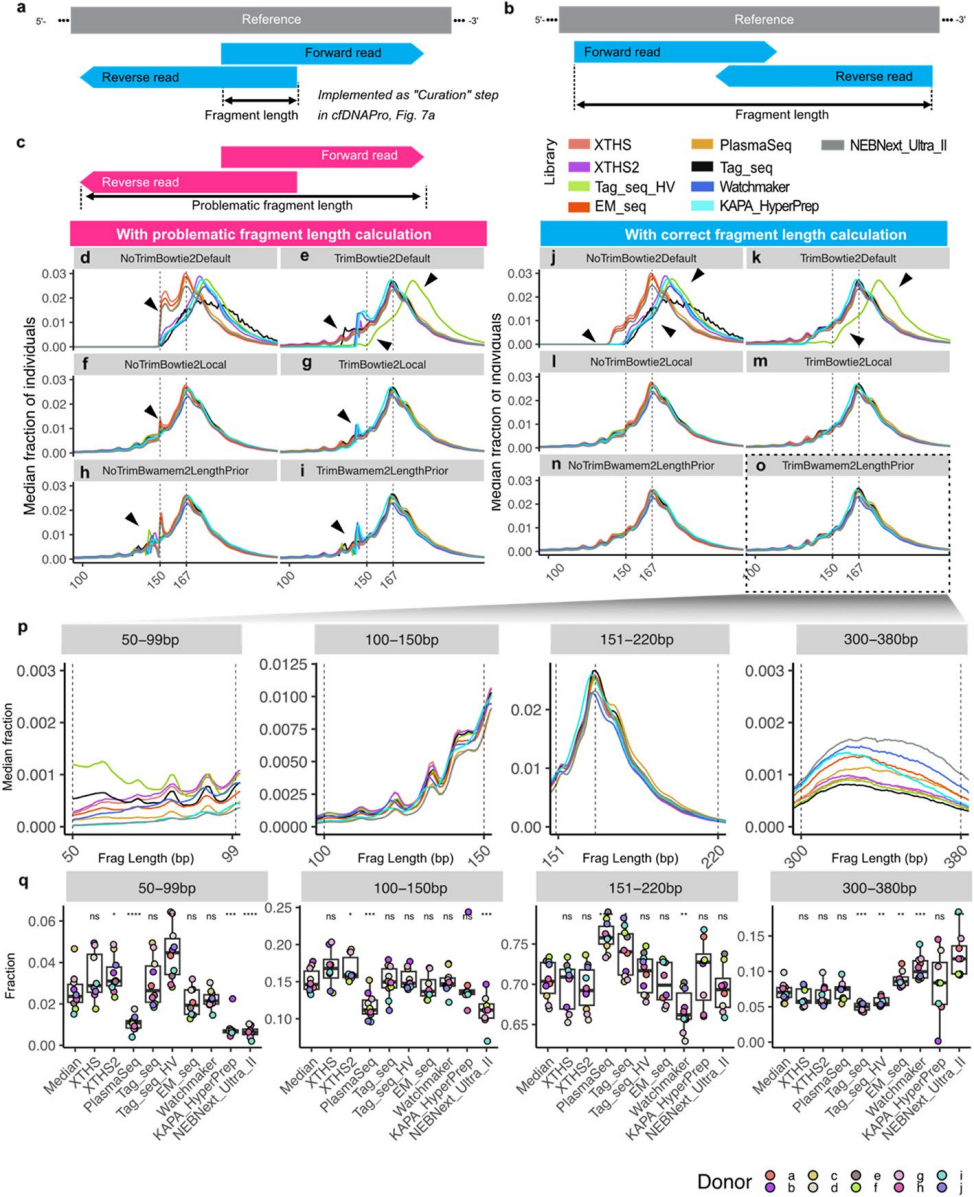
Fragment length definition and analytical impacts. The definition of “fragment length” in this study in ambiguous (**a**) and straightforward (**b**) scenarios. **c** A problematic way to calculate “fragment length.” Median distribution of all donors is shown; each facet shows different trimming-alignment parameters (Table 2). **d**–**i** Fragment length distribution with problematic length calculation. **j**–**o** Fragment length profile with correct fragment length calculation. Black triangles depict areas with artifacts. Fragment lengths were calculated using the *callLength()* implemented in cfDNAPro (Fig. 7a). **p** Fragment length distribution (median of all donors) of four ranges (50–59 bp, 100–150 bp, 151–220 bp, and 300–380 bp) calculated using TrimBwamem2LengthPrior settings. **q** For each donor using each library, sum of fraction in length ranges are shown. ns: *p* > 0.05, *: *p* ≤ 0.05, **: *p* ≤ 0.01, ***: *p* ≤ 0.001, ****: *p* ≤ 0.0001

Our study addressed the ambiguity in defining cfDNA fragments from PE sequencing data. This is essential as aligners and data processing tools adopt various definitions of a “fragment” in paired-end sequencing data, raising concerns in previous study [36]. For properly paired reads with overlapping sequences, there are two scenarios: (1) an ambiguous case occurs when there are sequence-through issues. We propose that the cfDNA fragment is the region between the left boundary of the forward strand and the right boundary of the reverse strand (Fig. 4a); this function is implemented in the cfDNAPro R package (Fig. 7a). In contrast, a problematic way to extract the fragment length is the region between the outermost boundaries (Fig. 4c). (2) A more straightforward case is when the fragments are longer than the read lengths. In this case, the cfDNA fragment is defined as the entire region read pairs cover (Fig. 4b).

For clarity purposes, six out of the ten settings are shown in Fig. 4. Results from all analytical settings are shown in Additional file 1: Fig. S6. In the absence of a correct length calculation (i.e., without using the curation step implemented in cfDNAPro R package) (Fig. 4d–i), the effect of library-specific trimming (Fig. 4e, g, i) can be observed as artifacts (highlighted by black triangles) that are attenuated in contrast to those without trimming (Fig. 4d, f, and h). For example, when the calculation is problematic, there is a paucity of reads below 150 bp in XTHS, EM_seq, and PlasmaSeq. Peaks around 140 bp in Watchmaker, Tag-Seq HV, and KAPA_HyperPrep are no longer present with the correct calculation of fragment lengths (Additional file 1: Fig. S7). Additionally, for those (Fig. 4d and e) with Bowtie2 default settings, profiles were highly heterogeneous, regardless of trimming. We further quantified the fraction of ambiguous read pairs in bam files and found that library-specific trimming could reduce the abundance of ambiguous scenarios, which correlates with the artifacts in fragment length profiles (Additional file 1: Fig. S10).

When correct fragment length calculation is applied, alignment profiles improve across most conditions. While Bowtie2 default settings remain problematic (Fig. 4j and k), the remaining settings yield homogenous and expected fragment length distributions, highlighting the robustness of the curation step. We also compared the feature distributions across different dimensions: fragment length distribution of each healthy donor with each trimming-alignment parameter was shown in Additional file 1: Fig. S4 (problematic length calculation) and Additional file 1: Fig. S5 (correct length calculation), respectively. For each library kit, a comparison of the ten trimming-alignment combinations is shown in Additional file 1: Fig. S7; in addition, for each library kit, an intra-individual comparison of fragment lengths can be found in Additional file 1: Fig. S8, while using the optimized parameter setting (i.e., “TrimBwamem2LengthPrior”), individuals showed highly similar length profiles. For each individual, an inter-kit comparison could be found in Additional file 1: Fig. S9: similarly, when using the optimized parameter setting and the correct fragment length definition implemented in cfDNAPro, library kits exhibited similar fragment length distributions.

Different library kits exhibit variations in fragment length distribution across different regions (Fig. 4p). We inspected four length ranges (i.e., 50–99 bp, 100–150 bp, 151–220 bp, and 300–380 bp) captured by various library kits derived from the optimized analytical settings in Fig. 4o. Tag_seq_HV tends to capture higher proportion of fragments in 50–99 bp region. While PlasmaSeq has lower fraction of 50–99 bp and 100–150 bp fragments, it captures a higher number of 151–220 bp fragments. Furthermore, EM_seq, Watchmaker, and NEBNext_Ultra_II have a higher fraction of fragments in the di-nucleosome region (300–380 bp). Our findings strongly suggest the choice of library kits should be carefully considered when comparing the fragment length signals between healthy control and cancer cohorts.

### Library kits exhibit inherent biases in motif profiles

**Fig. 5:**
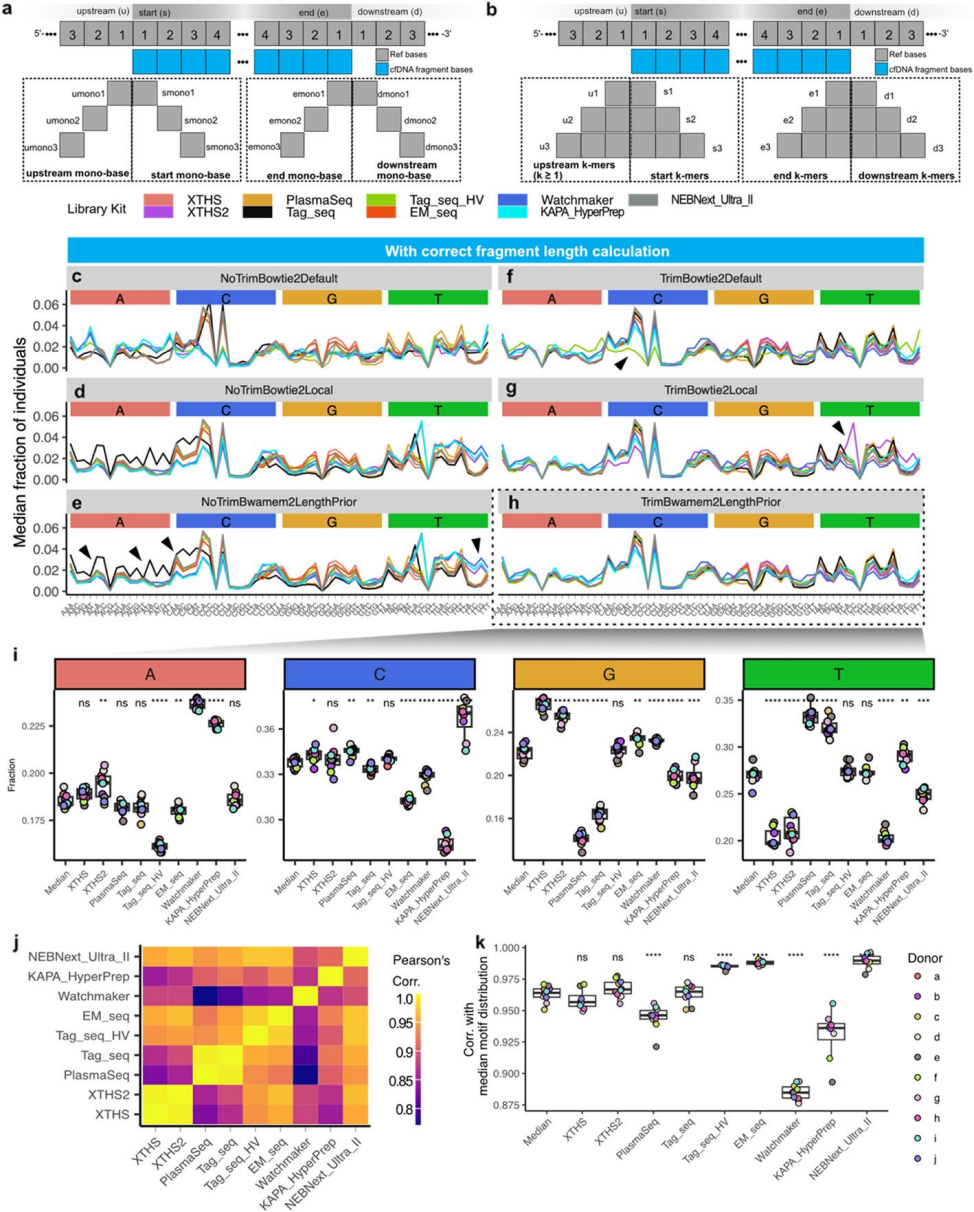
Fragment end motif definitions and variation comparison. **a**–**b** Definitions of eight types of motifs. **c**–**h** Line plots showing “s3” motifs frequency with and without correct fragment definition. **c**–**e** Panels on the left are results derived from analyses without trimming steps. **f**–**h** The right panels are the results of library-specific adapter trimming. All results shown here are those with correct fragment definition (Fig. 4a). Black triangles highlighted examples of abnormal s3 motifs regions for Tag_seq and Watchmaker. **i** Sum of fractions of motif starting with A, C, G, and T in **h**. **j** Pairwise correlation between lines in **h**. **k** Correlation between each donor’s motif profile and the median s3 motif distribution across all donors. ns: *p* > 0.05, *: *p* ≤ 0.05, **: *p* ≤ 0.01, ***: *p* ≤ 0.001, ****: *p* ≤ 0.0001

To evaluate the frequency of various fragment motif across healthy donors, library kits, and trimming-alignment parameters, we defined eight types of fragment motif including two categories. First, mono-nucleotide at various positions relative to the aligned fragment: “umono” at upstream, “smono” at the start, “emono” at the end, and “dmono” at downstream positions (Fig. 5a). Second, k-mers (k ≥ 1) instead of single base: upstream (u), start (s), end (e), and downstream (d) (Fig. 5b). Throughout this study, s3 motifs were analyzed (i.e., the three bases at the start (s) of each fragment). For clarity purposes, only the results with the correct fragment definition are shown in Fig. 5c–h. Results with and without correct fragment length calculation, using ten analytical settings, were shown in Additional file 1: Figs. S16 and S17.

Trimming reduced the biases in motifs starting with A and C in Tag_seq (e.g., Fig. 5e vs h highlighted by black triangles). The optimized setting (Fig. 5h) achieves a relatively homogenous and expected motif distribution. We quantified the fragment starting with A, C, G, and T and found significant variations across different library kits (Fig. 5i). We further calculated the pairwise correlation between s3 motif distributions (Fig. 5j) and the correlation between each library kit and the median s3 motif distribution of all donors analyzed using an optimized setting (Fig. 5k). XTHS and XTHS2 are highly similar, as well as PlasmaSeq and Tag_seq.

The s3 motif distribution of each healthy donor with each trimming-alignment parameter was shown in Additional file 1: Fig. S11 (problematic length definition) and Additional file 1: Fig. S12 (correct length calculation). A comparison of various trimming-alignment combinations for each library kit is presented in Additional file 1: Fig. S13. In addition, for each library kit, a comparison of motifs between healthy donors can be found in Additional file 1: Fig. S14. Using the optimal parameter setting (i.e., “TrimBwamem2LengthPrior”), individuals displayed highly similar profiles; for each individual, a comparison between library kits is available in Additional file 1: Fig. S15.

**Fig. 6:**
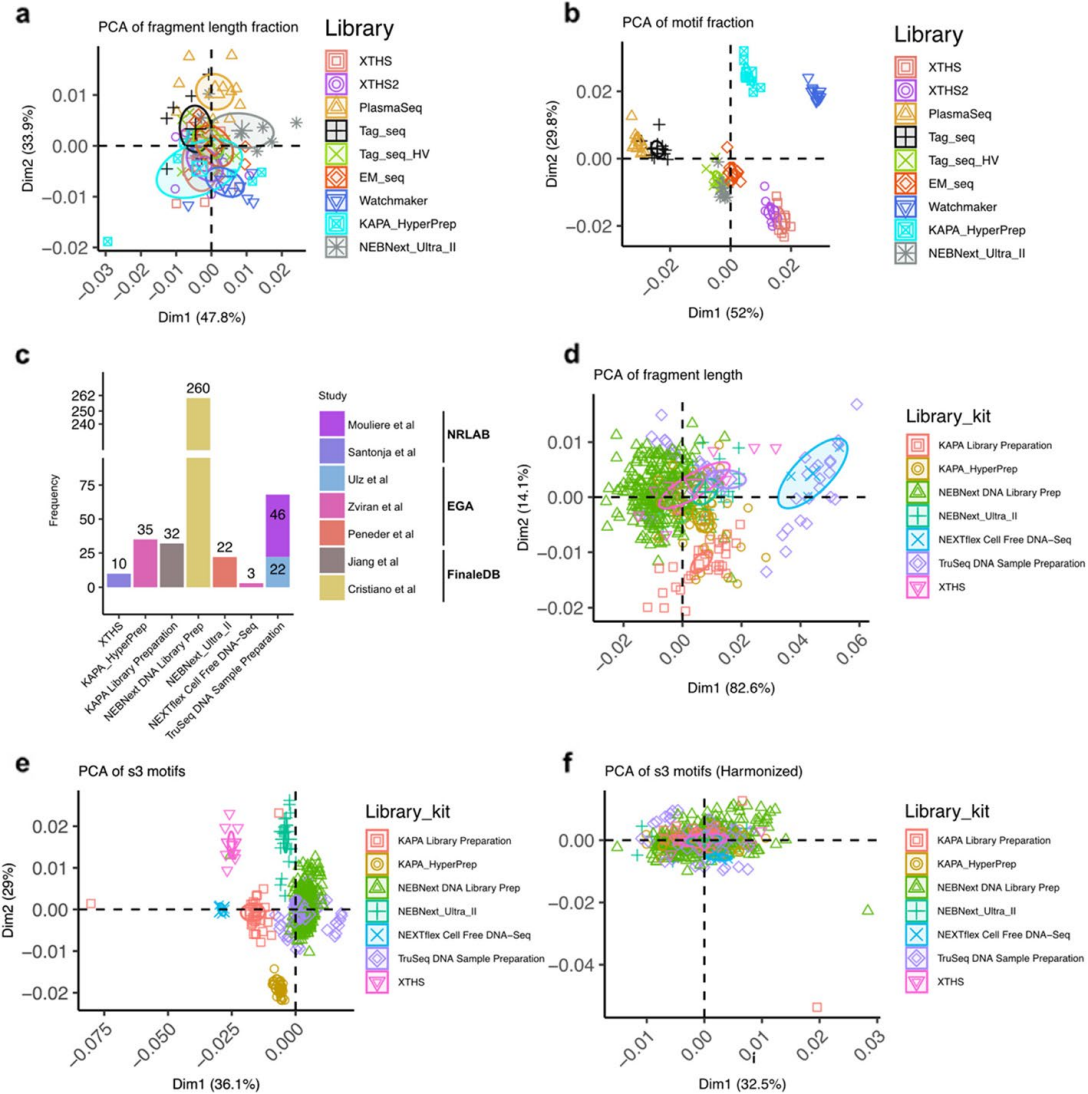
Principal component analysis of length and motif features derived from healthy samples. For each plot, 95% confidence area surrounding the group mean value was shown by ellipses. **a** The PCA analysis of fragment lengths. **b** PCA analysis of fragment s3 motifs. **c** The number of healthy plasma samples derived from published studies. **d** PCA analysis of fragment lengths and grouped by library kit. **e** PCA of s3 motifs of samples from various studies and grouped by library kit. **f** PCA of harmonized s3 motifs

To check if inter-donor and inter-library batch effects exist, we performed PCA of fragment length and s3 motif distributions retrieved from optimized parameter settings (i.e., TrimBwamem2LengthPrior). The results indicated that while fragment length is less affected by library preparation methods (Fig. 6a), the motifs are highly clustered based on the libraries (Fig. 6b). This phenomenon is consistent with previous observations (Fig. 4o, Additional file 1: Fig. S9b, Fig. 5h, and Additional file 1: Fig. S15b). Inter-donor variations affected fragment length and s3 motifs less (Additional file 1: Fig. S31).

### Harmonization attenuates batch effects in WGS data from the research community

We collected 430 healthy plasma samples from seven studies, which used various DNA extraction and library kits (Fig. 1d). We analyzed fragment length and s3 motif (Additional file 1: Fig. S20 and Additional file 1: Fig. S21) of these samples together with potential bias factors: PCA was performed and grouped by library kit (Fig. 6d–e), DNA extraction kit (Additional file 1: Fig. S22a, c, and e), sequencing platforms (Additional file 1: Fig. S22b, d, and f), study group (Additional file 1: Fig. S23a, c, and e), data source (Additional file 1: Fig. S23b, d, and f), gender (Additional file 1: Fig. S24a, b, and c), and age (Additional file 1: Fig. S24 d). For samples with raw data available (Mouliere et al. [13], Santonja et al. [53], Ulz et al. [20], Zviran et al. [59], Peneder et al. [15]), we applied our optimized analytical settings to trim and align the FASTQ files and extracted s3 motif and length features using cfDNAPro. For those from FinaleDB (Jiang et al. [17] and Cristiano et al. [14]), we derived the features based on the alignment coordinates of fragments retrieved from the database [60]. Batch effects were observed in the published datasets (Fig. 6d–e). We conducted harmonization of the input data using the *ComBat_seq()* function from the *sva* R package (version 3.50.0) [61]. The *ComBat()* method in *sva* package adjusts for known batch effects using an empirical Bayesian framework [62]; *ComBat_seq()* [63] implements an improved model based on the “ComBat” framework, which uses a negative binomial regression to model the input count matrix, and estimates parameters representing the batch effects. The adjusted data preserve the integer nature of the input while removing the known batch effects. It can preserve the signals from biological variables (e.g., case or control) specified by users in the adjusted data. Our results indicate this is a potential method for removing batch effects (Fig. 6f, Additional file 1: Figs. S27 and S28), but it is subject to further evaluation in different study designs before the adoption of batch-effects removal. We also analyzed data without samples from FinaleDB due to its different processing pipeline [60], and the results are consistent with those analyzed with FinaleDB (Additional file 1: Figs. S25, S26, S29, and S30). To further inspect if the harmonization process could preserve cancer signals, we collated sWGS data from 752 cancer patients (Additional file 2: Table S5). We stratified the cancer samples into three categories based on the tumor fraction (TF) inferred using ichorCNA [64]: [0, 0.03), [0.03, 0.1), and [0.1, 1]. Square brackets indicate boundary inclusivity, while parentheses indicate boundary exclusivity. As expected, the cancer signals were preserved during harmonization (Additional file 1: Figs. S32 and S33). Early-stage cancers with low TF and the differences between these samples and healthy control are subtle; adoption of the harmonization should subject to specific context of different studies.

### cfDNAPro R package ensures standardized fragmentomic multi-feature extraction

**Fig. 7:**
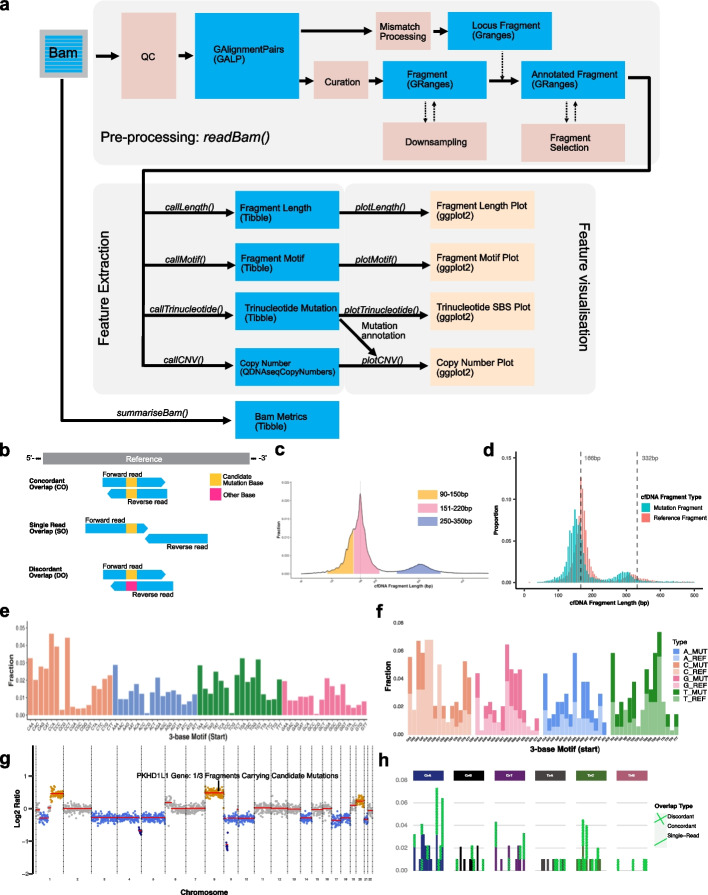
cfDNAPro as an integrated framework for multi-modal analysis. **a** Schematic overview of the cfDNAPro architecture. **b** Three types of SNV mutation overlap scenarios used for mutation quality control in cfDNAPro: Concordant overlap (CO), Single read overlap (SO), and Discordant overlap (DO). **c** Fragment length analysis using the callLength() and plotLength() with highlight length regions of interest. **d** Combining the length and mutation features. **e-f** s3 motif frequency plots with and without fragment stratification by carrying mutations or not. **g** Copy number analysis methods integrated with mutational annotation. Copy number gain, neutral and loss bins were highlighted using orange, grey and blue colours respectively. Bin(s) overlapped with the PKHD1L1 gene are highlighted with the number of mutated fragments and total number of fragments overlapping the gene region. **h** Trinucleotide single base substitution (SBS) profile of a lung cancer patient, stratified by mutationstatus at individual genomic loci. DO substitutions are highlighted with light yellow patterned lines

In light of the highlighted inconsistencies and uncontrolled analytical factors in the earlier sections, we hereby present the open-access R package “cfDNAPro” in which we implemented various cfDNA feature extraction and visualization utilities based on this study. For example, cfDNAPro offers utilities for independent features analysis (e.g., fragment length, motif, SNV, and copy number). In addition, we also developed functions for cross-feature analysis, such as analyzing the length profile of fragments with and without SNVs. The core functions are sample-oriented and can be stratified into three categories: pre-processing, feature extraction, and feature visualization (Fig. 7a). Details functions implemented in cfDNAPro is provided in Table S3. Each section contains functions whose outputs can be piped into the next to reduce memory requirements (details see Methods).

cfDNAPro offers cfDNA-specific feature extraction methods—essentially the QC step implemented in *readBam()* function, which helps attenuate potential biases introduced during various steps (Fig. 4). Moreover, we implemented methods for annotation of mutations of each cfDNA fragment sequenced, defining three categories based on the reference and fragment base status (Fig. 7b): (1) concordant overlap (CO), where both reads support the same variant base; (2) single read overlap (SO), where only one read contains the variant; (3) discordant overlap (DO), where reads disagree. To illustrate how filtering by CO, SO, and DO scenarios can potentially improve the detection of mutation signatures, we removed the DO substitution from the 96 single base substitution (SBS) profile in a lung sample. This adjustment increased the cosine similarity between the cancer sample and SBS4 (COSMIC tobacco smoking signature), from 0.63 to 0.69 (Fig. 7h and Additional file 1: Fig. S19).

Depending on the Bioconductor [65] and Tidyverse ecosystems in R, cfDNAPro is designed to (Fig. 7) support combinatory analysis of cfDNA biological features, making the process more integrative, intuitive, and straightforward. To demonstrate the utility, we conducted analyses on fragment length (Fig. 7c), fragment length categorized mutation-carrying status (Fig. 7d), motif frequency (Fig. 7e), motif frequency stratified by mutation status (Fig. 7f), and CNA annotated with mutation information (Fig. 7g). By standardizing data analysis, cfDNAPro mitigates the analytical impacts on downstream model building.

Figure 7 cfDNAPro as an integrated framework for multi-modal analysis. **a** Schematic overview of the cfDNAPro architecture. **b** Three types of SNV mutation overlap scenarios used for mutation quality control in cfDNAPro: concordant overlap (CO), single read overlap (SO), and discordant overlap (DO). **c** Fragment length analysis using the *callLength()* and *plotLength()* with highlight length regions of interest. **d** Combining the length and mutation features. **e**–**f** s3 motif frequency plots with and without fragment stratification by carrying mutations or not. **g** Copy number analysis methods integrated with mutational annotation. Copy number gain, neutral, and loss bins were highlighted using orange, gray, and blue colors, respectively. Bin(s) overlapped with the PKHD1L1 gene are highlighted with the number of mutated fragments and total number of fragments overlapping the gene region. **h** Trinucleotide single base substitution (SBS) profile of a lung cancer patient, stratified by mutation status at individual genomic loci. DO substitutions are highlighted with light yellow patterned lines.

## Discussion

ctDNA as a non-invasive biomarker for disease detection has gained rapid translational implementation in clinical settings (e.g., cancer early detection [66] and minimal residual disease detection [9, 67]). Despite an increasing number of studies have reported its clinical feasibility, the minute quantity of total cfDNA molecules and usually an even lower amount of ctDNAs in the early stage patients [1, 27] in plasma raised a higher requirement for cancer signal enrichment and noise attenuation. Here, we comprehensively evaluated the experimental (i.e., library preparation) and analytical (i.e., trimming, alignment, and feature extraction) impacts on the length and motif profile. Moreover, we present two analytical tools: TAP (a Nextflow pipeline for library-specific trimming and cfDNA-specific alignment) and cfDNAPro (an R package for feature extraction and visualization).

This study advances the research field in two aspects: it elucidates the bias factors introduced to the data in various steps, serving as an essential reference for researchers in study design; it offers a standardized and scalable one-stop solution for data analysis. Our results add the missing blocks in the current research community and provide critical foundation for future study [29, 31].

To inspect the inherent characteristics of library preparation methods, we chose 9 kits: XTHS and XTHS2 from Agilent Technologies; PlasmaSeq, Tag_seq, and Tag_seq_HV from Takara Bio; EM_seq and NEBNext_Ultra_II from New England Biolabs; Watchmaker from Twist Bioscience and Watchmaker Genomics; and KAPA_HyperPrep from Roche. We evaluated the properties of each kit based on their practical (Table 1) and experimental considerations (Fig. 3).

We discussed the various aspects of experimental concerns, e.g., DNA input, cost, and processing time. PlasmaSeq, Tag_seq, and Watchmaker have relatively lower amounts of required DNA input, which indicates the suitability of these kits for samples with a limited quantity of cfDNA available, for example, finger-prick dry blood spots [68].

Regarding the whole-genome sequencing data generated from various library kits, we found several significantly distinct metrics across libraries that are not negligible. For example, Watchmaker had more mitochondrial reads (Fig. 3b and Additional file 1: Fig. S2a), different library kits generated variable fractions of unmapped reads through different analytical routes we implemented (Fig. 3c and Additional file 1: Fig. S2a). The unmapped reads can be used for downstream analysis in microbial studies [69–71]. While these metrics can inform disease detection, issues caused by batch effects should be considered during the study design phase. When choosing which kit to use, we recommend comprehensively evaluating the scope of the study and candidate library preparation protocols. For example, when aiming at mutation or mismatch analysis, XTHS2 might an appropriate choice (Fig. 3d); similarly, when cost or experiment time become import factors to be evaluated, the information in Table 1 could inform the decision-making process.

Moreover, distinct amplicon structures (Fig. 2 and Additional file 1: Fig. S1) necessitate library-specific trimming. Without this strategy, the results are incongruous (Figs. 4 and 5), rendering downstream feature integration impractical.

To extensively examine analytical impacts, we designed various combinations of trimming and alignment parameter settings (Table 1). To inspect the fragmentomic features of multiple kits, we first clarified the definition of a “fragment” in read alignment (Fig. 4a–c) because there is not a standardized way to calculate fragment length, which leads to inconsistencies [36]. We refer to this (Fig. 4a) as a “curation” step, which is implemented in the cfDNAPro R package (Fig. 7a).

We found that analytical settings affect fragment lengths more significantly than the choice of library kits (Fig. 4d–o, Additional file 1: Figs. S4, S5, and S7–S9). Without a correct fragment length calculation, trimmed data still exhibit issues, particularly in the 150 bp range (i.e., read length): Tag_seq_HV aligned with Bwamem2 demonstrated thresholding problems, as indicated by black triangles in Fig. 4i. Different library kits show variations in different length ranges (Fig. 4p and q).

While adopting optimal processing parameters (i.e., “TrimBwamem2LengthPrior” in Table 2) and standardized feature extraction methods (Figs. 5b and 7a), fragment lengths from different library kits and individuals exhibited a relatively homogeneous distribution. PCA analysis further revealed a subtle clustering effect based on the library kits (Fig. 6a); in contrast, fragment motif is more significantly affected by the library kits than fragment lengths (Fig. 5c–k, Additional file 1: Figs. S11–S16). PCA analysis revealed an apparent kit-wise clustering effect, which strongly indicates the necessity of quality control and harmonization of motif quantification, especially when the training and testing data for machine learning models are derived from different protocols (Fig. 6b).

Our results on community healthy plasma data indicate the existence of batch effects across these studies. The experimental impacts on the results could not be eliminated by using the same standardized processing pipeline (Fig. 6d–e, Additional file 1: Figs. S22 and S24). The batch effects in published studies could be a combination of various factors. We analyzed several factors: study (datasets/author names), extraction kit, library kit, sequencer, data source (NRLAB/EGA/FinaleDB), gender, and age. Based on our analysis, study, extraction kit, and library kit factors are closely linked with each other; thus, in the PCA analysis, all of these factors present clustering effects; the sequencer factor might be confounded by study, extraction kit, and library kit, thus less informative. Data source, gender, and age did not show obvious batch effects.

We discussed data harmonization of the features extracted from different studies (Fig. 6f, Additional file 1: Figs. S32 and S33). The harmonization could preserve the ctDNA signals while attenuating batch effects. From a practical point of view, in studies combining various datasets, the “datasets” might be an appropriate variable to harmonize against because they represent the variations derived from any factors specific to individual studies. However, whether or not to adopt such harmonization should be subject to specific study designs.

To achieve standardized and reproducible quantification of cfDNA, we implemented the TAP pipeline for library-specific trimming and alignment. Considering the broad user community and comprehensive infrastructural supports for bioinformatics [38, 65], we developed the biological feature extraction and visualization as an R package called cfDNAPro. Both are available on GitHub. cfDNAPro has been serving the user community since 2021.

Within cfDNAPro, we developed various functions for multi-modal feature extraction, such as the *readBam()* function for reading bam files and curation, *readLength()* and *plotLength()* for length analysis, and *readMotif()* and *plotMotif()* for motif analysis. Moreover, by integrating an optional mutational annotation feature into the *readBam()* function, we introduce a comprehensive method for annotating fragments that overlap with a priori variant loci generated by external means. Our approach could ascertain if either one or both paired-end reads support the variant base (Fig. 7b). Recognizing fragments with inconsistencies gauges the noise associated with a given locus. Users can filter mutated cfDNA fragments based on their mutational categories, enabling them to derive trinucleotide mutation counts via *callTrinucleotide()* and visualize the substitution frequencies via *plotTrinucleotide()*. By integrating fragment-specific metrics, such as length and end context, with the fragment’s mutational status, our method sets a new standard for comprehensive cfDNA data analysis (Fig. 7d, f, g, and h). We also implemented *plotCNV()* as a modern way to visualize CNAs with gene annotation utility depending on *ggplot2* and *ggrepel* R packages [72, 73] which gives the flexibility to customize the plot using ggplot syntax (Fig. 7g). In addition, cfDNAPro includes essential functions frequently used in the research area, such as downsampling bam files and summarizing bam statistics. We plan to regularly add support for other analyses and visualizations, such as nucleosome position calling and coverage signature analysis of fragments. We anticipate that cfDNAPro and the data reported in this study will improve the efficiency and reproducibility of cfDNA fragmentomics analyses and lay a solid foundation for further methodological development for cancer detection in the study field. For example, when building multi-modal AI for cancer screening, these practices would be encouraged: (1) using a reproducible and correct trimming, alignment, and feature extraction pipeline. (2) Avoiding using biomarkers that are easily biased by experimental procedures. Robust features against various biases should be adopted. Feature harmonization might be considered when it fits in the study design. (3) Adopting machine learning models that are resilient against batch effects.

## Conclusions

This is the first systematic study comparing the fragmentomics results from different lab experimental and analytical approaches. Different library kits exhibited variations in sequencing data properties and fragmentomic feature profiles. The analytical approaches can affect fragment lengths, and the inherent properties of various library kits bias the motif profiles. This information is pivotal for building multi-modal AI models for cancer detection, especially when conducting multi-center studies and integrating data derived from various protocols. We proposed optimized solutions to those challenges and developed TAP for library-specific trimming and cfDNA-specific alignment to accelerate research and ensure robust data pre-processing. We also developed an open-access R package called “cfDNAPro,” which implements cfDNA-specific feature extraction methods, e.g., fragment length, motif, mutations, and copy number aberration. More importantly, it provides a unified framework for conducting multi-feature studies, unlocking the possibility of orchestrating multi-modal feature integration and uncovering innate relationships across biomarkers. The evaluation of experimental and analytical impacts, alongside collated healthy plasma datasets from various studies, the TAP, and the cfDNAPro package, are essential resources for advancing the understanding of cfDNA biological features. Our study accelerates the adoption of best practices in reproducible science and provides a roadmap for future cfDNA multi-modal features integration research.

## Methods

### Sample collection, cfDNA extraction, and library preparation

Plasma samples from 10 healthy donors were obtained from BioIVT stored at − 80 °C until DNA extraction. The blood processing protocol is provided by BioIVT: (A) blood is collected into EDTA tubes. (B) The whole blood collected undergoes two centrifugations: (1) 1600 g for 10 min to separate the plasma from the whole blood within 1 h of collection, then immediately (2) taking the plasma from (1), run a 2nd centrifugation at 8000 g for 10 min. (C) Collect the supernatant from (2) and transfer (without disturbing the pellet) to new 2-mL tubes. Discard the pellet. Freeze to − 20 °C. Shipped to the lab with dry ice. Stored in the lab at − 80 °C.

cfDNA was purified from 3.8 to 4.1 mL of plasma using the QIAsymphony DSP Circulating DNA Kit (QIAGEN). To assess extraction efficiency, a non-human spike-in control (an amplicon of 170 bp derived from *Xenopus tropicalis*) was added to the lysis buffer during cell-free DNA extraction, following the method described by previous studies [53, 74]. The extracted cell-free DNA was quantified by digital PCR and then stored at − 80 °C until further use. cfDNA quantification by dPCR of human RPP30 locus and also by Agilent cfDNA TapeStation is shown in Additional file 2: Table S6.

Around 750–1000 haploid genome copies (around 3.3 ng) of plasma DNA were used for library preparation. The libraries were prepared following manufacturer guidelines. The library kits used in this study include XTHS and XTHS2 from Agilent Technologies; PlasmaSeq, Tag_seq, and Tag_seq_HV from Takara Bio; EM_seq and NEBNext_Ultra_II from New England Biolabs; Watchmaker from Twist Bioscience and Watchmaker Genomics; and KAPA_HyperPrep from Roche (Fig. 2). The number of amplification cycles varied according to the manufacturer’s recommendation, as detailed in Table 1. Donor a and c in XTHS and donor c and j in EM_seq, NEBNext_Ultra_II, and KAPA_HyperPrep were excluded from the analyses due to a lack of DNA materials (Additional file 2: Table S1).

### Library-specific adapter trimming

Due to the differences in the amplicon structures of various libraries, we adopted a library-specific trimming strategy: first, a single Unique Molecular Identifier (UMI) and single sample barcode: XTHS. Adapters were trimmed using Trim Galore! [75] (Fig. 2a).

Second, dual UMI and dual indices: XTHS2 (Fig. 2b), Tag_seq (Fig. 2d), Watchmaker (Fig. 2g), and KAPA_HyperPrep (Fig. 2h). Both kits have “dark bases” (or referred to as “stem sequences” or “skipped bases” by kit manufacturers) between UMI and cfDNA fragments. XTHS2 was trimmed using AGeNT [76] software supplied by Agilent Technologies. Tag_seq was trimmed using an in-house tool “tag-trim,” which identifies the stem sequence from 3′ end of sequences and removes all bases after. Watchmaker and KAPA_HyperPrep are trimmed by directly removing a specific length of bases (i.e., UMI + “skipped bases”). Third, dual UMI and dual samples barcodes but without any intervening sequences between the i5 UMI and cfDNA fragments: Tag_seq_HV (Fig. 2e). Trimming was conducted using Trimmomatic [77] software according to the library kit user manual. Moreover, when there is no UMI but with dual sample barcodes: PlasmaSeq, EM_seq, and NEBNext Ultra II, adapters were trimmed using Trim Galore! [75] (Fig. 2c, f, and i).

### Sequencing data alignment

Libraries were sequenced using Illumina NovaSeq 6000 (PE150 bp). We utilized Bowtie2 (version 2.5.1) and Bwamem 2 (version 2.2.1) to align the PE sequencing data. For Bowtie 2, the default settings, “–local” and “–local –soft-clipped-unmapped-tlen” options were used in various iterations. For bwamem2, the default setting and “-I 167,1000” were used in different analytical routes in Table 2. Trimming and library-specific alignment steps are implemented as the TAP pipeline available on GitHub; the pipeline utilizes singularity containers to meet high data analysis reproducibility and scalability standards for users. A schematic overview of the TAP is shown in Additional file 1: Fig. S18. Version number of software and tools integrated into TAP is available in Additional file 2: Table S2. Resulting BAM files were downsampled to 1 × to match the lowest coverage of the data.

### Handling of healthy plasma whole-genome sequencing data from studies

For data from NRLAB and EGA: Mouliere et al. [13], Santonja et al. [53], Ulz et al. [20], Zviran et al. [59], Peneder et al. [15]. The sequencing data (i.e., FASTQ) files were trimmed and aligned using TAP pipeline with optimal parameter settings (i.e., “TrimBwamem2LengthPrior”), BAM files were downsampled to 1 × to match the lowest coverage of the data collated.

For data from FinaleDB: Jiang et al. [17] and Cristiano et al. [14]. FinaleDB processed the sequencing data with a pipeline reported by Zheng et al. [60]. The alignment coordinates of fragments stored in tab-separated values (TSV) were provided for each sample. We downloaded the TSV files from FinaleDB and converted to bam files and downsampled to 1x. Fragment length between 100 and 220 bp were extracted using *readBam()* and *callLength()* functions in cfDNAPro. s3 motifs were calculated using *readBam()* and *callMotif()* functions in cfDNAPro.

### cfDNAPro implementation

cfDNAPro is built using R. It is available via GitHub, Bioconductor, and Anaconda repositories (see Code availability). The package was designed and tested using R version 4.1.0 and is compatible with R version 4.1.0 (or later) on multiple operating systems (Windows/macOS/Linux). R was chosen due to its open-source nature, general preference, and availability of infrastructural data structure (e.g., GRanges, GAlignmentPairs) for genomic data analysis within the bioinformatics community.

The architecture of cfDNAPro could be stratified into three categories: the first section is responsible for data curation (i.e., ensuring the correct fragment length calculation) and contains one primary function: readBam(). It will first check if the input Bam file contains paired-end reads, then import them into a GAlignmentPairs object and transform them into fragments (i.e., from paired reads to fragments). This gets stored in a GRanges object for optimum storage efficiency. Data quality control and alignment curation (Fig. 4) are implemented in this step. Furthermore, annotations are added to the GRanges object as meta columns, e.g., fragment length and fragment start motif, to facilitate fragment selection based on the meta information of each fragment. The *readBam()* function also provides an optional feature for annotating user-provided mutation loci with fragment-level specifics. This results in additional meta columns encompassing details about the count of fragments supporting the reference allele, the number of fragments favoring the alternative allele, and the determination of whether paired-end reads encompass the mutation site. A priori mutations are read from tab-separated format lists, obtained from matched tumor samples or alternative sources. De novo mutation lists can be generated using the *pileupMismatches()* function, which leverages *Rsamtools::pileup()*, and then used as a mutation file in the *readBam()* function.

The second section consists of feature extraction. cfDNAPro offers utilities to extract various biological features from the annotated GRanges object exported by the *readBam()* function. The features are stored in a Tibble object [78], e.g., fragment length, i.e., *callLength()*, and fragment motif, i.e., *callMotif()* and *callTrinucleotide()*. Copy number extraction method *callCNV()* depends on the QDNAseq package and stores results in QDNAseqCopyNumbers object [79]. In addition, *summariseBam()* is also available for calculating descriptive statistics such as the number of reads, number of mapped reads, number of reads mapped to mitochondrial sequences, and the overall coverage of a bam file.

The final section is responsible for the feature visualization. Various plots are available, such as the fragment length distribution, plotted by function *plotLength()*, the fragment end motif frequencies, as plotted by *plotMotif()*, frequency of single nucleotide mutation classified by their trinucleotide context *callTrinucleotide()*, and copy number plots, plotted by *plotCNV()*. All visualization functions depend on the *ggplot2* R package because *ggplot2* offers state-of-the-art utilities and mature ecosystems [72]. This means the resulting visualization object could be modified further by users within *ggplot2* ecosystem.

### Quality control and curation of alignments

We implemented the two essential steps, i.e., “QC” and “curation” (Fig. 7a). Specifically, in QC steps: (1) reads mapping qualities less than 30 were discarded; (2) reads must be paired. Of note, by default, cfDNAPro does not impose filtration by “proper pair”; (3) no duplicate; (4) no secondary alignment; (5) no supplementary alignment; (6) no unmapped reads.

Regarding the “proper pair” mentioned in QC criteria (2) above, although filtering by “proper pair” is a common quality control step in the next-generation sequencing data analysis, we do not recommend the same filtration in cfDNA sequencing data: this “proper pair” is assigned to each read pair by aligners. For example, the bwa-mem algorithm assumes fragment length as a normal distribution and infers the mean and standard deviation by default: “The maximum distance x for a pair considered properly paired (SAM flag 0 × 2) is inferred by the software, and for mapping Illumina short-insert reads to the human genome, x is about 6–7 sigma away from the mean fragment length.” While this assumption works for most of the traditional tissue sequencing data, it does not fit the scope of cfDNA fragmentomic research, as cfDNA lengths are not normally distributed (e.g., the di- and tri-nucleosome peak). Thus, filtering by “proper pair” will lead to the potential loss of fragments in the di-nucleotide region (Additional file 1: Fig. S3).

Following the QC step, cfDNAPro curates the coordinates of the fragments, which ensures the correct definition of a fragment (Fig. 4a and b): (1) remove read pair seqname discordance; (2) remove read pair without strand info; (3) only keep inwardly directed read pairs; (4) the start of the forward read as the new start position; (5) end of the reverse strand read as the new end position; (6) remove out-of-bound fragments.

### Fragment length and motif analysis

Fragment lengths were extracted using the cfDNAPro package (version 1.7.2) described above with the following code: *result* <—*cfDNAPro::readBam(bamfile, genome_label* = *“hg38”, curate_start_and_end* = *TRUE) |*> *cfDNAPro::callLength(genome_label* = *“hg38”)*.

Although cfDNAPro supports eight types of motifs (Fig. 5a and b), all fragment end motifs were “s3” motifs, i.e., the first three bases of each fragment. The code used was *result* <—*cfDNAPro::readBam(bamfile, genome_label* = *“hg38”, curate_start_and_end* = *TRUE) |*> *cfDNAPro::callMotif(genome_label* = *“hg38”, motif_type* = *“s”, motif_length* = *3)*.

Only fragments between 50 and 450 bp were kept for downstream analyses. For results without alignment curation (i.e., analyses with problematic fragment length calculation), the “curate_start_and_end” parameter was set to FALSE.

### Statistical tests

The statistical tests were done using R (version 4.3.2) [38]. The metrics between different groups (https://zenodo.org/records/15221979) in Fig. 3 were compared using the *stat_compare_means()* function implemented in the *ggpubr* package (version 0.6.0) [80], which depends on the *wilcox.test* (i.e., Wilcoxon signed rank test, two-sided) utility in the *stats* package (version 4.3.2) [38]. The PCA analysis was performed using the *prcomp()* function in the *stats* package (version 4.3.2) [38]. Feature harmonization was performed using *Combat_seq()* function [62, 63] in *sva* R package (version 3.50.0) [61]. PCA results were visualized with the *factoextra* package (version 1.0.7) [81]. The group mean points were shown, and ellipses surrounding each cluster was 95% confidence area around group mean points.

## Supplementary Information

Additional file 1. A file containing additional Figs. S1–S33

Additional file 2. A file containing additional Tables S1–S7

## Data Availability

cfDNAPro is an open-access Bioconductor/R package released under the GPL-3 Open Source license, it supports Windows, Linux and macOS systems. It requires R version ≥ 4.1.0. The latest version of cfDNAPro can be obtained from https://github.com/nrlab-CRUK/cfDNAPro [82], its documentation can be accessed via https://cfdnapro.readthedocs.io/en/latest/index.html [83]. TAP pipeline is an Nextflow pipeline and it supports Linux system. Code and documentation are available via :https://github.com/nrlab-CRUK/TAP [84]; The exact versions of cfDNAPro, TAP used for this paper are available via Zenodo (https://doi.org/10.5281/zenodo.15132270 [85] and https://doi.org/10.5281/zenodo.14779585 [86]). The cfDNAPro documentation files are also available from Zenodo (https://doi.org/10.5281/zenodo.15221979 [87]). The datasets generated and/or analysed during the current study are available in the European Genome-phenome Archive (EGA) repository under accession number EGAS00001008051 [88, 13, 89]), Santonja et al [53] (EGAD00001008589 [90] and EGAD00001006293 [91]), Ulz et al [20] (EGAD00001005343 [92]), Zviran et al [59] (EGAS00001004406 [93]), Peneder et al [15] (EGAS00001005127 [94]). There are also two datasets available via FinaleDB (http://finaledb.research.cchmc.org): Jiang et al [17] and Cristiano et al [14]. The remaining data are available within the article, additional files, or available from the authors upon request.
