# Supplementary material for: A standardized framework for robust fragmentomic feature extraction from cell-free DNA sequencing data
Source: Genome Biol. 2025 May 23;26:141. doi: 10.1186/s13059-025-03607-5 (PMC12100915; doi:10.1186/s13059-025-03607-5)
Supplement: Supplementary file 1 — Additional file 1. A file containing additional Figs. S1–S33 [file 13059_2025_3607_MOESM1_ESM.docx]

# Additional File 1

**Wang et al**

**The file includes:**

**Additional Fig S1**-**S33**

## Additional figures


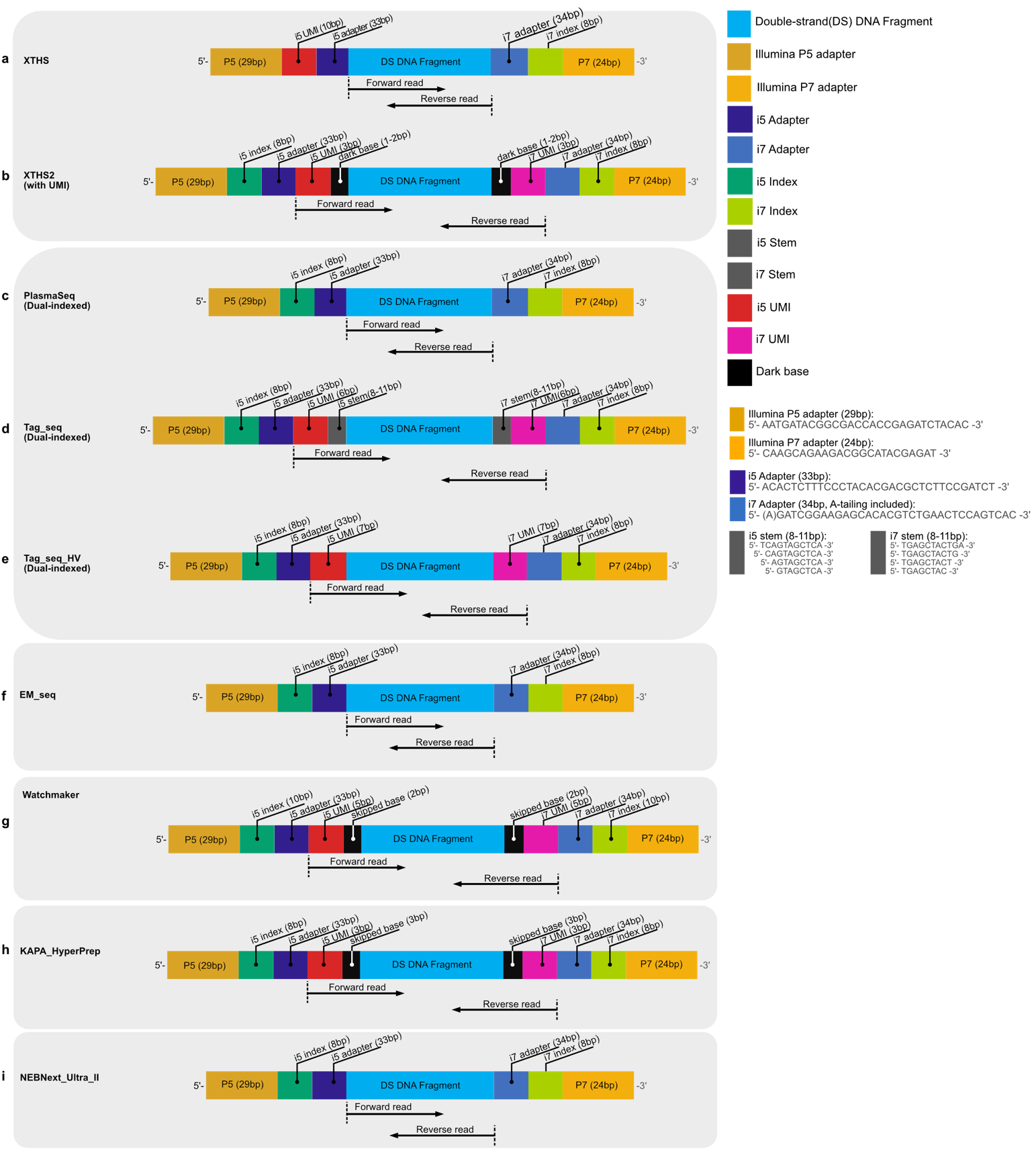


[**Fig S1**](#sfigu_amplicon) **Amplicon structures generated by library kits used in the study**. **a-g** Amplicon structures of various library kits. **h** Nucleotide sequences of different elements.

**
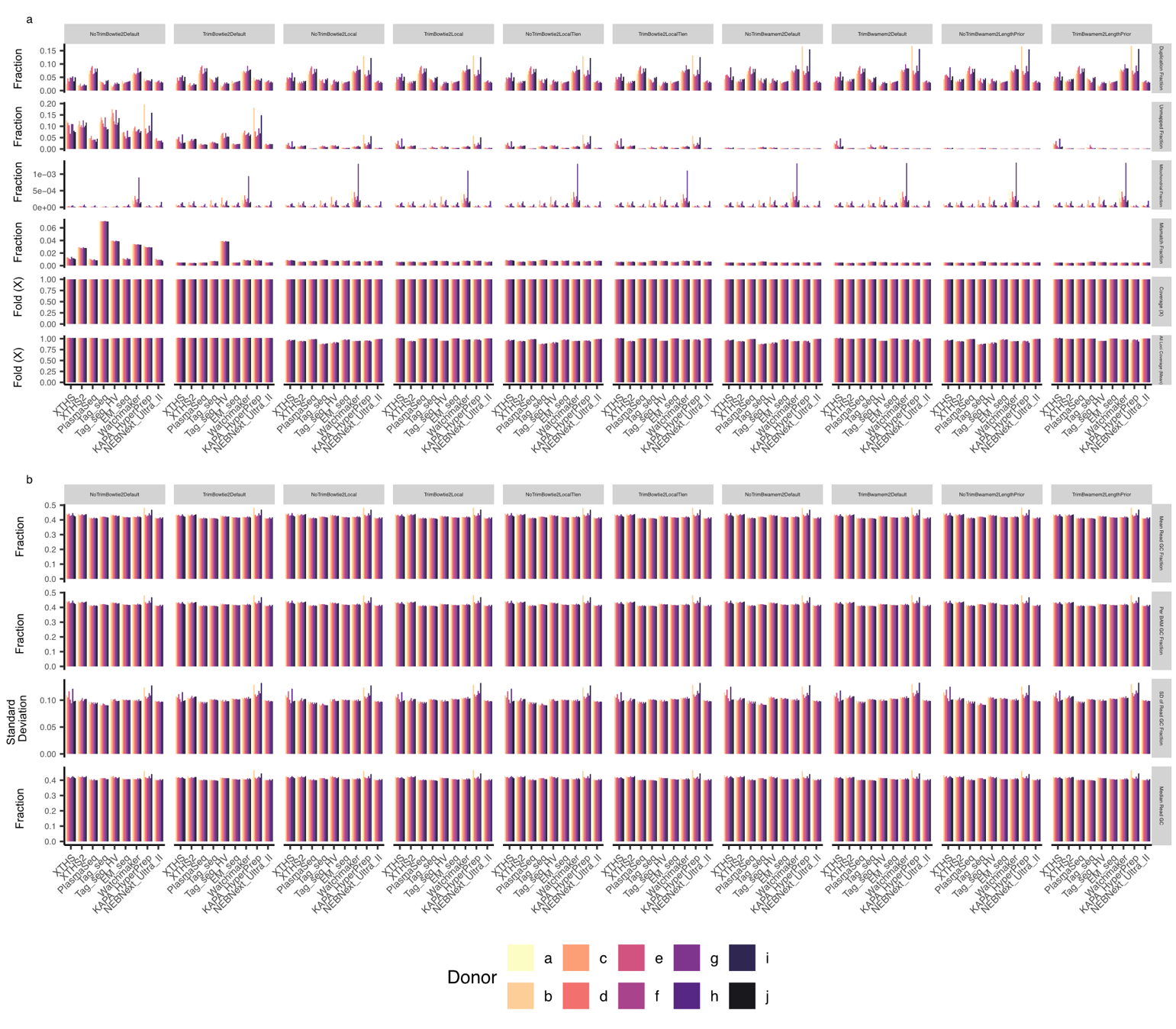
**

**Fig S2 Sequencing data descriptive statistics of all bam files generated in the study.** **a** Duplication fraction, Unmapped fraction, Mitochondrial fraction, Mismatch fraction, Coverage (after downsampling), and mean coverage across all genomic loci (after downsampling). Of note, the duplication rate can be affected by various factors, such as “PCR duplicates” and “sequencing duplicates,” can be affected by the PCR cycles specific to in-house lab protocols, the sequencer settings, and the downsampling method, etc; Different library kits generate highly variable fractions of unmapped reads. This raises special concerns for projects focusing on non-human reads (e.g., microbiome) from sequencing data. **b** GC content related metrics: mean GC% per read, GC% per bam file, Standard deviation of GC% per read, and median GC% per read.


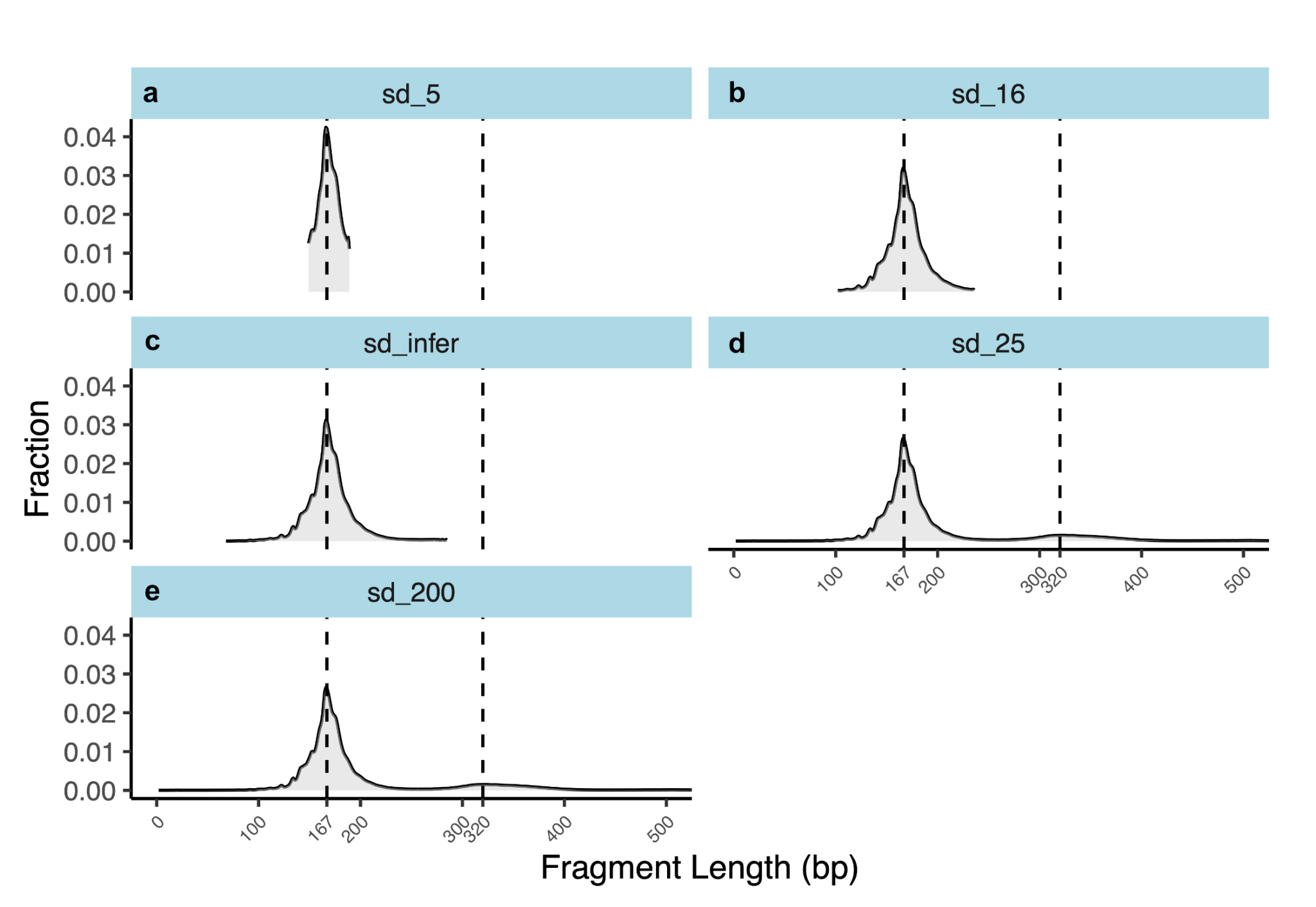


[**Fig S3**](#sfigu_aligner) **Impact of normal distribution assumptions on the fragment length distribution.** Reads were filtered by “proper pair” before calculating fragment length distributions. **a, b, d,** and **e**, During bwa mem2 alignment, through “-I” parameter, the mean of normal distribution parameters were set manually to 167 and sd to 5, 16, 25 and 200 respectively. **c** Leave the “-I” as the default setting (i.e., the aligner will infer mean and sd based on the input data).


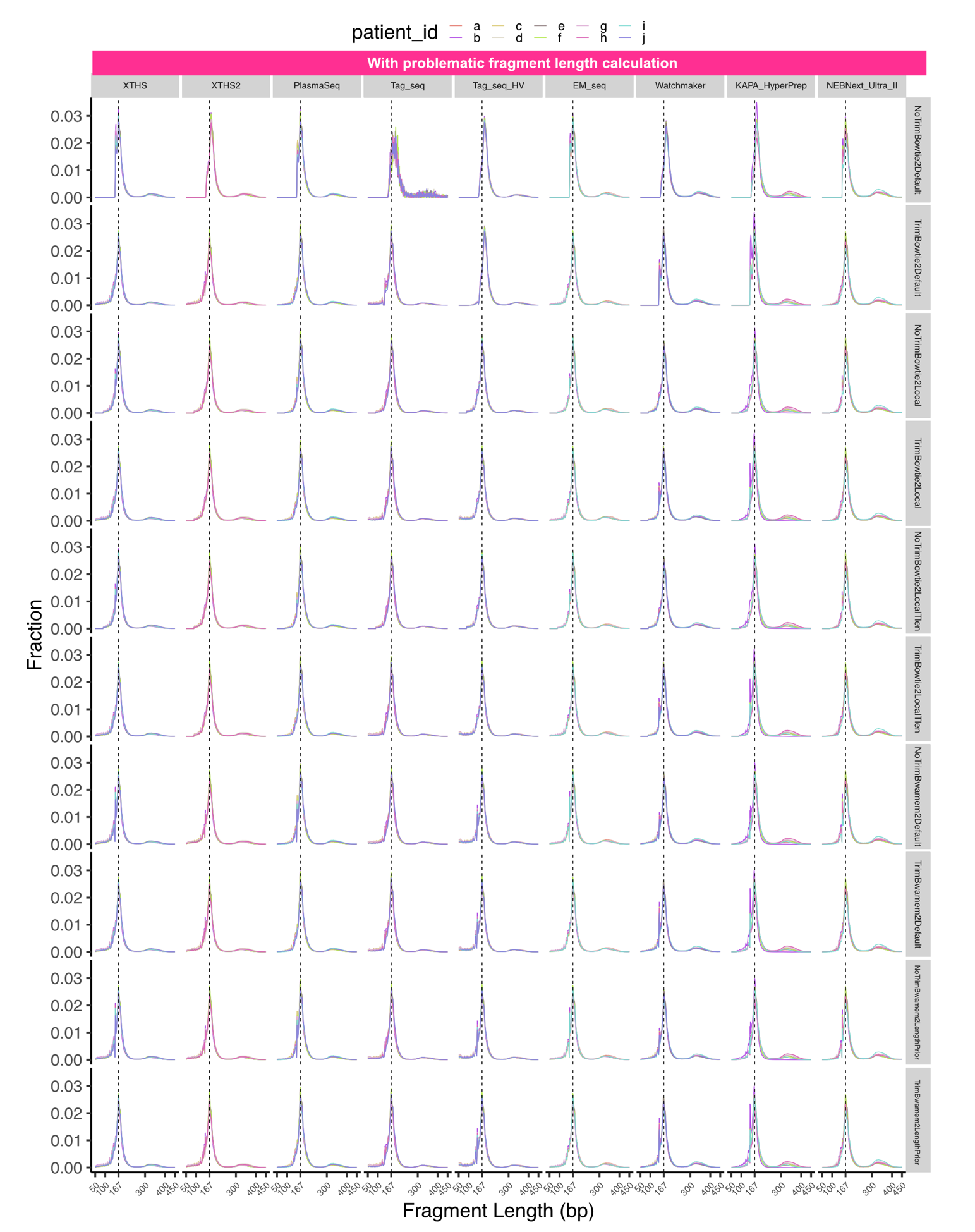


[**Fig S4**](#sfigu_lengths_nocuration) **Fragment length distribution (With problematic fragment length calculation)**. Data from all individuals, all libraries and all processing parameters.


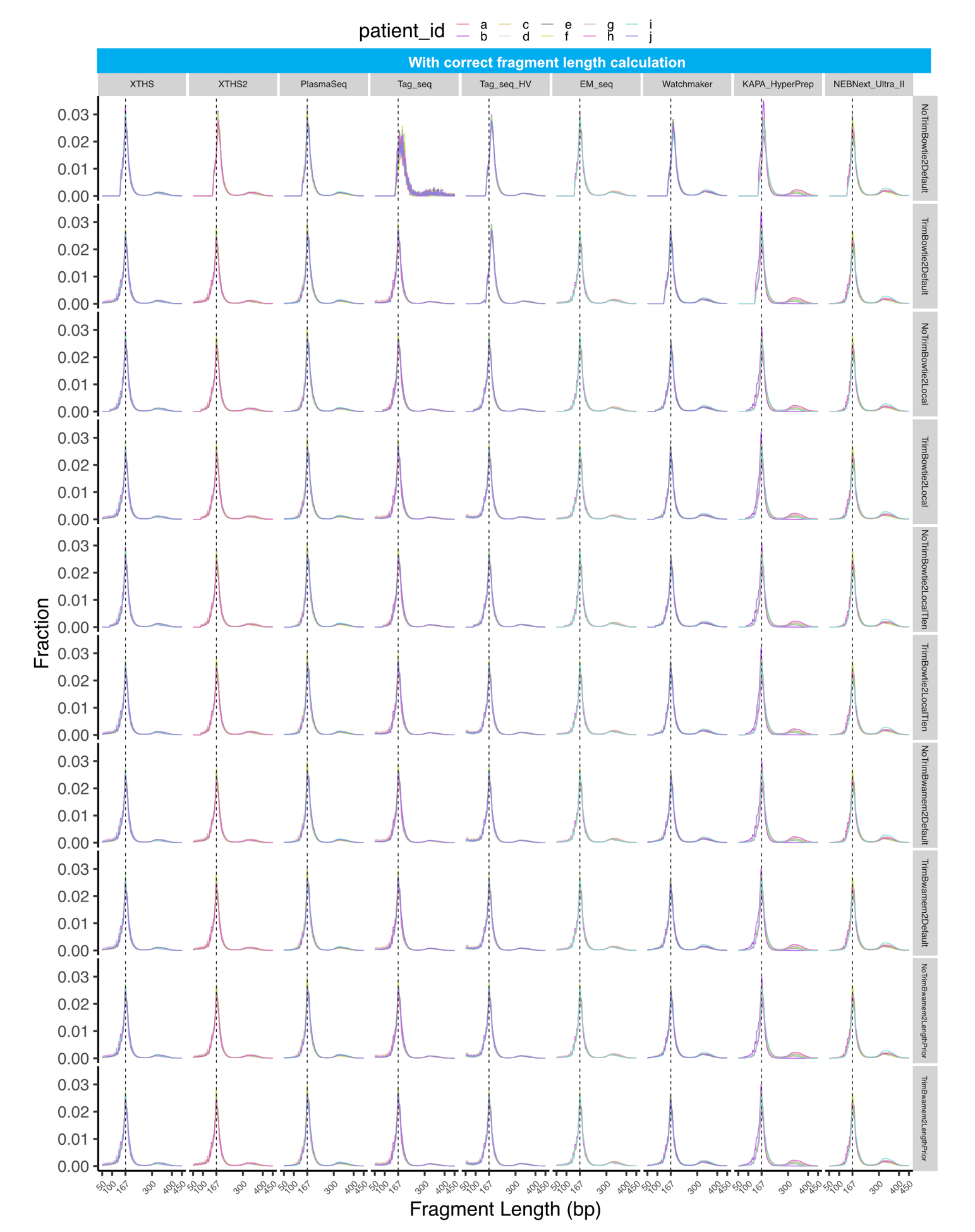


[**Fig S5**](#sfigu_lengths_curation) **Fragment length distribution (With correct fragment length calculation).** Data from all individuals, all libraries and all processing parameters.


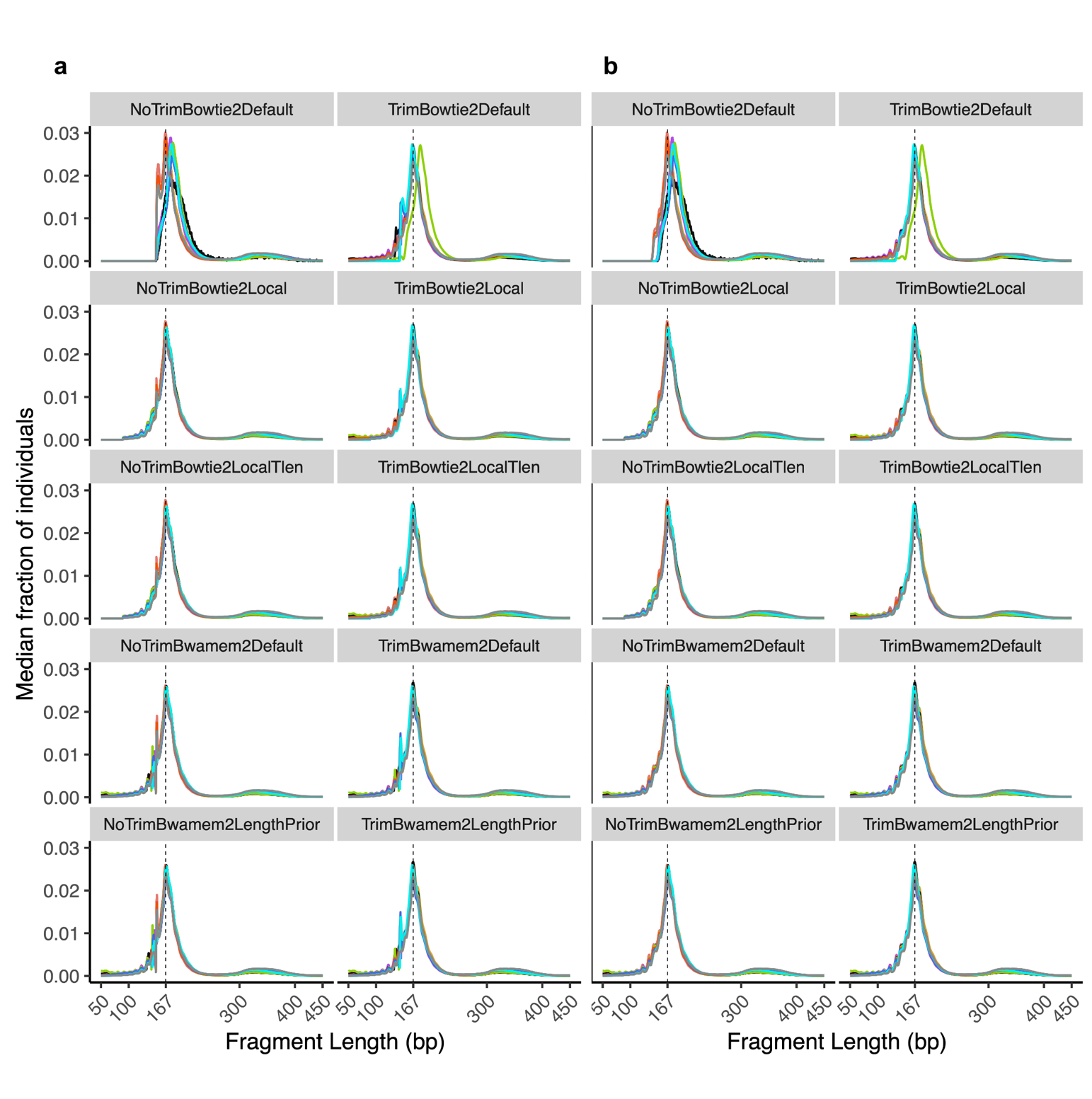


[**Fig S6**](#sfigu_lengths_facet_by_settings) **Median fragment length distribution of all individuals**. Facets are all analytical settings.

**a** With problematic fragment length calculation. **b** With correct fragment length calculation.


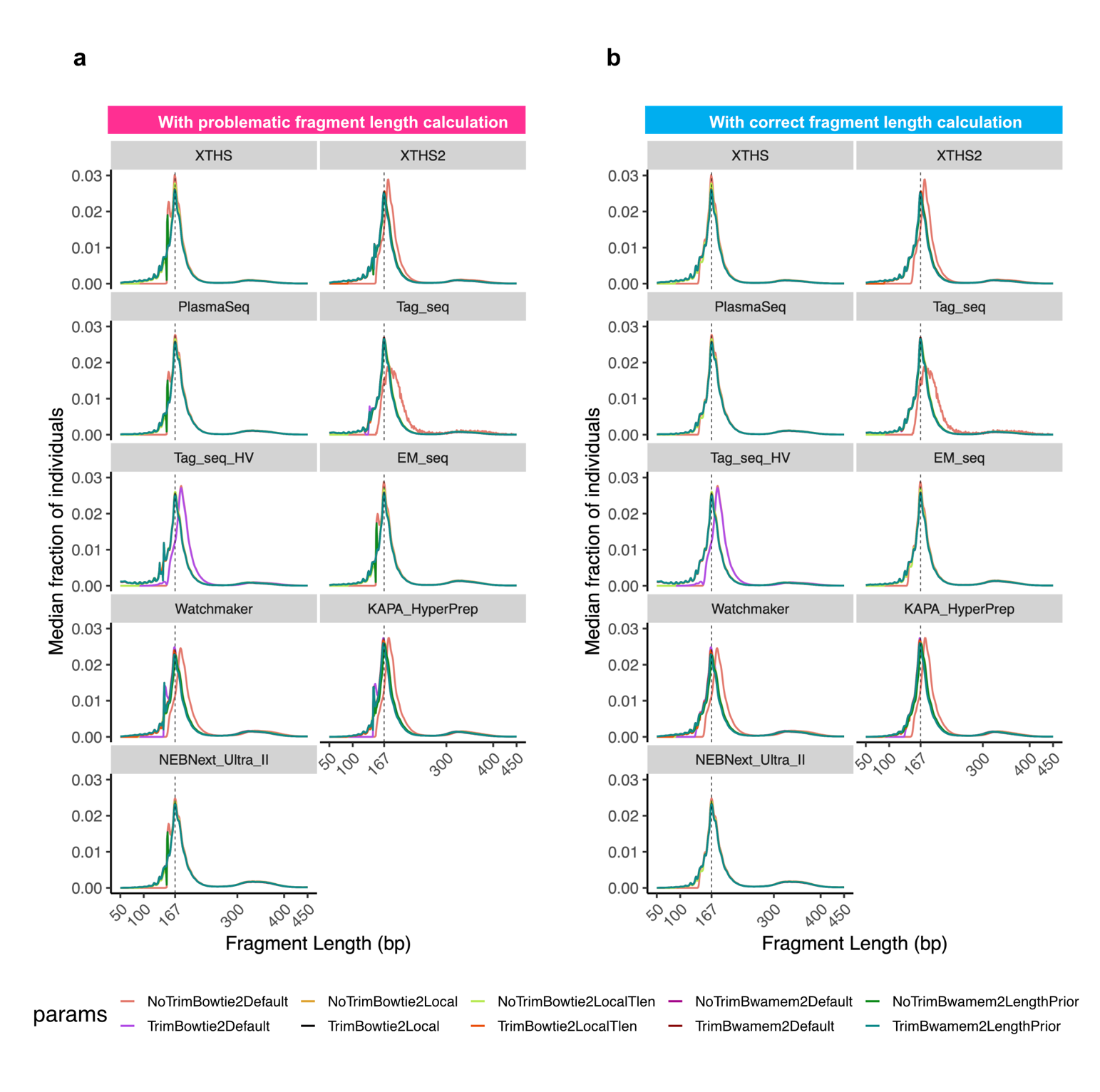


[**Fig S7**](#sfigu_lengths_facet_by_library) **Median fragment length distribution of all individuals**. Facets are all libraries, and lines in each panel are processing parameters.


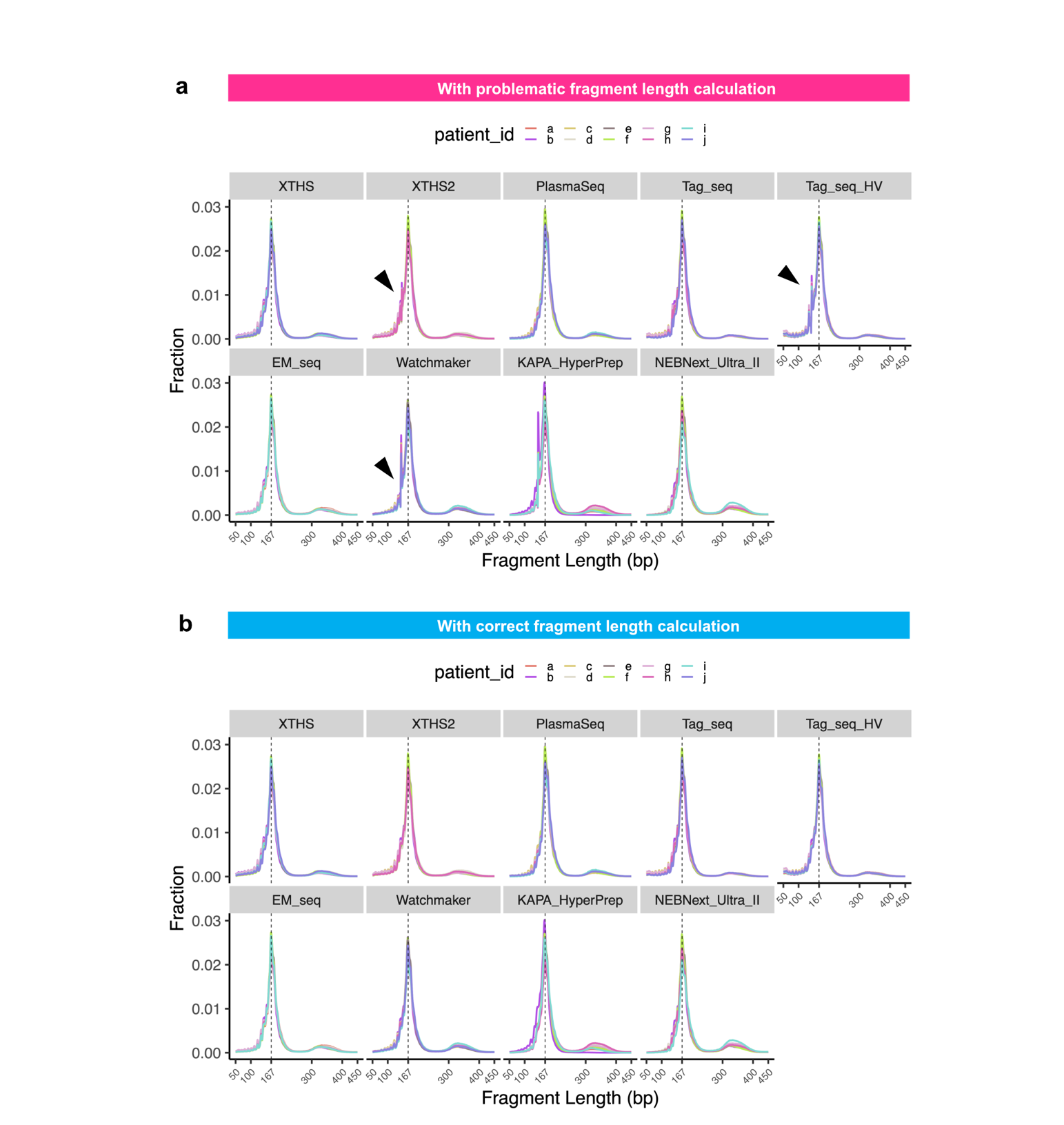


[**Fig S8**](#sfigu_lengths_facet_by_libs) Fragment length distribution of individuals, facets are all libraries, and lines in each panel are healthy donors. All samples were processed using the optimised parameter: TrimBwamem2LengthPrior.


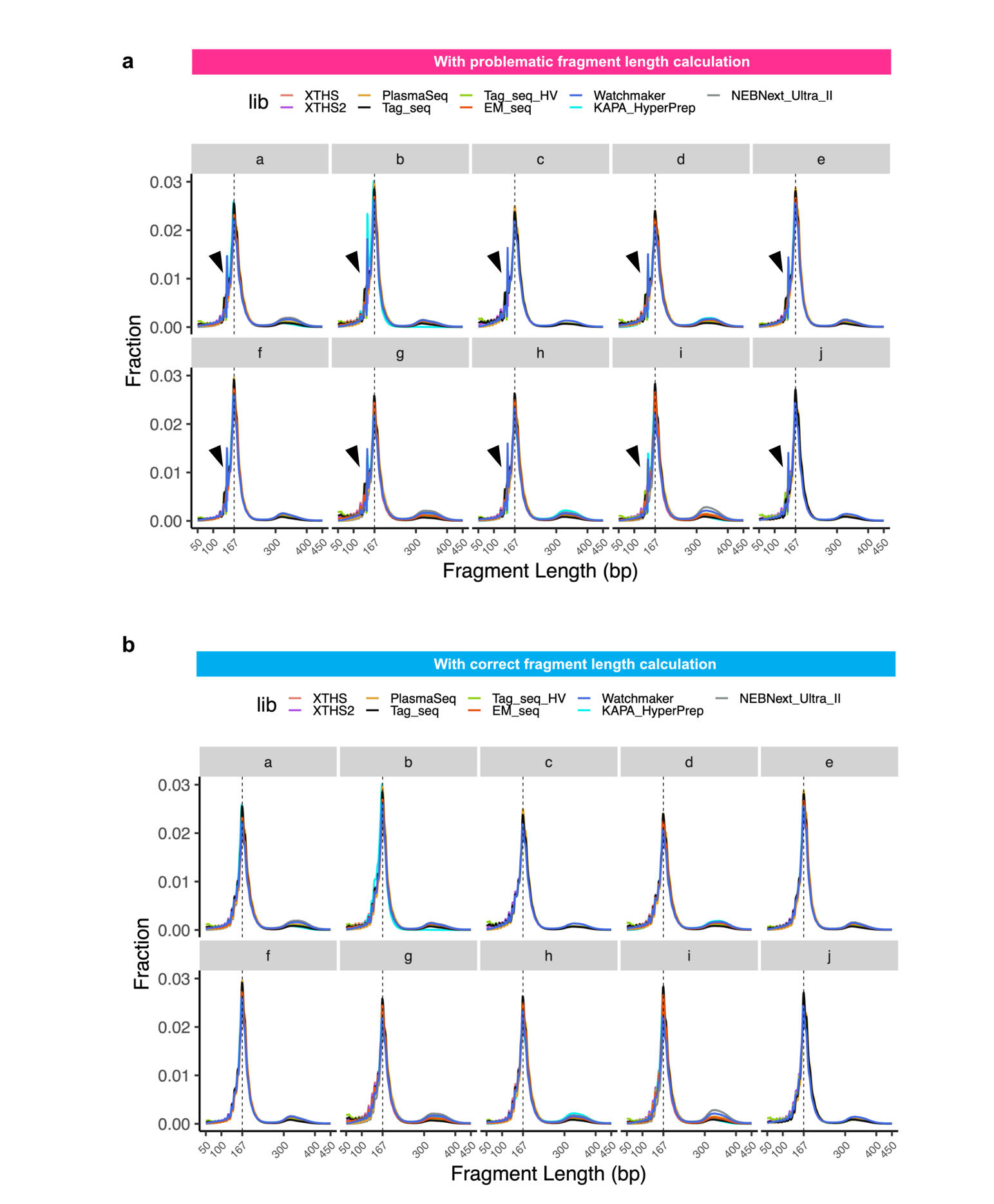


[**Fig S9**](#sfigu_lengths_facet_by_donors) **Fragment length distribution of individuals**. Facets are all healthy donors and lines in each panel are library kits. All samples were processed using the optimised parameter: TrimBwamem2LengthPrior.


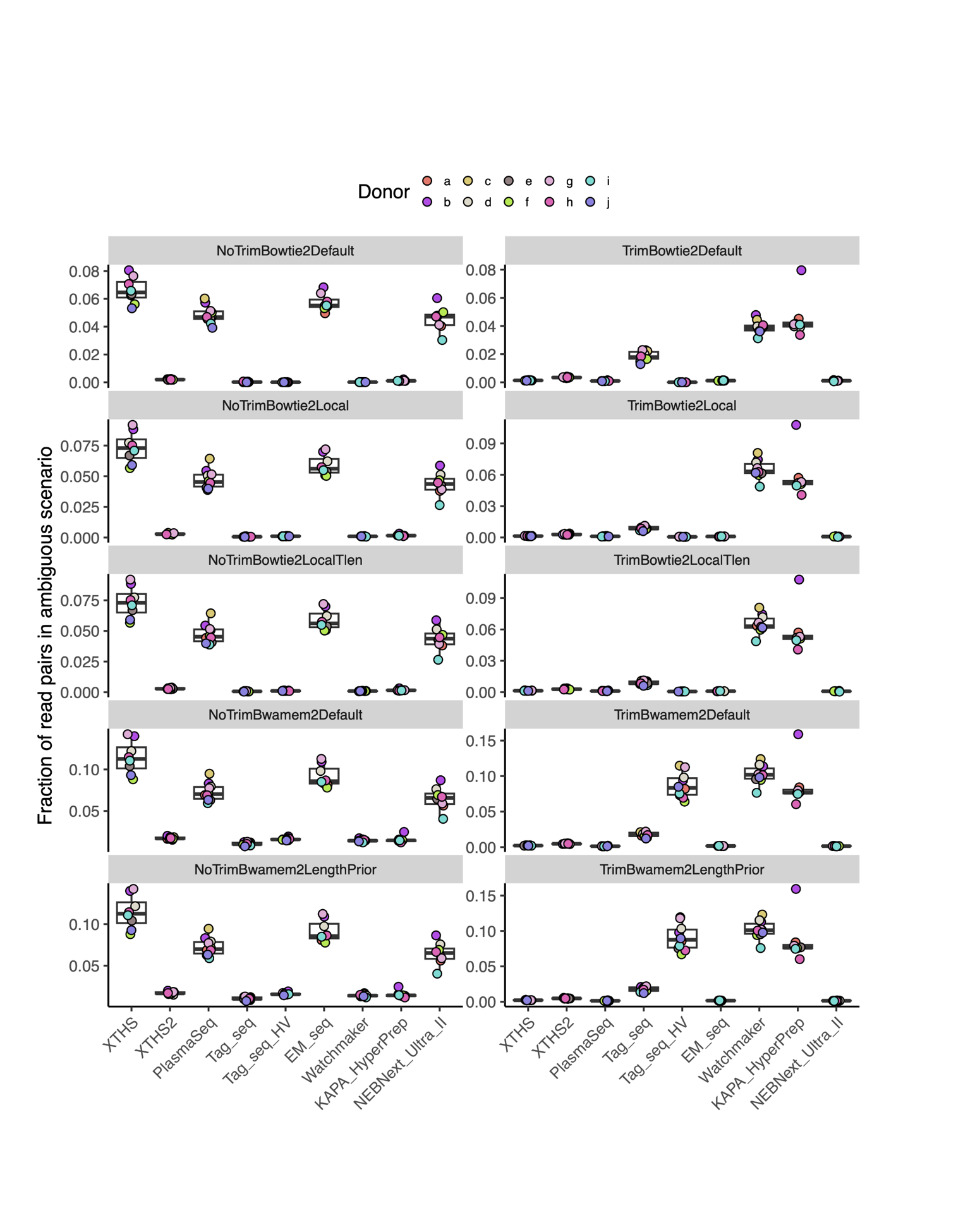


[**Fig S10**](#sfigu_senarios) **Quantification of ambiguous read-pairs in bam files**. The definition of each processing parameter is shown in [**Table 2**](#tab_trim_align_params).


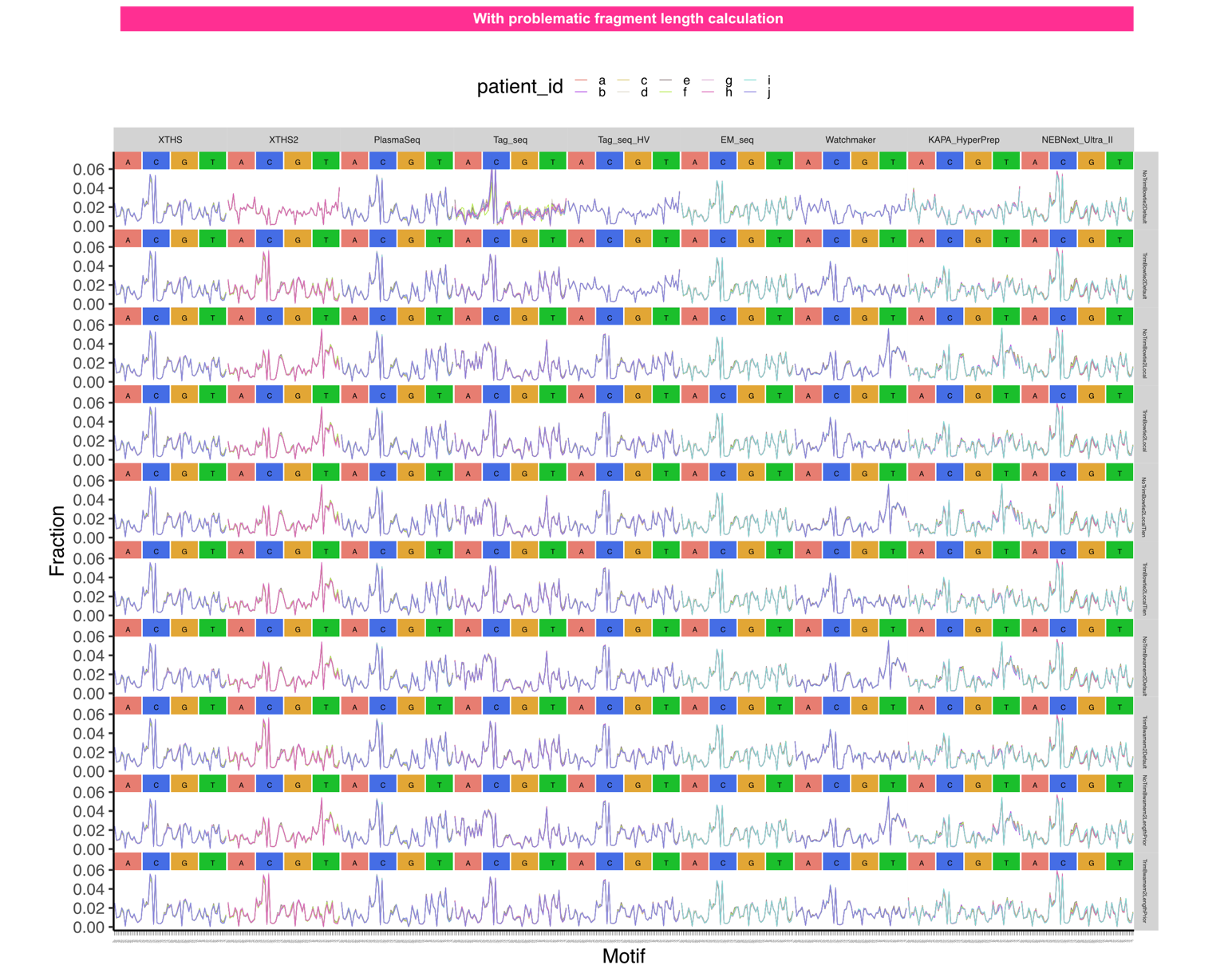


[**Fig S11**](#sfigu_motif_nocuration) **Motif distribution (without curation)**. Panels are libraries and parameters, and lines in each panel are healthy donors.


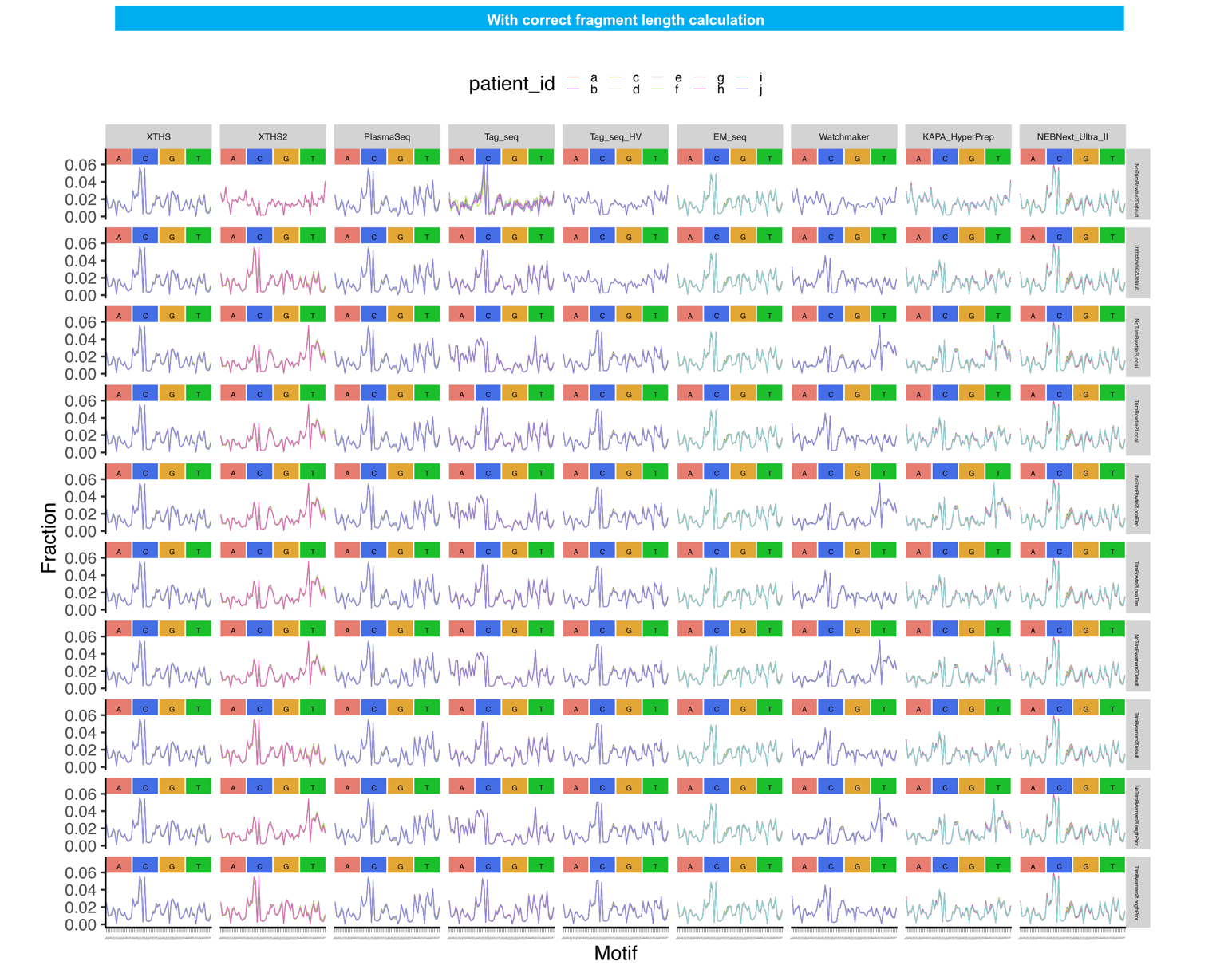


[**Fig S12**](#sfigu_motif_curation) **Motif distribution (with curation) of individuals**. Facets by libraries and parameters, and lines in each panel are healthy donors.


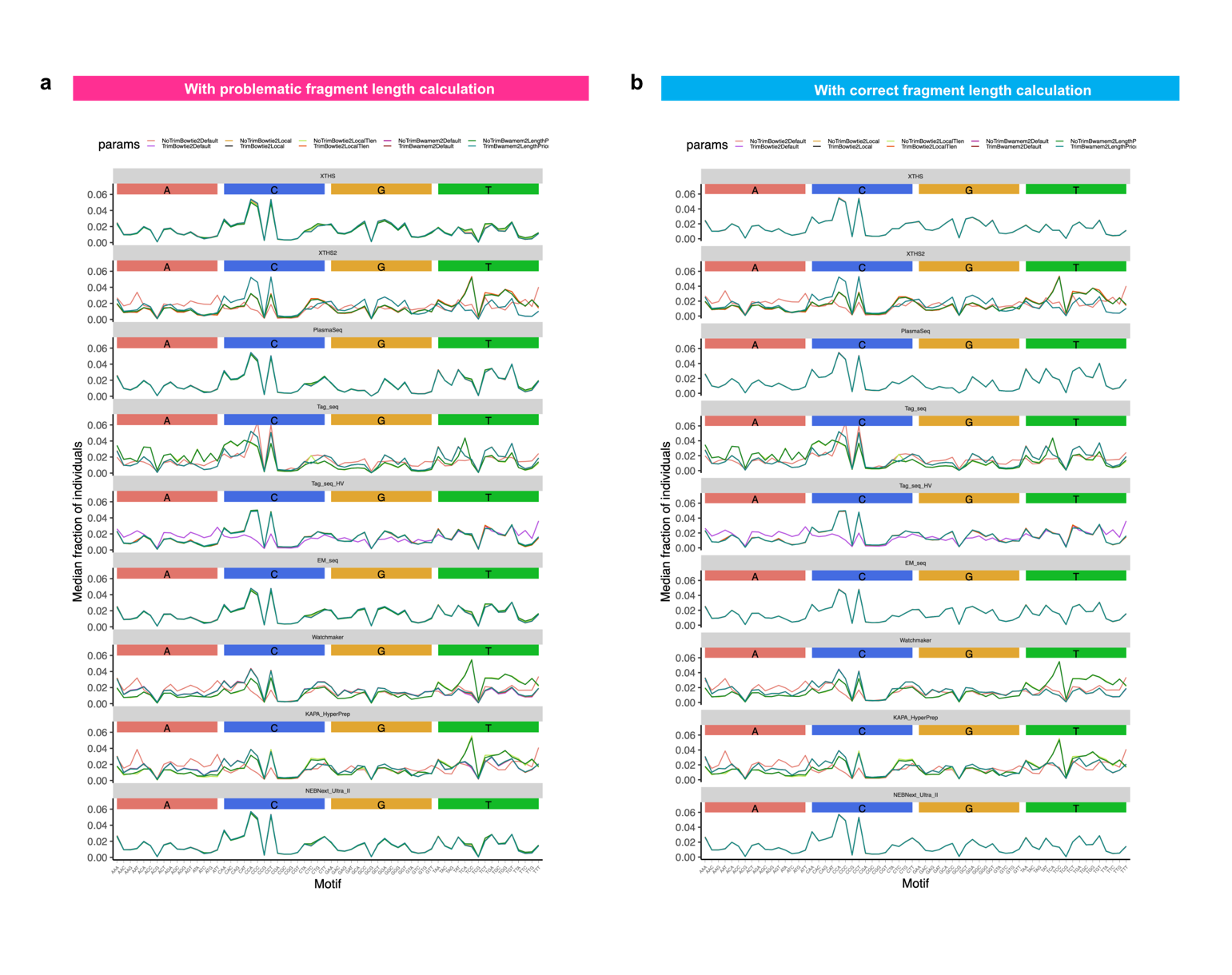


[**Fig S13**](#sfigu_motif_facet_by_lib) **Median motif distribution.** Facets by libraries, and lines in each panel are median s3 motif distribution across all healthy donors of each analysis parameter.


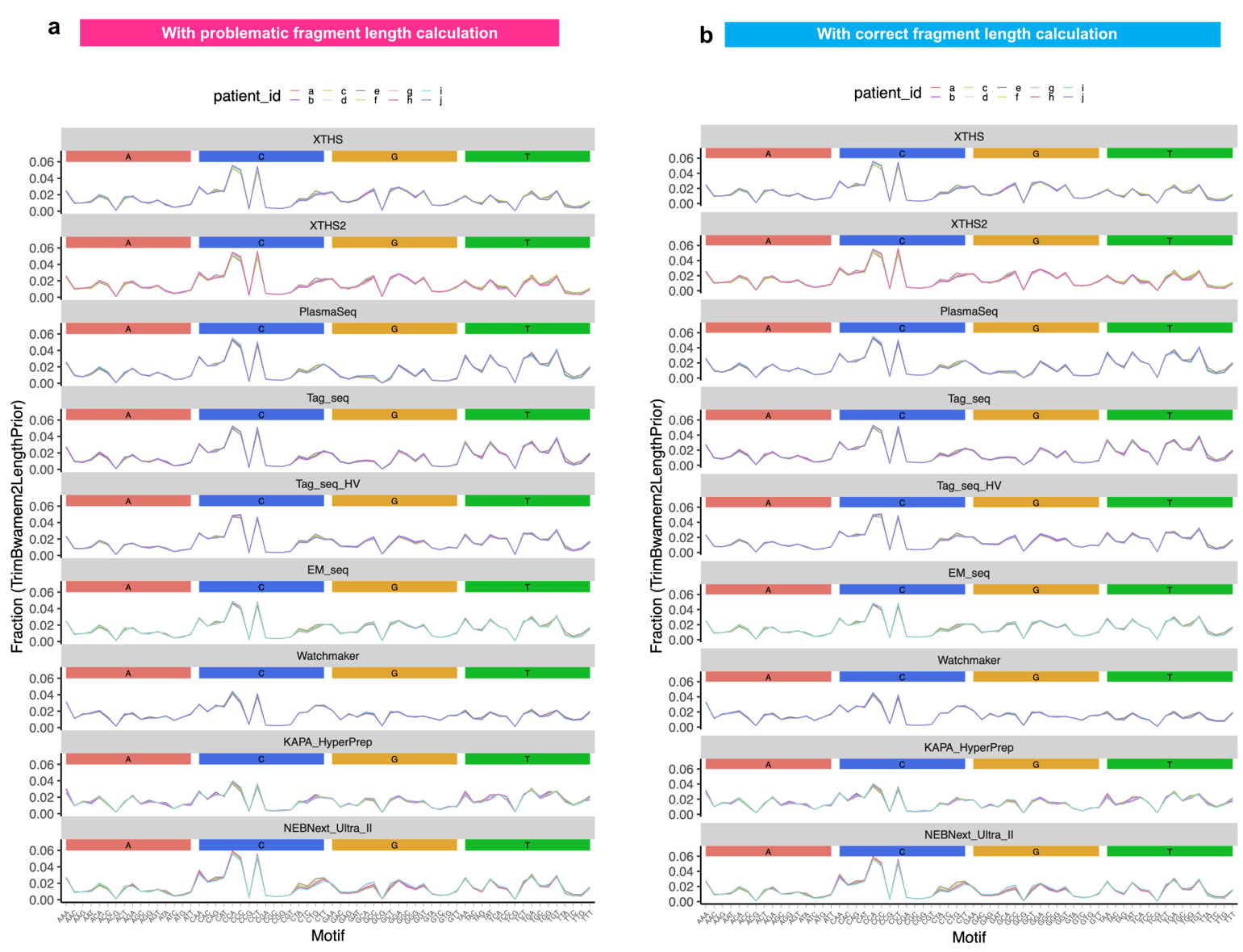


[**Fig S14**](#sfigu_motif_facet_by_library) **Motif distribution**. Panels are libraries, and lines in each panel are the median s3 motif distribution of each healthy donor. **a** With problematic fragment length calculation step. **b** With correct fragment length calculation.


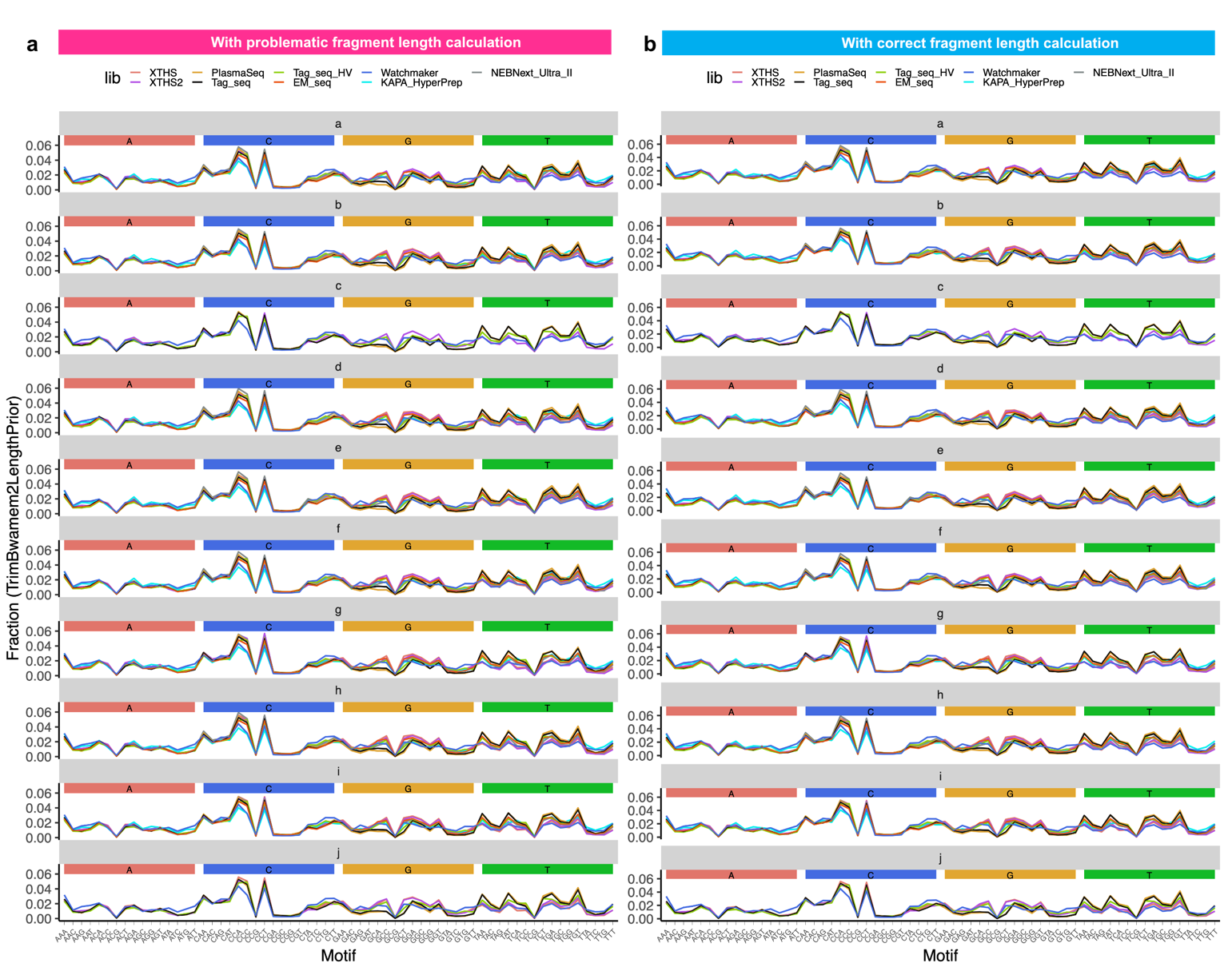


[**Fig S15**](#sfigu_motif_facet_by_donors) **Motif distribution**. Panels are different healthy donors, and lines in each panel are motif frequency from each library kit using the optimal trimming-alignment parameter (i.e., “TrimBwamem2LengthPrior”). **a** With problematic fragment length calculation. **b** With correct fragment length calculation.


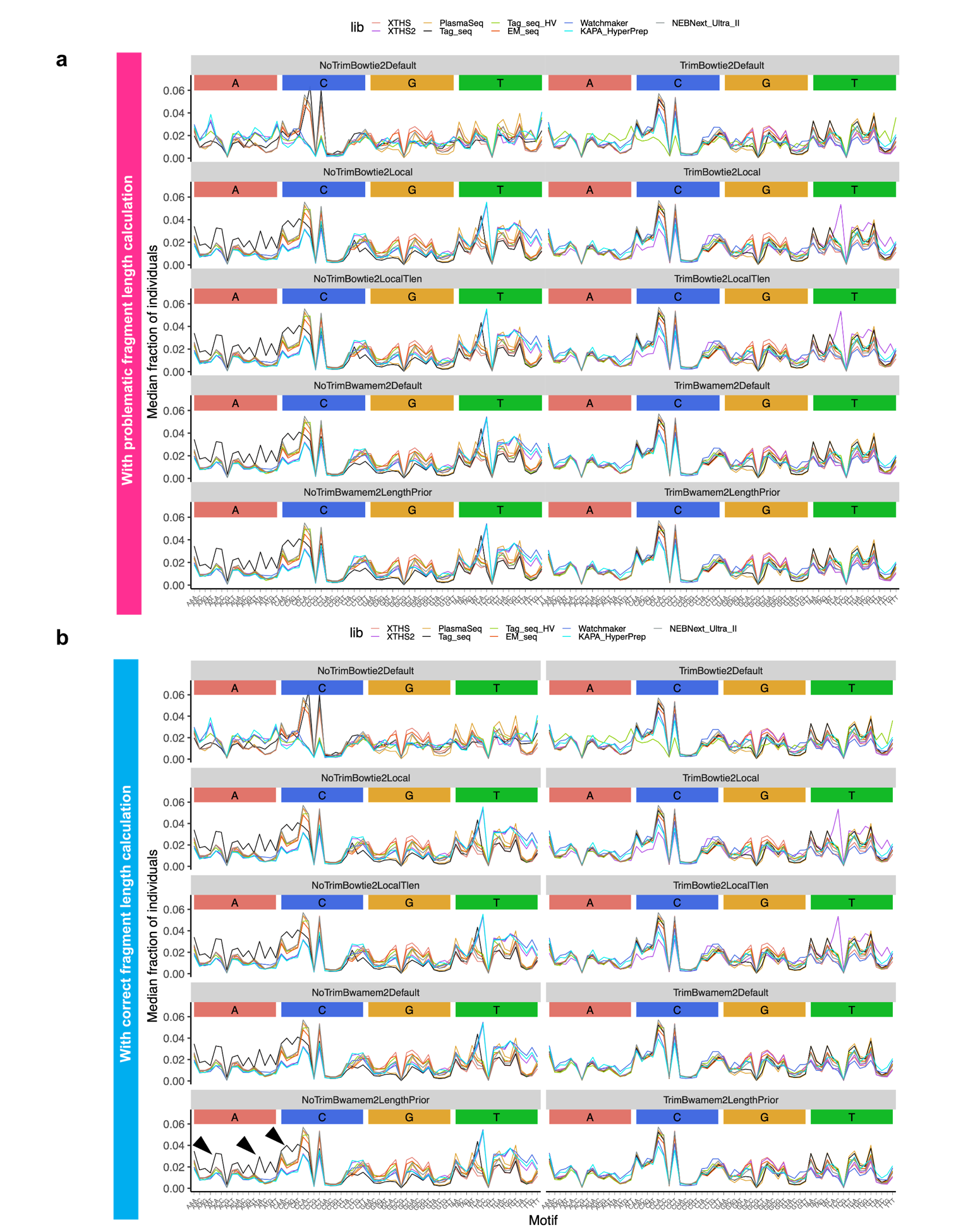


[**Fig S16**](#sfigu_motif_facet_by_params) **Median motif distribution, facets are parameters, lines in each panel are library preparation kits**. **a** With problematic fragment length calculation step. **b** With correct fragment length calculation. Black triangles highlight the abnormal motifs of ThruPLEX Tag-Seq in untrimmed s3 motif distribution. And the Watchmaker in both trimmed and untrimmed s3 motif distributions. Heatmaps showing the same data is shown in [**Additional Fig S17**](#sfi_motif_facet_by_params_heatmap).


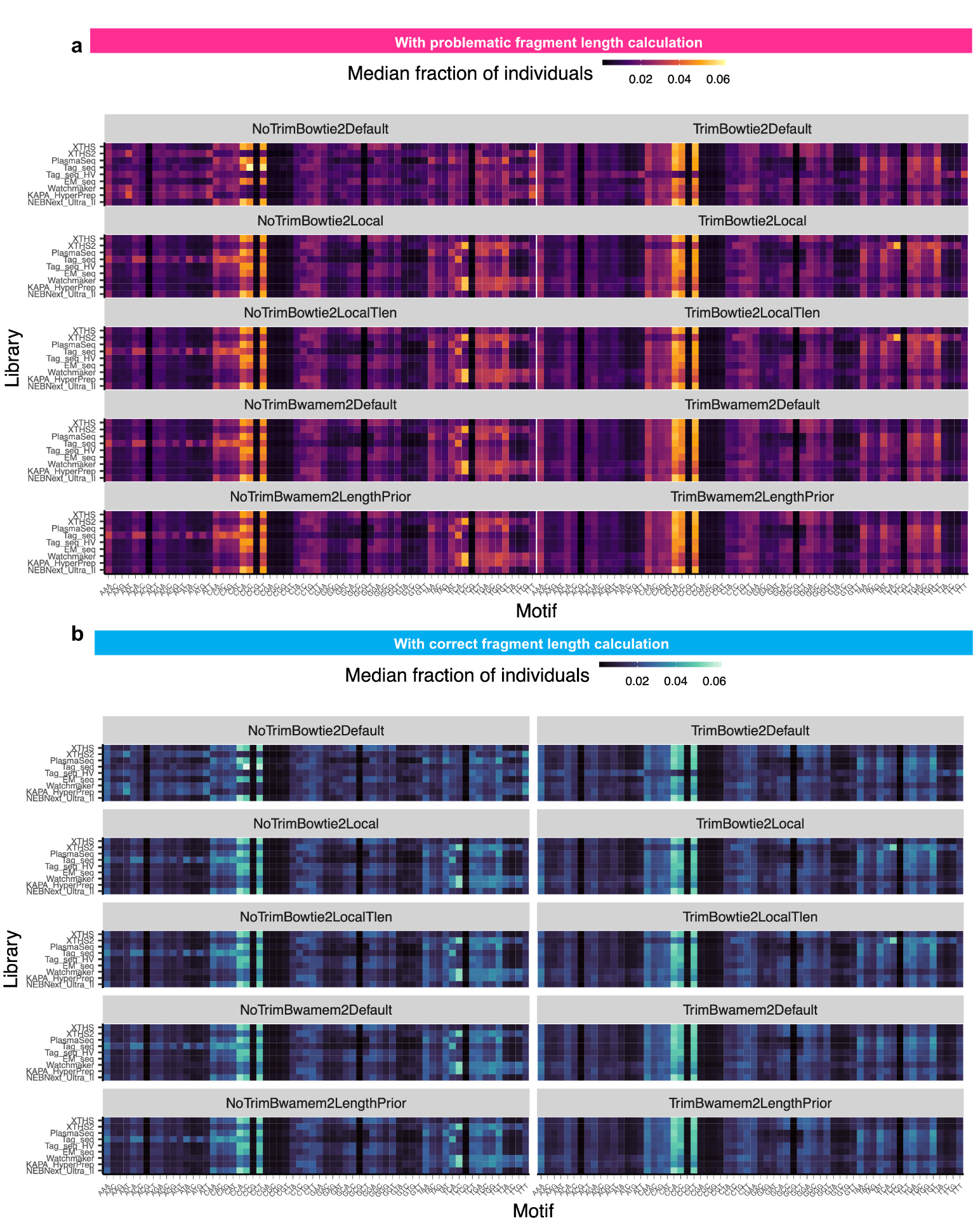


[**Fig S17**](#sfigu_motif_facet_by_params_heatmap) **Heatmap of median motif distribution**. facets are ten analytical parameter settings, x-axis shows 64 s3 motifs, y-axis shows library kits. **a** With problematic fragment length calculation step. **b** With correct fragment length calculation. Line plots showing the same data is shown in [**Additional Fig S16**](#sfi_motif_facet_by_params).


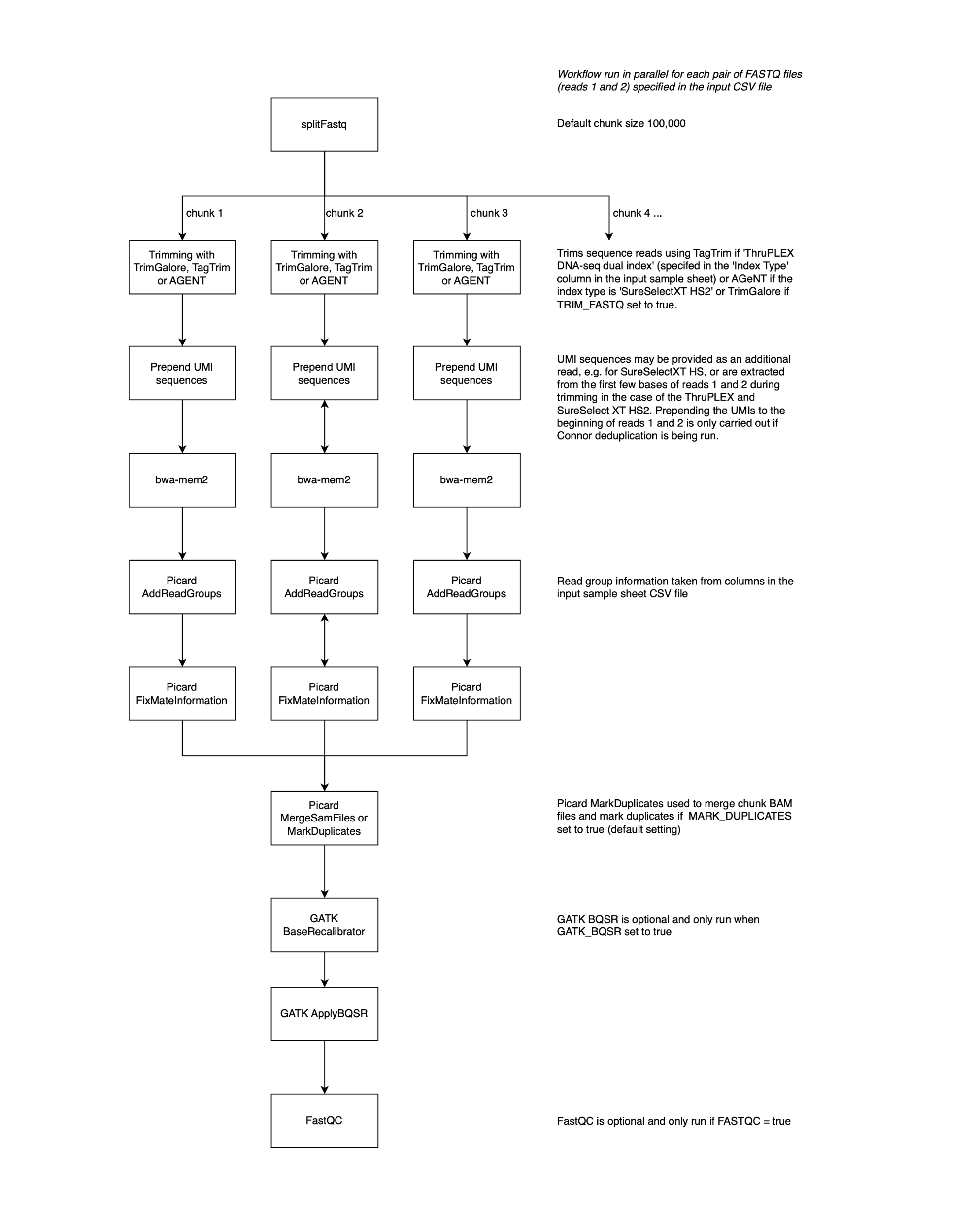


[**Fig S18**](#sfigu_tap) **Workflow of Trim Align Pipeline (TAP).** The entire TAP pipeline can be accessed via GitHub: <https://github.com/nrlab-CRUK/TAP>


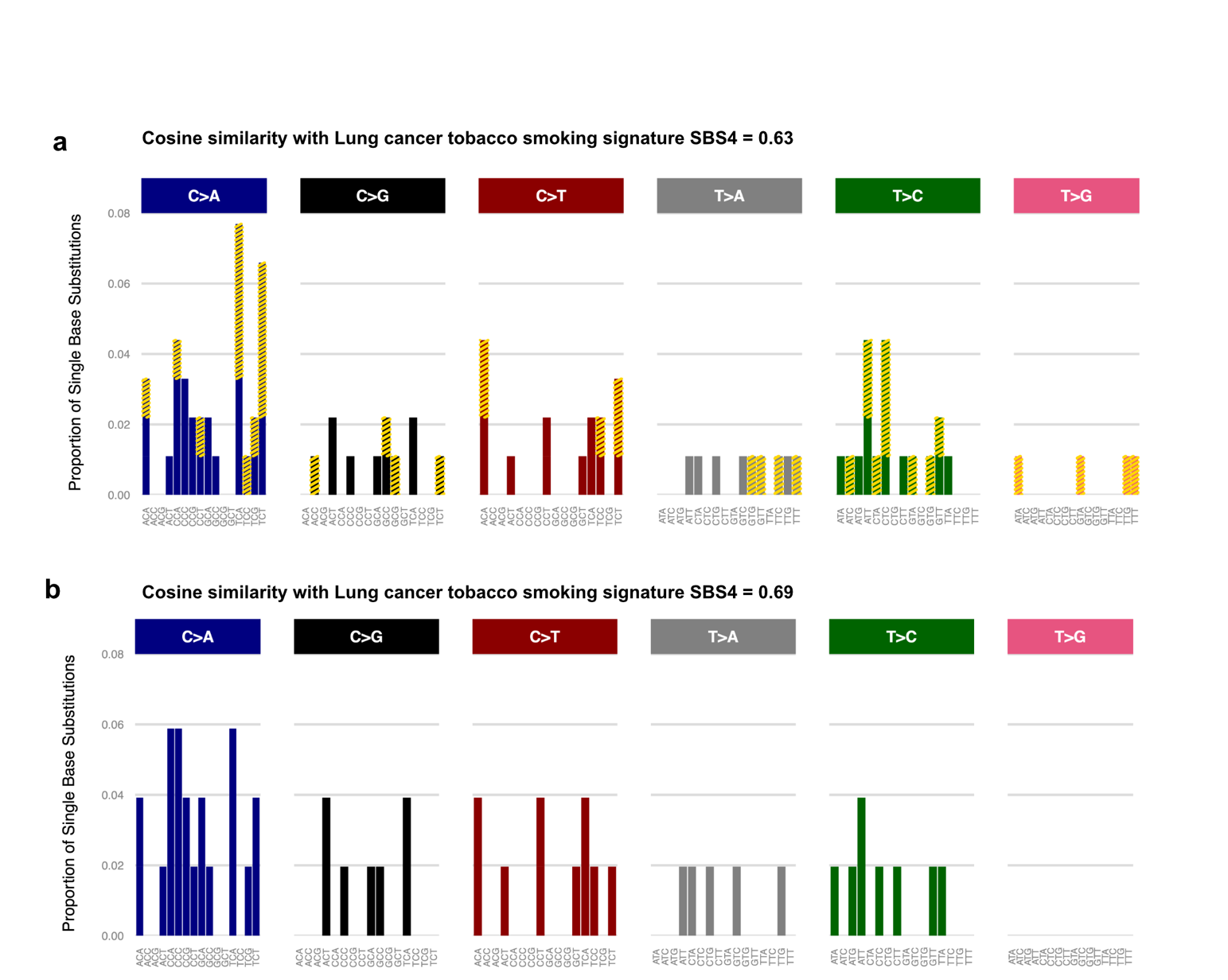


[**Fig S19**](#sfigu_mut_corr) **Demonstration of mutation type filtering**. **a** DO substitution in the mutation signature profile included in the analysis (highlighted using a red rectangle with yellow stripes), the cosine similarity between the sample and SBS4 (i.e. tobacco smoking signatures) is 0.63. **b** By removing the SO substitutions, the similarly increased to 0.69.


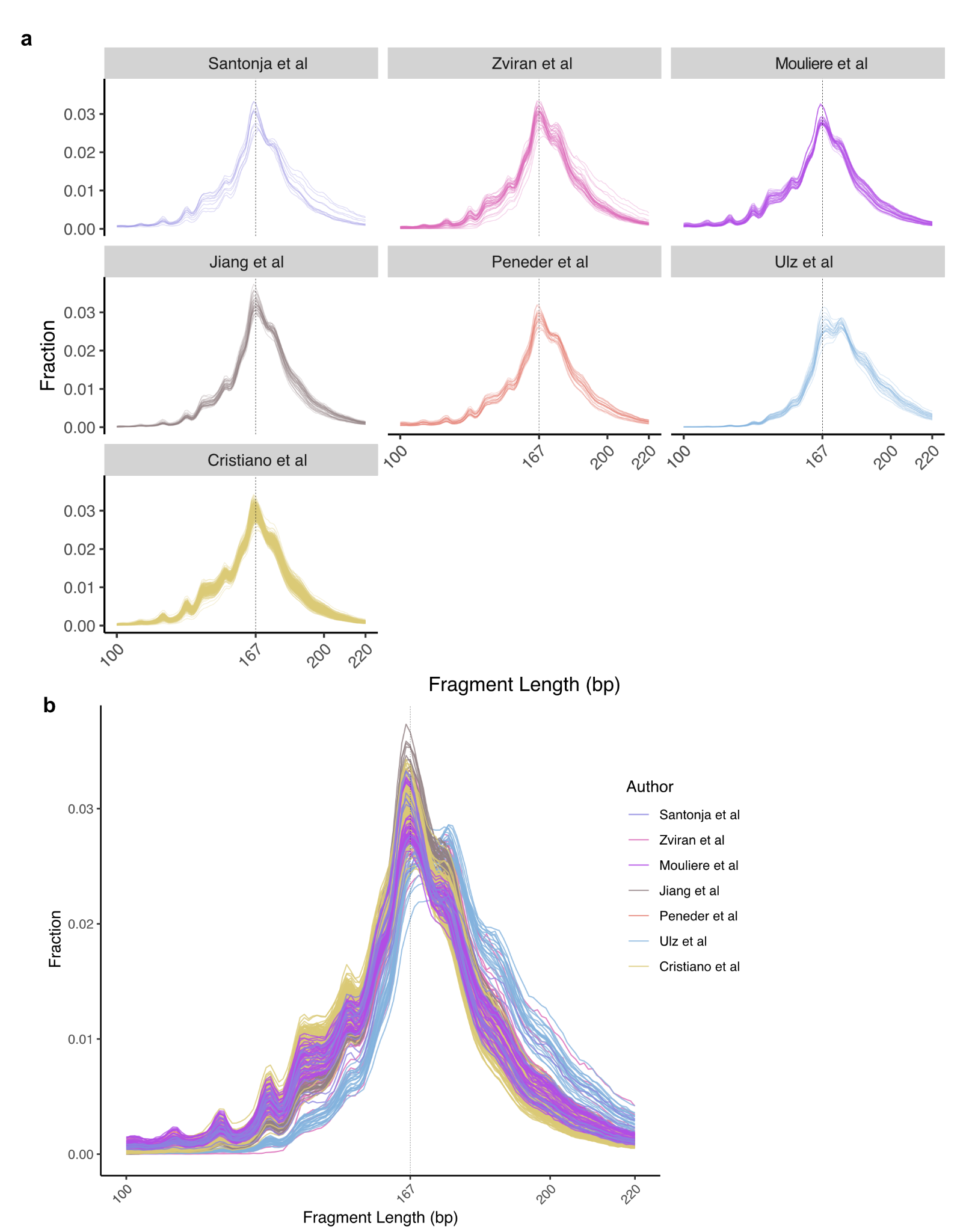


[**Fig S20**](#sfigu_external_len_dist) **Fragment length distribution (100-220bp) of samples from various studies**. **a** Length distribution samples split based on study (i.e., author). **b** Length distribution of samples shown together. Each line represents one individual.


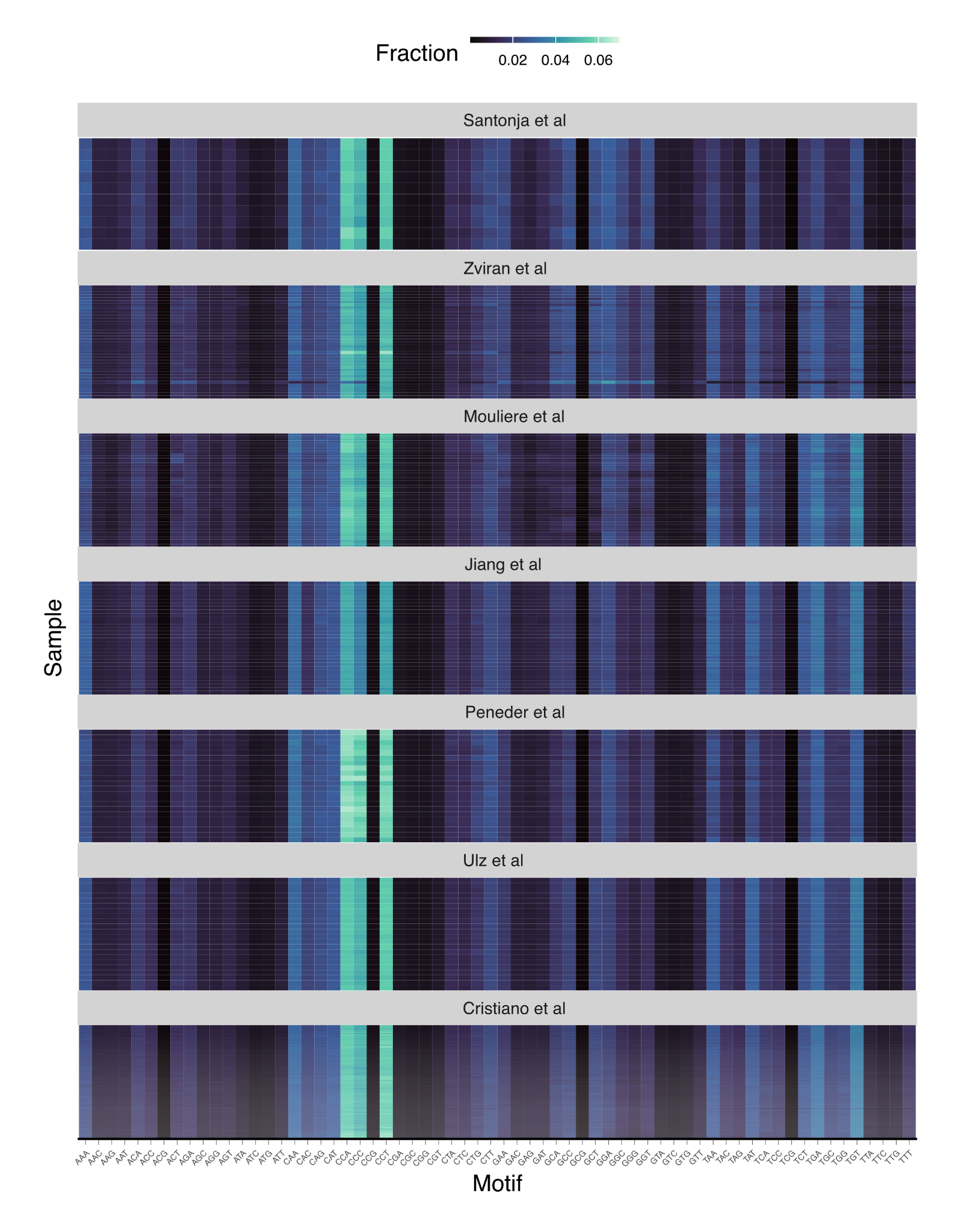


[**Fig S21**](#sfigu_external_motif_dist) **Heatmap showing the s3 motif fraction of samples from various studies.**


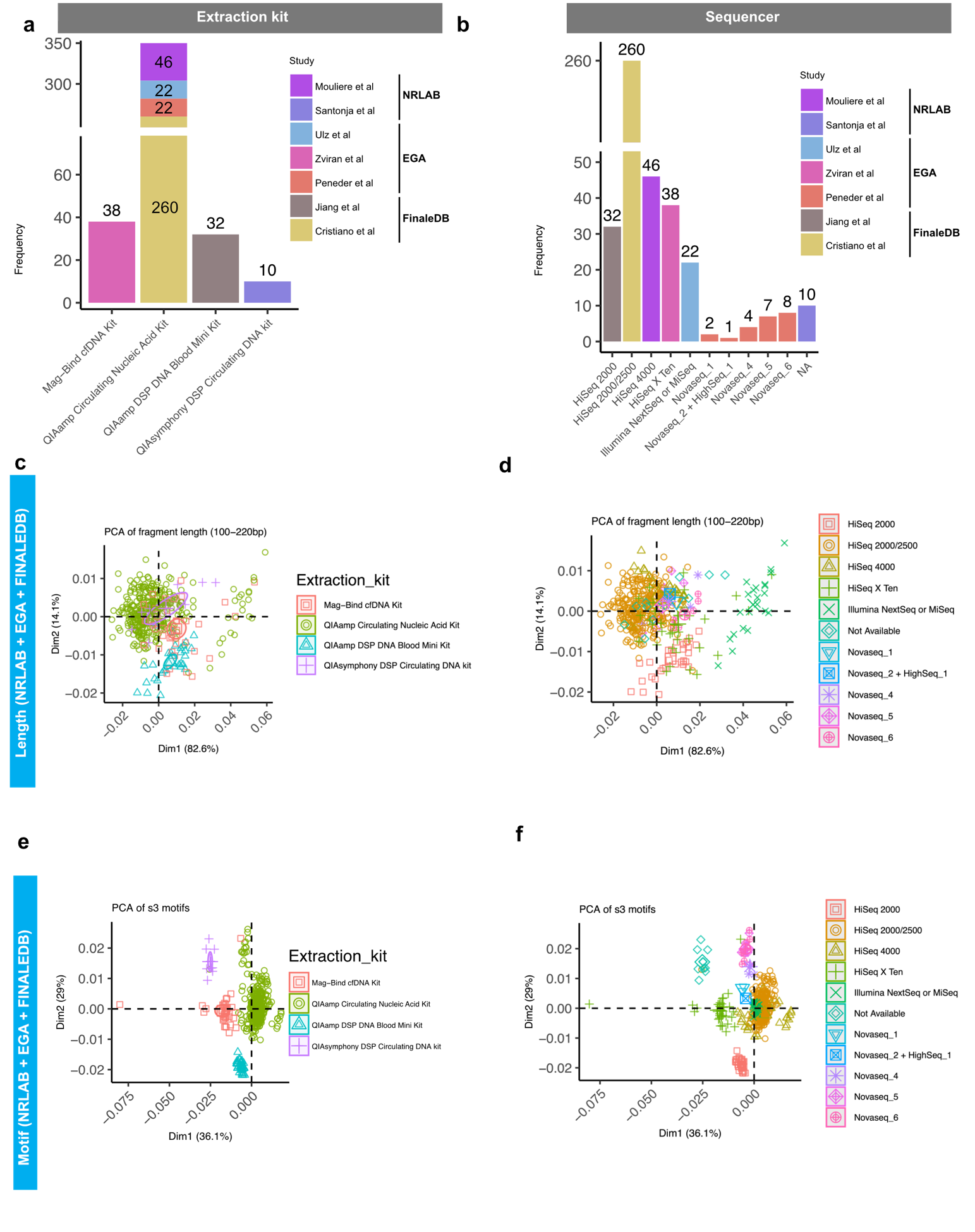


[**Fig S22**](#sfigu_external1) **Statistics of extraction kit and sequencers reported by various studies. a** Stacked bar chart showing DNA extraction kits used by various studies. **b** Data source. **c** and **e** PCA of fragment lengths and s3 motifs (grouped by extraction kit). **d** and **f** PCA analysis of lengths and s3 motifs (grouped by sequencing platforms)


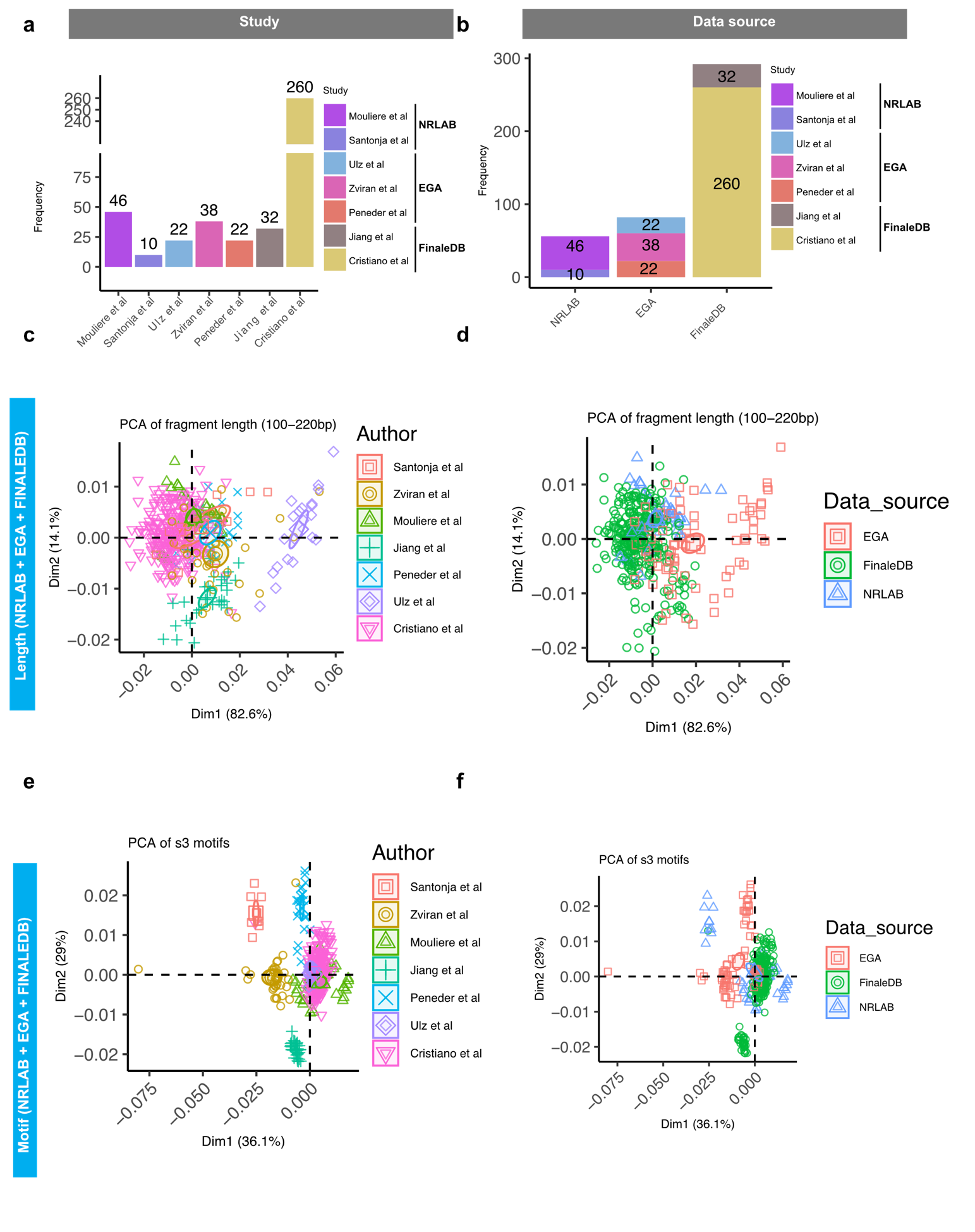


[**Fig S23**](#sfigu_external_cohort_and_datasource) **Samples number and data source of healthy plasma samples from various studies.** **a** Number of samples in each study. **b** Data source. **c** and **e** Length and s3 motifs PCA analyses (grouped by authors/studies). **d** and **f** Length and s3 motifs PCA analyses (grouped by data source).


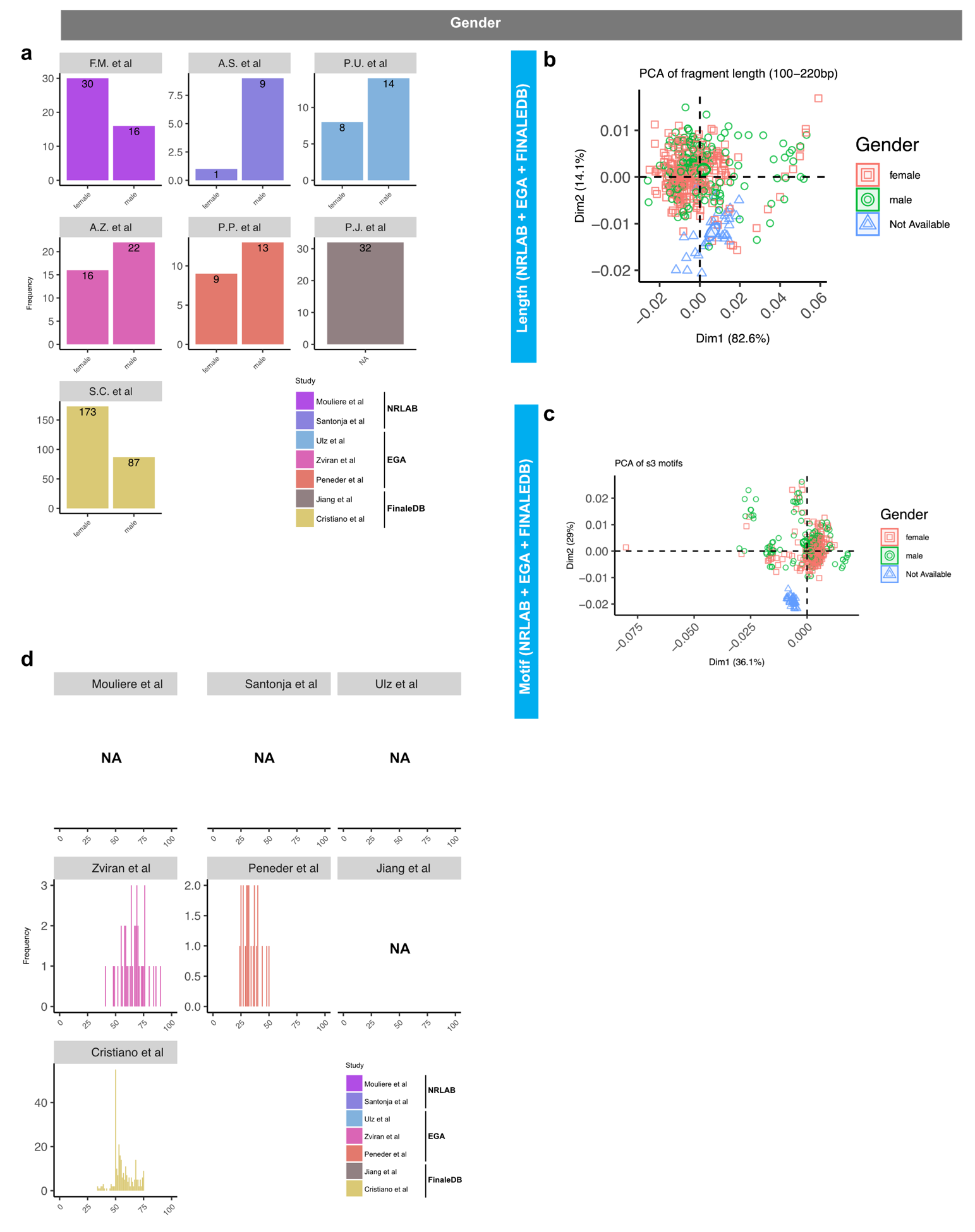


[**Fig S24**](#sfigu_external_others) **Gender and age information**. **a** Gender information of samples collected in those studies. **b** and **c** PCA analysis of length and s3 motifs (grouped by gender). **d** Age information of samples collected in various studies.


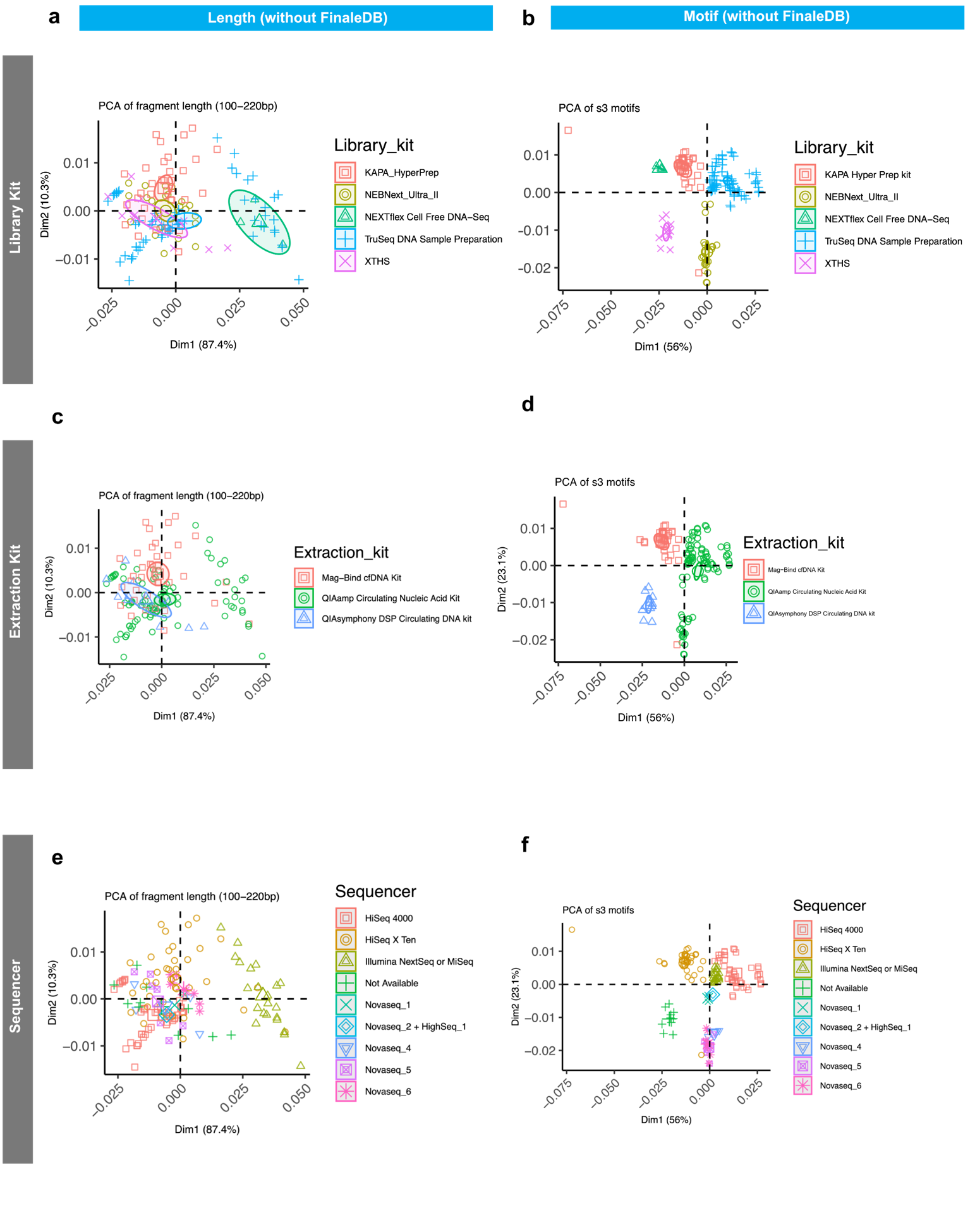


[**Fig S25**](#sfigu_external_no_finaledb1) **PCA analysis of NRLAB and EGA** (i.e. excluding FinaleDB data due to heterogeneous data processing pipeline). **a**, **c** and **e** The PCA based on fragment lengths and grouped by “Library Kit”, “Extraction Kit” and “Sequencer” respectively. **b**, **d**, and **f** The PCA of fragment s3 motifs and grouped by “Library Kit”, “Extraction Kit” and “Sequencer” respectively.


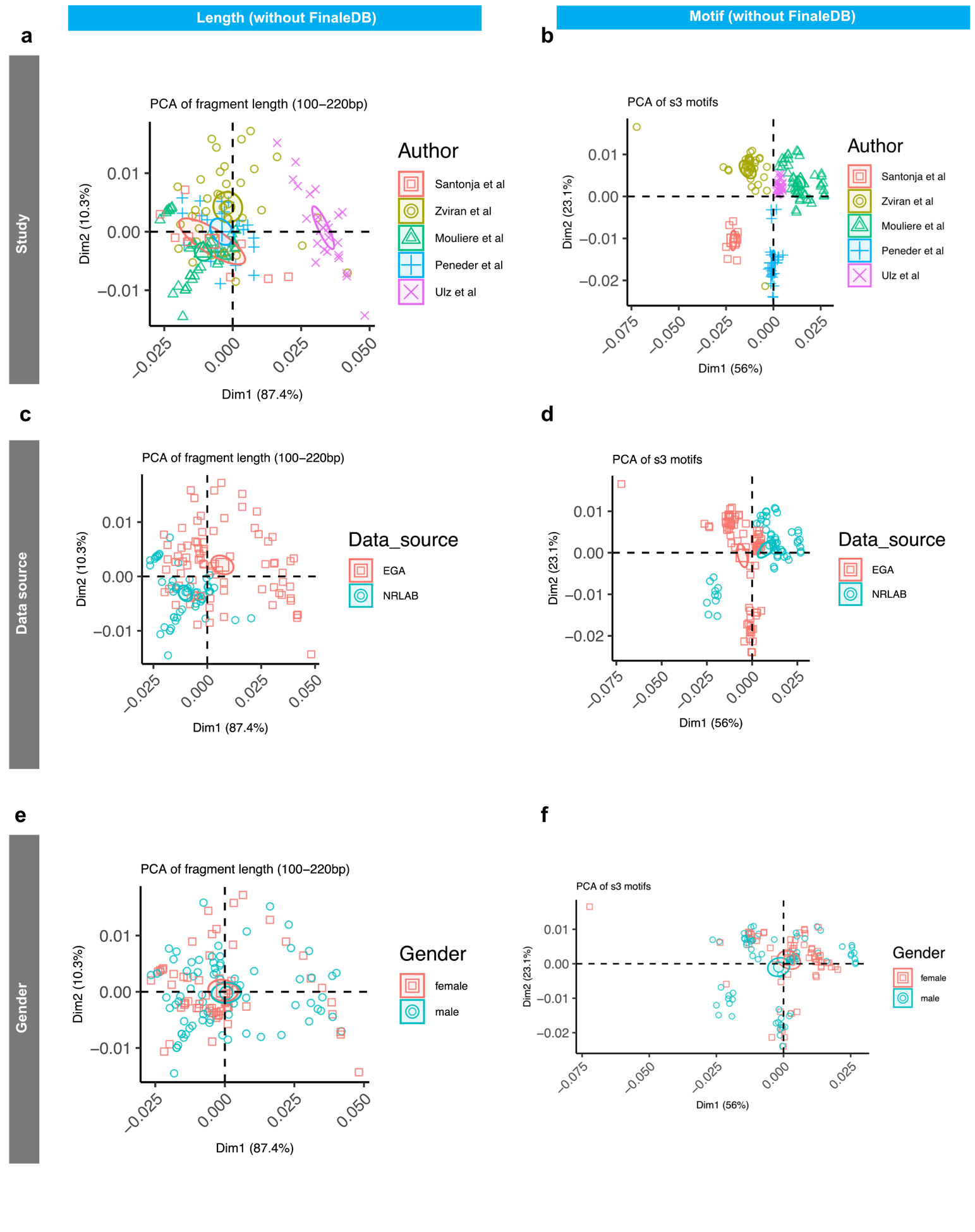


[**Fig S26**](#sfigu_external_no_finaledb2) **PCA analysis of NRLAB and EGA** (i.e. excluding FinaleDB data due to heterogeneous data processing pipeline). **a**, **c** and **e** The PCA based on fragment lengths and grouped by “Study”, “Data source ” and “Gender” respectively. **b**, **d**, and **f** The PCA of fragment s3 motifs and grouped by “Study”, “Data source ” and “Gender” respectively.


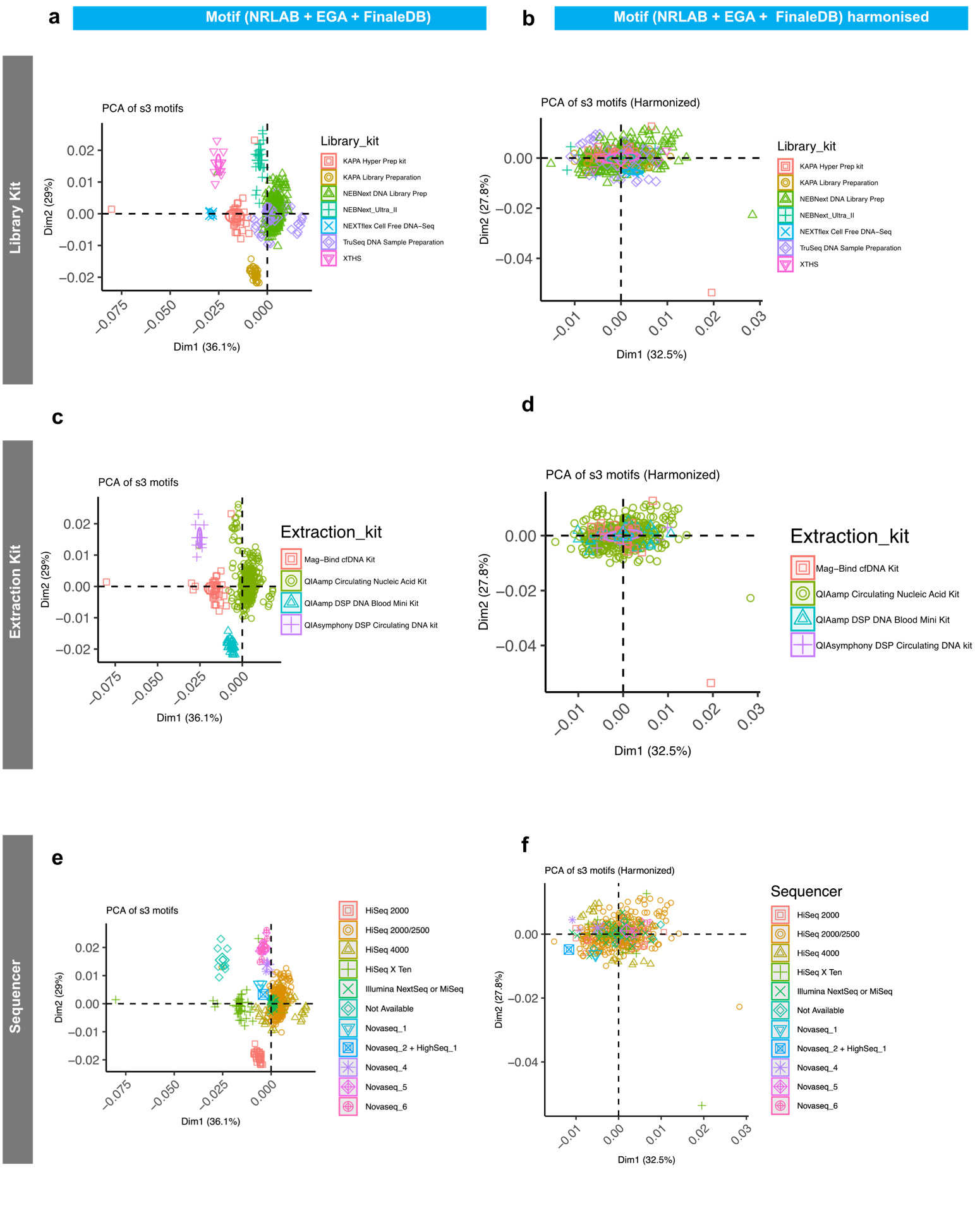


[**Fig S27**](#sfigu_external1_harmonised_motif) **PCA analysis of NRLAB and EGA**. **a**, **c** and **e** The PCA based on fragment s3 motifs and grouped by “Library Kit”, “Extraction Kit” and “Sequencer” respectively. **b**, **d**, and **f** The PCA of harmonised fraction of s3 motifs.


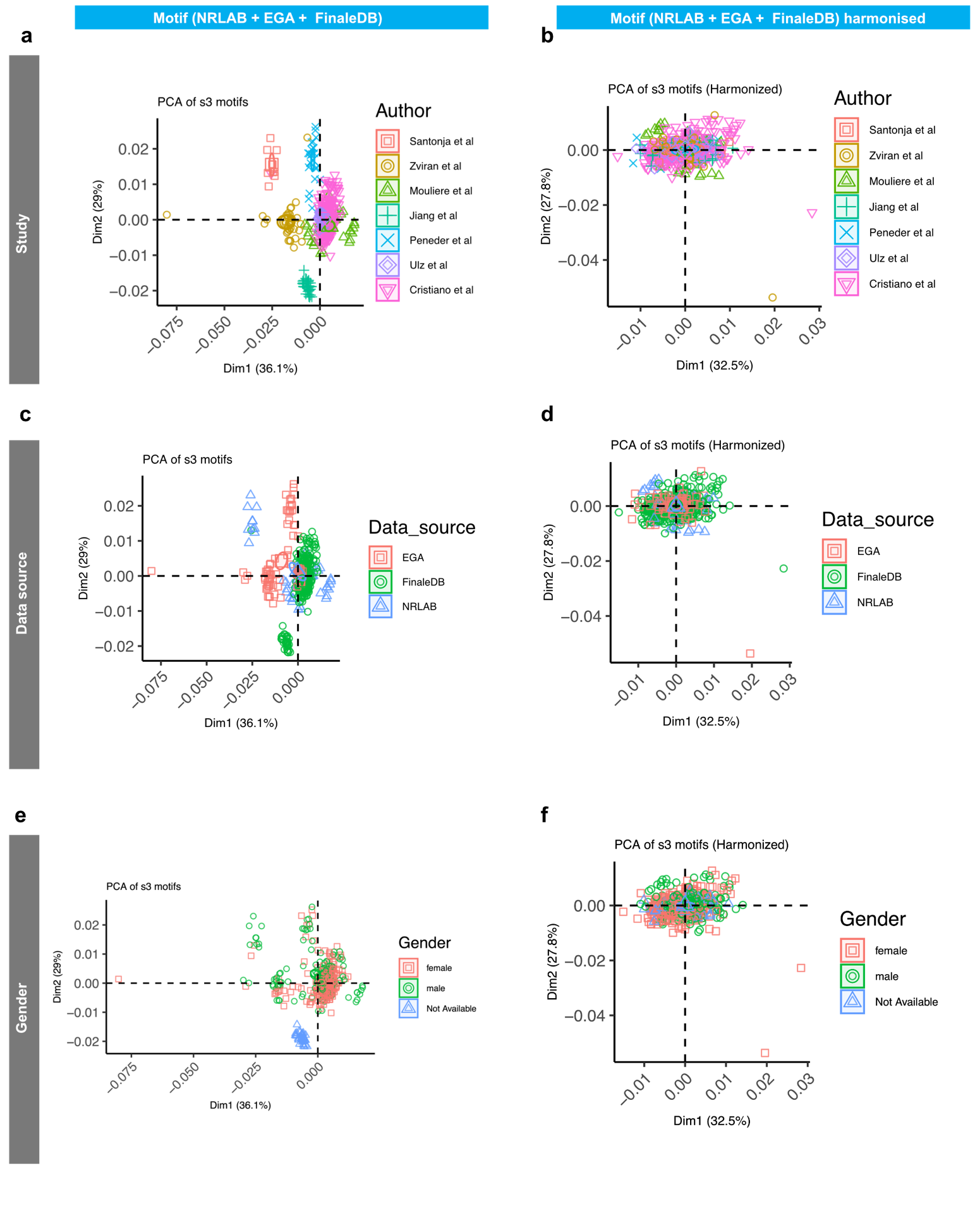


[**Fig S28**](#sfigu_external2_harmonised_motif) **PCA analysis of NRLAB, EGA and FinaleDB**. **a**, **c** and **e** The PCA based on fragment s3 motifs and grouped by “Study”, “Data source ” and “Gender” respectively. **b**, **d**, and **f** The PCA of harmonised fraction of s3 motifs.


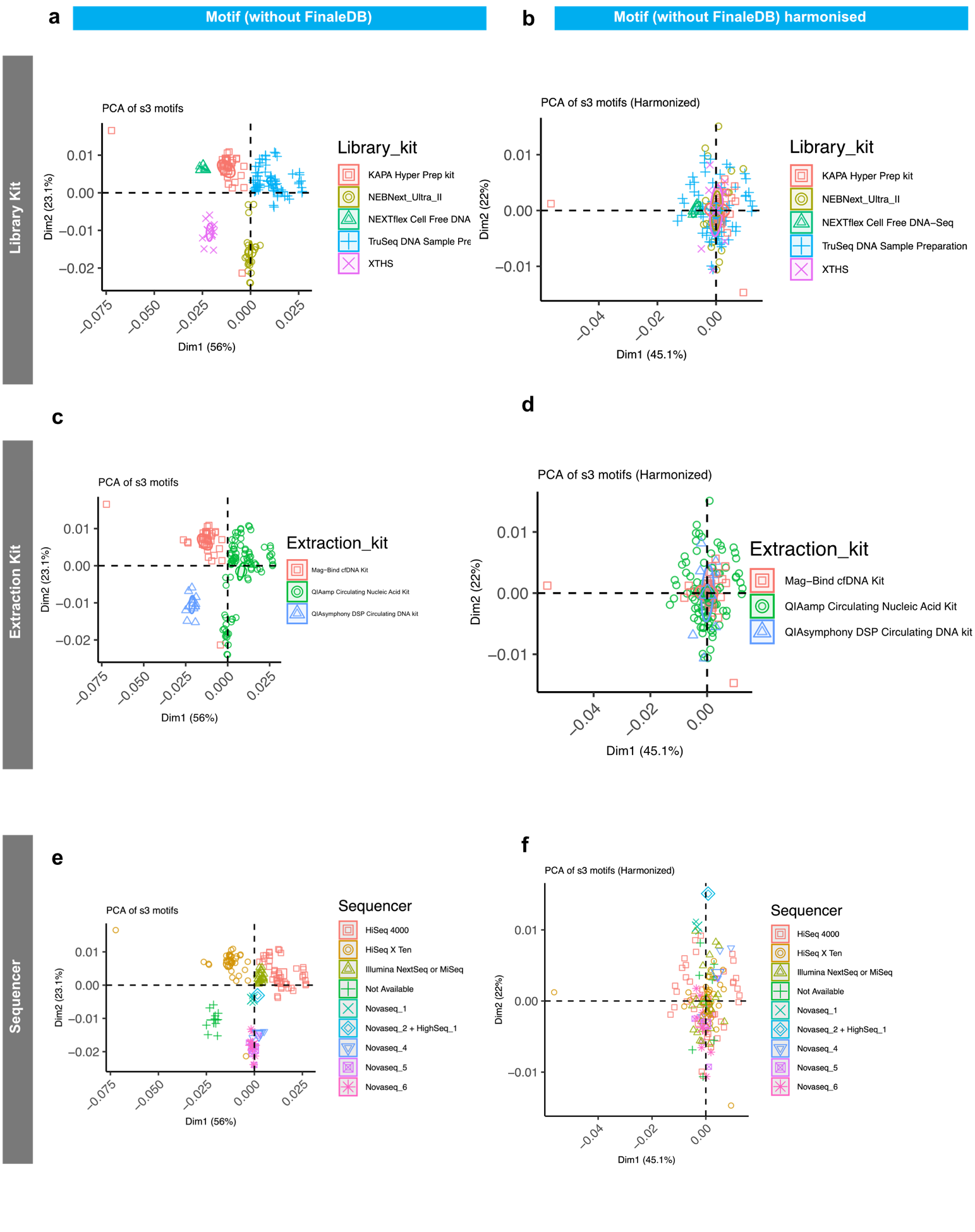


[**Fig S29**](#sfigu_external1_no_finaledb_harmonised_motif) **PCA analysis of NRLAB and EGA**. **a**, **c** and **e** The PCA based on fragment s3 motifs and grouped by “Library Kit”, “Extraction Kit” and “Sequencer” respectively. **b**, **d**, and **f** The PCA of harmonised fraction of s3 motifs.


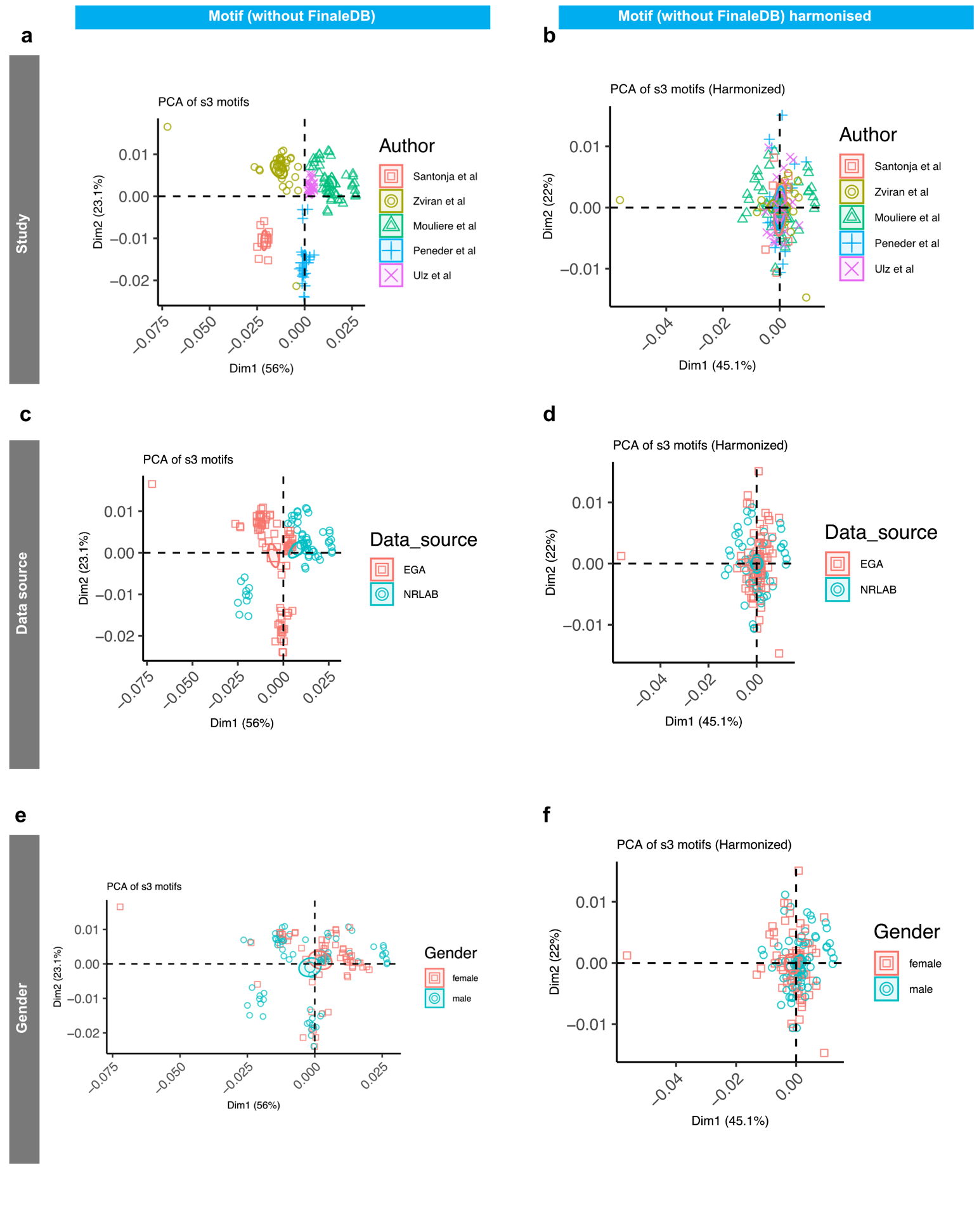


[**Fig S30**](#sfigu_external2_no_finaledb_harmonised_motif) **PCA analysis of NRLAB and EGA**. **a**, **c** and **e** The PCA based on fragment s3 motifs and grouped by “Study”, “Data source ” and “Gender” respectively. **b**, **d**, and **f** The PCA of harmonised fraction of s3 motifs.


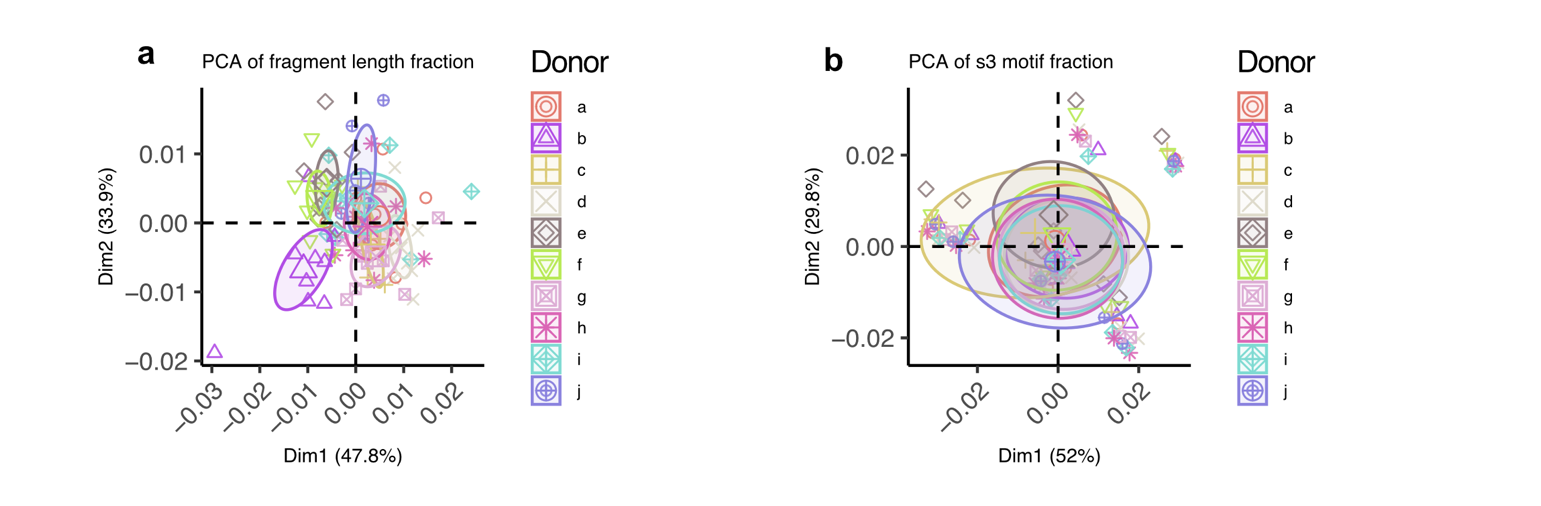


[**Fig S31**](#sfigu_internal_pca_pid) PCA analysis of fragment length and motif fraction grouped by healthy donor IDs. **a** PCA of fragment length fraction. **b** PCA of fragment s3 motif fraction.


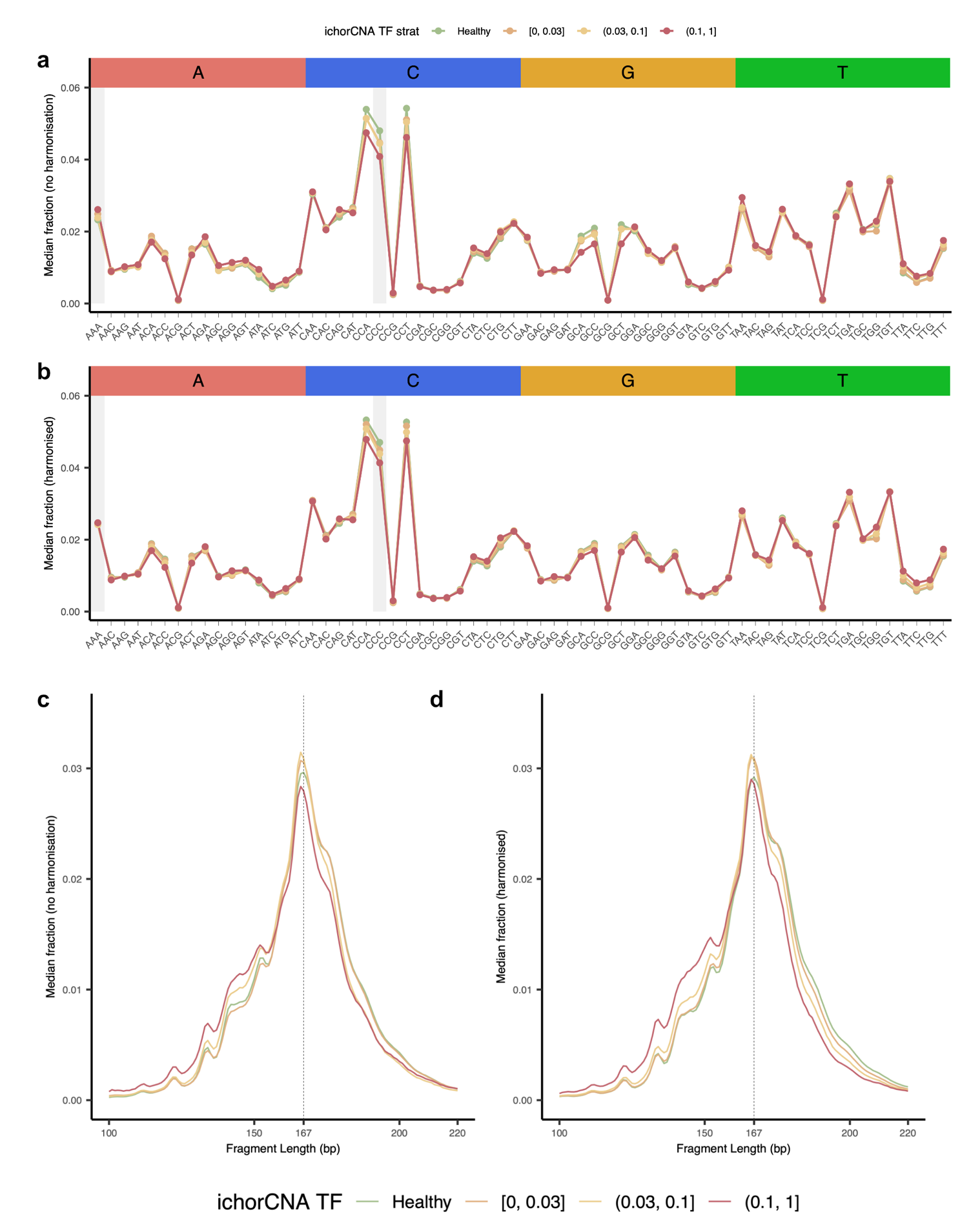


**Fig S32** **a-b** The median fraction of s3 motifs of samples in different ichorCNA TF groups before and after harmonisation. **c-d** he median fraction of lengths of samples in different ichorCNA TF groups before and after harmonisation.


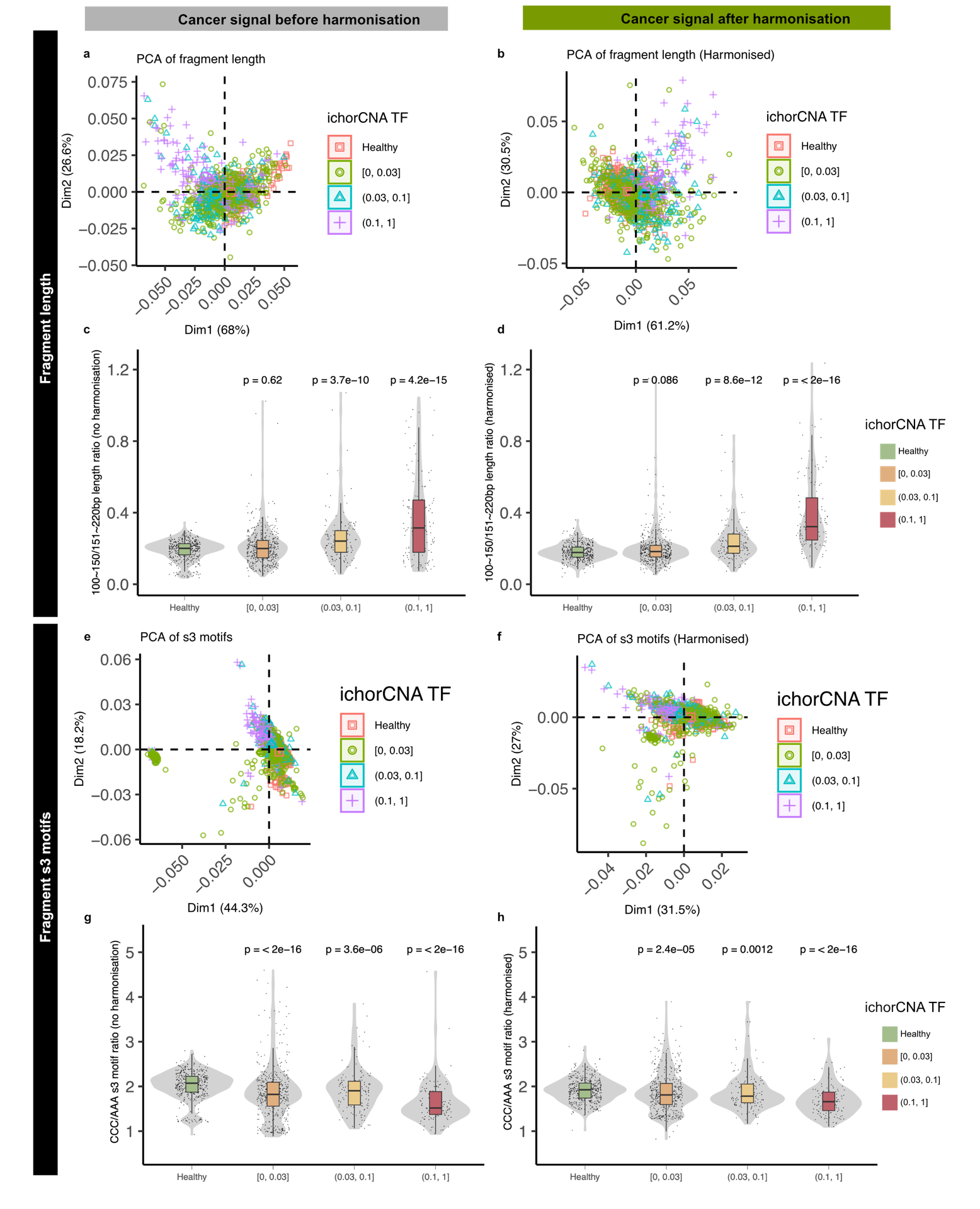


**Fig S33 Comparison of cancer signals before (left) and after (right) feature harmonisation.** **a-b** The PCA analysis of fragment lengths. **c-d** The cancer signal reflected by 100-150/151-220bp fragment length ratio across ichorCNA TF groups. **e-f** PCA analysis of fragment s3 motifs. **g-h** The cancer signal reflected by CCC/AAA s3 motif ratio across ichorCNA TF groups. For each PCA plot, 95% confidence area surrounding the group mean value was shown by ellipses. The median distributions of s3 motif and length in each ichorCNA TF group (before and after harmonisation) are shown in **Additional Fig S32**.
