# Supplementary material for: A standardized framework for robust fragmentomic feature extraction from cell-free DNA sequencing data
Source: Genome Biol. 2025 May 23;26:141. doi: 10.1186/s13059-025-03607-5 (PMC12100915; doi:10.1186/s13059-025-03607-5)
Supplement: Supplementary file 2 — Additional file 2. A file containing additional Tables S1–S7 [file 13059_2025_3607_MOESM2_ESM.docx]

# Additional File 2

**Wang et al**

**The file includes:**

**Additional Table S1**-**S7**

## Additional tables

[**Table S1**](#stabl_barcodes) **Healthy donor ID and name of sample barcodes of sequencing libraries.** Blank cells indicate the exclusion of the samples from analyses due to a lack of DNA materials.

| Donor | Specimen | XTHS | XTHS2 | Tag_seq | Tag_seq_HV | EM_seq | PlasmaSeq | Watchmaker | KAPA_HyperPrep | NEBNext_Ultra_II |
| --- | --- | --- | --- | --- | --- | --- | --- | --- | --- | --- |
| **a** | PPC10a |  | P1A01 | D701tp-D501tp | UDI0065dna | A9 | U15 | A2 | UDI-P09 | NEBi701-i503 |
| **b** | PPC10b | SXTHSE02 | P1B01 | D701tp-D502tp | UDI0066dna | B9 | U16 | B2 | UDI-P10 | NEBi702-i503 |
| **c** | PPC10c |  | P1C01 | D701tp-D503tp | UDI0067dna |  | U17 | C2 |  |  |
| **d** | PPC10d | SXTHSH03 | P1D01 | D701tp-D504tp | UDI0068dna | C9 | U18 | D2 | UDI-P11 | NEBi703-i503 |
| **e** | PPC10e | SXTHSA04 | P1E01 | D702tp-D501tp | UDI0073dna | D9 | U19 | E2 | UDI-P12 | NEBi704-i503 |
| **f** | PPC10f | SXTHSB04 | P1F01 | D702tp-D502tp | UDI0074dna | E9 | U20 | D1 | UDI-P13 | NEBi701-i504 |
| **g** | PPC10g | SXTHSC04 | P1G01 | D702tp-D503tp | UDI0075dna | F9 | U21 | E1 | UDI-P14 | NEBi702-i504 |
| **h** | PPC10h | SXTHSD04 | P1H01 | D702tp-D504tp | UDI0076dna | G9 | U22 | F1 | UDI-P15 | NEBi703-i504 |
| **i** | PPC10i | SXTHSE04 | P1A02 | D701tp-D507tp | UDI0072dna | H9 | U23 | G1 | UDI-P16 | NEBi704-i504 |
| **j** | PPC10j | SXTHSF04 | P1B02 | D702tp-D508tp | UDI0080dna |  | U24 | H1 |  |  |

[**Table S2**](#stabl_software) **Software/tools/packages in this paper.**

| Software in data trimming and alignment | Version | Source |
| --- | --- | --- |
| *A nextflow pipeline (with docker container) was built and accessible via :* [*https://github.com/nrlab-CRUK/TAP*](https://github.com/nrlab-CRUK/TAP/tree/master) | | |
| miniconda | py39_4.12.0 | [repo.anaconda.com](https://repo.anaconda.com/miniconda/Miniconda3-py39_4.12.0-Linux-x86_64.sh) |
| python | 3.6 | conda-forge |
| mysql-connector-python | 8 | conda-forge |
| bamtools | 2.5.2 | bioconda |
| bedtools | 2.30.0 | bioconda |
| biopython | 1.7 | bioconda |
| bowtie2 | 2.5.1 | bioconda |
| bwa-mem2 | 2.2.1 | bioconda |
| bwameth | 0.2.6 | bioconda |
| fastqc | 0.11.9 | bioconda |
| gatk4 | 4.2.6.1 | bioconda |
| hmmcopy | 0.1.1 | bioconda |
| picard | 2.27.4 | bioconda |
| pysam | 0.19 | bioconda |
| samtools | 1.15.1 | bioconda |
| trim-galore | 0.6.7 | bioconda |
| trimmomatic | 0.39 | bioconda |
| r-base | 4.1.3 | conda-forge |
| r-optparse | 1.7.3 | conda-forge |
| r-tidyverse | 1.3.2 | conda-forge |
| r-ichorcna | 0.3.2 | bioconda |
| groovy | 4.0.15 | <https://groovy.jfrog.io/artifactory/dist-release-local/groovy-zips/> |
| The Agilent Genomics NextGen Toolkit (AGeNT) | 3.0.5 | [Agilent](https://www.agilent.com/en/download-agent-tool?productURL=https%3A%2F%2Fwww.agilent.com%2Fen%2Fproduct%2Fnext-generation-sequencing%2Fngs-data-analysis-interpretation%2Fagent-4301558) |
| connor | 0.6.1 | bioconda |
| pysam | 0.11.2.2 | bioconda |
|  |  |  |
| Software in data analysis | Version | Source |
| *Platform: x86_64-conda-linux-gnu (64-bit), Running under: CentOS Linux 7 (Core)* | | |
| r-base | 4.3.2 | conda-forge |
| cfDNAPro | 1.7.1 | <https://github.com/hw538/cfDNAPro> |
| r-tidyverse | 2.0.0 | conda-forge |
| factoextra | 1.0.7 | <https://cran.r-project.org/web/packages/factoextra/index.html> |
| sva | 3.50.0 | Bioconductor |

[**Table S3**](#bookmark=id.1baon6m) **Functions implemented in cfDNAPro.** The detailed documentation of each function could be accessed via the R console or additional file.

| Category and Function | Description | Input/output |
| --- | --- | --- |
| readBam | Import bam file as GRanges, including QC, curation, and mutational annotation | Bam file/GRanges obj |
| readGALP | Import bam file as GAlignmentPairs object | Bam file/GAlignmentPairs obj |
| callLength | Calculate fragment length | GRanges/Tibble obj |
| callMotif | Calculate the frequency or fraction of motifs | GRanges/Tibble |
| callTrinucleotide | Calculate trinucleotide mutation frequencies | GRanges/Tibble |
| callCNV | Calculate copy number variation | GRanges/Tibble |
| callMetrics | Calculate the median/mean proportion of each fragment length within cohorts | Bam files/Tibble |
| callMode | Calculate the modal fragment size of each sample | Bam files/Tibble |
| callPeakDistancce | Calculate the inter-peak distance of the fragmentation profile | Bam files/Tibble |
| callValleyDistance | Calculate the inter-trough distance of the fragmentation profile | Bam files/Tibble |
| plotLength | Plot fragment length distribution | Tibble/ggplot2 obj |
| plotMotif | Plot the frequency of different motifs | Tibble/ggplot2 |
| plotTrinucleotide | Plot trinucleotide mutation counts | Tibble/ggplot2 |
| plotCNV | Plot copy number variation | Tibble/ggplot2 |
| plotMetrics | Plot the fragment length metrics of multiple cohorts | Tibble/list of ggplot2 obj |
| plotMode | Plot the modal fragment length of multiple cohorts | Tibble/ggplot2 |
| plotModeSummary | Plot modal fragment size in a stacked bar chart of multiple cohorts | Tibble/ggplot2 |
| plotSingleGroup | Plot the raw fragment size metrics of a single group in a single plot | Tibble/ggplot2 |
| plotPeakDistance | Plot the distribution of inter-peak distance | Tibble/ggplot2 |
| plotValleyDistance | Plot the distribution of inter-valley distance | Tibble/ggplot2 |
| summarizeBam/summariseBam | Summarise descriptive Bam stats, e.g. depth, n mapped reads, etc. | Bam file/Tibble |
| downsampleBam | Randomly downsample bam file to target depth or number of read pairs | Bam file/GRanges or Bam file |
| examplePath | Get the path to built-in example data | Not Applicable/String |

| Name | Peer-reviewed^1^ | Latest update^2^ | Language | Link to repository | Description^3^ |
| --- | --- | --- | --- | --- | --- |
| FinaleToolkit | No (preprint: Li et al, bioRxiv, 2024) | 9 Jan 2025 | Python | <https://github.com/epifluidlab/FinaleToolkit> | FinaleToolkit (FragmentatIoN AnaLysis of cEll-free DNA Toolkit) is a package and standalone program to extract fragmentation features of cell-free DNA from paired-end sequencing data. |
| cfDNA-UniFlow | No | 24 Sep 2024 | Snakemake | <https://github.com/kircherlab/cfDNA-UniFlow> | cfDNA-UniFlow: a unified, standardized, and ready-to-use workflow for processing whole genome sequencing (WGS) cfDNA samples from liquid biopsies. |
| cfDNApipe | Yes (Zhang et al, Bioinformatics, 2021) | 18 Jan 2022 | Python | <https://github.com/XWangLabTHU/cfDNApipe> | cfDNApipe (cell free DNA Pipeline) is an integrated pipeline for analyzing cell-free DNA WGBS/WGS data. |
| cfDNA-Flow | No | 14 Jun 2024 | Snakemake | <https://github.com/uzh-dqbm-cmi/cfDNA-Flow> | cfDNA-Flow facilitates the accurate and reproducible analysis of cfDNA WGS data. It offers various preprocessing options to accommodate different experimental setups and research needs in the field of liquid biopsies. |
| cfTools | No | 10 Aug 2024 | R/ | <https://github.com/jasminezhoulab/cfTools> | cfTools is an R package for cell-free DNA (cfDNA) methylation data analysis, including (1) cancer detection: sensitively detect tumor-derived cfDNA and estimate the tumor-derived cfDNA fraction (tumor burden); (2) tissue deconvolution: infer the tissue type composition and the cfDNA fraction of multiple tissue types for a plasma cfDNA sample. |
| cfdnakit | No | 30 Sep 2024 | R | <https://github.com/Pitithat-pu/cfdnakit> | This package provides basic functions for analyzing next-generation sequencing of circulating cell-free DNA (cfDNA). The package focuses on extracting length of cfDNA, and genome-wide copy-number alteration estimated by the short-fragmented cfDNA using shallow whole-genome sequencing data (~0.3X or more). The ctDNA estimation score (CES) comprehensively estimate the circulating tumor DNA based on the short-fragment analysis. |
| cfDNAPro | This manuscript | 11 Jan 2025 | R | [https://github.com/nrlab-CRUK/cfDNAPro](https://github.com/hw538/cfDNAPro) | **Derived from an in-depth evaluation of library kit biases on fragmentomic features, the methods implemented in package include** the fragment length, motif, copy number, and mutation signature analysis from experts within the cfDNA study area. Robust and reproducible feature extraction process which minimises the potential biases called by different processing pipelines. Together with Trim Align Pipeline (TAP), the analysis process ensures the correct quantification of features. |

**Table S4** A list of bioinformatic analysis tools for cfDNA research. Data was retrieved on 11 Jan 2025.

^1^ Providing the information about if the tool has published as peer-reviewed papers as of 11 Jan 2025.

^2^ This is the date of latest commit to GitHub repository.

^3^ This is the tool description obtained from its code repository.

**Table S5** The number of samples collated from published studies and analysed in this manuscript. Cancer samples were stratified into three groups based on ichorCNA TF. Square brackets indicate inclusivity of boundaries, while parentheses indicate exclusivity of boundaries.

| Data source | Study | Healthy | [0, 0.03] | (0.1, 1] | (0.03, 0.1] |
| --- | --- | --- | --- | --- | --- |
| NRLAB | Mouliere et al, 2018 | 46 | 126 | 45 | 23 |
| NRLAB | Santonja et al, 2023 | 10 | 7 | 0 | 0 |
| EGA | Ulz et al, 2019 | 22 | 20 | 38 | 11 |
| EGA | Zviran et al, 2020 | 38 | 33 | 3 | 0 |
| EGA | Peneder et al, 2021 | 22 | 72 | 25 | 29 |
| FinaleDB | Jiang et al, 2015 | 32 | 60 | 9 | 20 |
| FinaleDB | Cristiano et al, 2019 | 260 | 159 | 13 | 59 |

**Table S6** cfDNA samples quantification by dPCR of human RPP30 locus and also by Agilent cfDNA TapeStation.

| Specimen | Extraction Repeat | dPCR(AC/ul) | Tapestation conc/ %cfDNA |
| --- | --- | --- | --- |
| PPC10a | 1 | 179 | 276pg/ul 83% |
| PPC10b | 1 | 162 | 303pg/ul 86% |
| PPC10c | 1 | 74 | 145 pg/ul 84% |
| PPC10d | 1 | 159 | 217 pg/ul 75% |
| PPC10e | 1 | 210 | 423pg/ul 92% |
| PPC10f | 1 | 333 | 487pg/ul 90% |
| PPC10g | 1 | 74 | 117pg/ul 86% |
| PPC10h | 1 | 138 | 268pg/ul 83% |
| PPC10i | 1 | 110 | 195pg/ul 83% |
| PPC10j | 1 | 208 | 319pg/ul 88% |
| PPC10a | 2 | 87 | 260pg/ul 77% |
| PPC10b | 2 | 108 | 218pg/ul 79% |
| PPC10c | 2 | 62 | 140pg/ul 82% |
| PPC10d | 2 | 108 | 148pg/ul 74% |
| PPC10e | 2 | 195 | 230pg/ul 84% |
| PPC10f | 2 | 151 | 193pg/ul 84% |
| PPC10g | 2 | 59 | 105pg/ul 77% |
| PPC10h | 2 | 77 | 125pg/ul 76% |
| PPC10i | 2 | 64 | 157pg/ul 80% |
| PPC10j | 2 | 185 | 428pg/ul 89% |

**Table S7** The extension temperature and time of each library kit.

| Library kit | Extension temp(°C) | Extension time(s) |
| --- | --- | --- |
| XTHS | 72 | 60 |
| XTHS2 | 72 | 60 |
| PlasmaSeq | 72 | 50 |
| Tag_seq | 72 | 50 |
| Taq_seq_HV | 65 | 75 |
| EM_seq | 65 | 60 |
| Watchmaker | 72 | 30 |
| KAPA_HyperPrep | 72 | 30 |
| NEBNext_Ultra_II | 65 | 75 |
